# The functional form of specialised predation affects whether Janzen–Connell effects can prevent competitive exclusion

**DOI:** 10.1111/ele.14014

**Published:** 2022-04-26

**Authors:** Daniel J. B. Smith

**Affiliations:** ^1^ Committee on Evolutionary Biology University of Chicago Chicago Illinois USA

**Keywords:** coexistence theory, conspecific negative density dependence, Janzen–Connell effects, Janzen–Connell hypothesis, plant–soil feedbacks, species diversity, species richness, tropical forests

## Abstract

Janzen–Connell effects (JCEs), specialised predation of seeds and seedlings near conspecific trees, are hypothesised to maintain species richness. While previous studies show JCEs can maintain high richness relative to neutral communities, recent theoretical work indicates JCEs may weakly inhibit competitive exclusion when species exhibit interspecific fitness variation. However, recent models make somewhat restrictive assumptions about the functional form of specialised predation—that JCEs occur at a fixed rate when offspring are within a fixed distance of a conspecific tree. Using a theoretical model, I show that the functional form of JCEs largely impacts their ability to maintain coexistence. If predation pressure increases additively with adult tree density and decays exponentially with distance, JCEs maintain considerably higher species richness than predicted by recent models. Loosely parameterising the model with data from a Panamanian tree community, I elucidate the conditions under which JCEs are capable of maintaining high species richness.

## Graphical abstract

Recent theory challenges the efficacy of Janzen Connell Effects in maintaining species diversity. I examine how modeling assumptions affect these results. I find integrating more realistic assumptions into the model permits greater diversity maintenance than recent results indicate

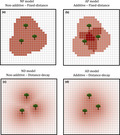

## INTRODUCTION

The Janzen–Connell hypothesis is a species coexistence mechanism frequently invoked to explain the high diversity of tropical forests (Connell, 1971; Janzen, 1970; Terborgh, 2012; Wright, 2002). It is based on the observation that specialised natural enemies (e.g. insects, fungi and pathogens) reduce the survivorship of seeds and seedlings when they are near conspecific adult trees. These phenomena are frequently referred to as Janzen–Connell effects (JCEs). JCEs are thought to promote coexistence by generating negative frequency dependence: the more common a species is, the greater the proportion of the environment its offspring experience JCEs. However, the efficacy of this mechanism remains contested on theoretical grounds.

Empirical evidence supports the presence of JCEs in a variety of systems (e.g. Bever et al., 2015; Comita et al., 2014; Hazelwood et al., 2021; Hyatt et al., 2003; Johnson et al., 2012; Mangan et al., 2010; Petermann et al., 2008; Swamy & Terborgh, 2010). Theoretical work demonstrates JCEs can effectively delay extinction from ecological drift and maintain high species richness relative to neutral communities (Adler & Muller‐Landau, 2005; Armstrong, 1989; Levi et al., 2019; Sedio & Ostling, 2013). However, for a stabilising mechanism to maintain coexistence, it must be sufficiently strong to offset interspecific fitness differences (Chesson, 2000). Recent studies that integrate interspecific variation into JCE models call into question their ability to promote deterministic coexistence (Cannon et al., 2021; Hülsmann et al., 2020; Stump & Comita, 2018) causing some to label JCEs ‘a weak impediment to competitive exclusion’ (Chisholm & Fung, 2020).

**FIGURE 1:**
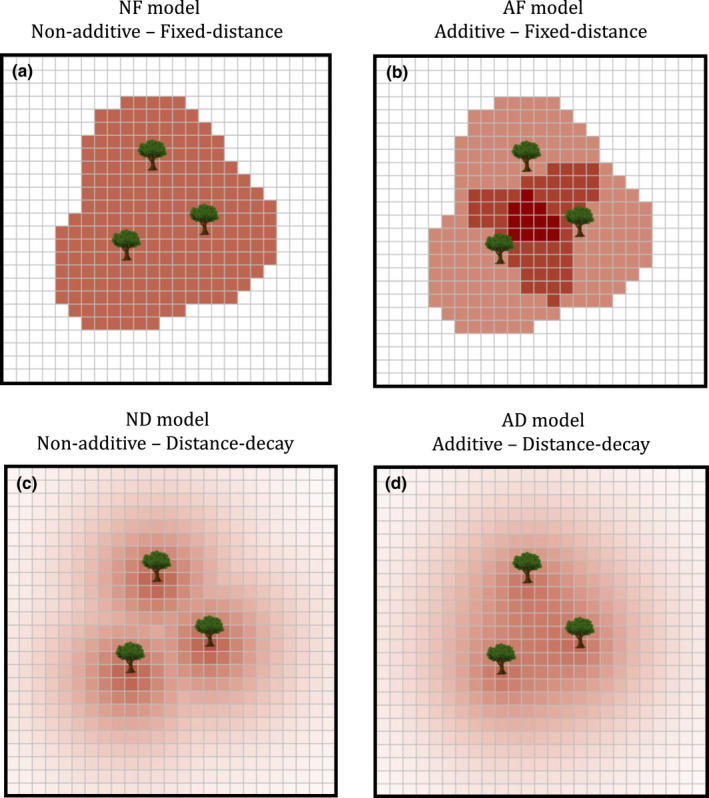
Visualisation of how each Janzen–Connell effect (JCE) functional form induces offspring mortality in 2D space. Each grid cell represents a patch on which an adult persists. For simplicity, only three adults of a single species are depicted. Shading depicts the relative probability of offspring mortality in space. Red shading corresponds to higher offspring mortality; white corresponds 100% offspring survival. The plots highlight the distinct features of each modelling assumption. The fixed‐distance models (NF and AF models; panels a and b) induce concentrated predation pressure over a relatively small area while the distance‐decay models (ND and AD; panels c and d) distribute less concentrated predation pressure over a larger area. The non‐additive models (NF and ND; panels a and c) induce relatively strong predation pressure near adults that does not increase with adult density whereas the additive models (AF and AD; panels b and d) exhibit the highest mortality where multiple conspecific adults are close in proximity. The images depict when v=10, $r=10\sqrt{2}$, g=0.172, N=250, $a_{A}=0.5$, and $a_{n}=a_{A}+a_{A}E/N$ where $E=E_{F}=E_{D}=2\pi v^{2}g$ (as per the additive–non‐additive normalisation; see ‘Model normalisations’ and Appendix E)

A notable assumption of several recent JCE‐type models (e.g. Chisholm & Fung, 2020; Levi et al., 2019) is the functional form of specialised predation pressure. These studies assume that JCEs impact offspring within a fixed distance of a conspecific adult tree. Where JCEs induce offspring mortality, they reduce survivorship by a fixed proportion that is independent of conspecific adult density. I refer to this as the ‘non‐additive‐fixed‐distance’ (NF) model (Figure 1a). The NF model is likely used because it decreases model complexity and requires relatively low computational power. However, empirical evidence indicates that offspring mortality increases additively with conspecific adult density and declines monotonically with conspecific distance (e.g. Comita et al., 2010, 2014; Hubbell et al., 2001; Johnson et al., 2014; Liu et al., 2015). I refer to this as the ‘additive‐distance‐decay’ (AD) model (Figure 1d). Several previous studies use this functional form (e.g. Adler & Muller‐Landau, 2005; Muller‐Landau & Adler, 2007; Sedio & Ostling, 2013; Stump & Comita, 2020). If offspring mortality increases with the local abundance of natural enemies that disperse from nearby conspecific trees, this model captures more biological realism.

There are two axes on which the aforementioned functional forms vary. In terms of adult density, predation pressure acts either additively or non‐additively. In terms of distance, predation occurs over a fixed distance or decays monotonically. To tease out the effect each functional form assumption has on species coexistence, I develop an ordinary differential equation (ODE) approximation of each of the four possible spatially explicit JCE models that incorporate these functional form assumptions (Figure 1). I use the ODEs to compare the relative ability of each JCE functional form to promote deterministic coexistence in a community exhibiting interspecific fitness variation. Overall, I show that the functional form of specialised predation strongly affects the ability of JCEs to maintain species richness. This study highlights the need to more precisely determine the functional form of specialised predation, quantify the parameters that affect its strength and better integrate these empirical results into theoretical models.

## MODEL AND METHODS

I consider a tree community of N species that contains M patches in which the center of every patch contains a single adult tree. Below, I describe the discrete‐time spatially explicit model.

### Offspring (within‐patch) dynamics

Each tree produces a set number of seeds each time‐step. All trees uniformly disperse a portion of their seeds (D) among patches and retain the remaining portion of their seeds (1‐D) on the local patch. The number of offspring species i disperses to each patch is proportional to $Y_{i}$, henceforth intrinsic fitness (a composite parameter of fecundity and offspring survival). On each patch, JCEs kill offspring. Let $J_{i,k}\left(x\right)$ define the probability an offspring of species i survives JCEs on a patch occupied by species k at location x. Then, letting $S_{i,k}\left(x\right)$ represent the number of offspring of species i on a patch occupied by species k at a location x:

(1) $$\begin{array}{cc} S_{i,i}\left(x\right) & \;=Y_{i}\left[\left(1‐D\right)+\;p_{i}D\right]J_{i,i}\left(x\right)\\ S_{i,k}\left(x\right) & \;=Y_{i}p_{i}DJ_{i,k}\left(x\right)\\ S_{all,i}\left(x\right) & \;=\sum_{n=1}^{N}S_{n,i}\left(x\right) \end{array}$$

where $p_{i}$ is the proportion of species i in the population, i=1,2,...,N. i≠k. If the adult on the patch at location x dies during the time‐step, a lottery determines which species replaces the adult (see ‘Tree dynamics’). If the adult survives, all offspring on the patch die. Appendix E provides a more detailed description.

### Janzen–Connell effect functional forms

#### Non‐additive‐Fixed‐distance model

JCEs kill a fixed proportion of a species' offspring when they are within r metres of a conspecific adult (Figure 1a):

(2) $$\begin{array}{c} \begin{array}{c} J_{i,i}\left(x\right)=\exp\left[‐a\right]\\ J_{i,k}\left(x\right)=\left\{\begin{array}{cc} 1, & \text{if}\;\text{min}\left(x_{i}\right)>r\\ \exp\left[‐a\right], & \text{if}\;\text{min}\left(x_{i}\right)\leq r \end{array}\right. \end{array} \end{array}$$

where $\min(x_{i})$ is the minimum distance between of an adult of species i and a patch at location x. a represents baseline predation pressure (a composite trait of predation rate and the time over which predation occurs; see Appendix E). I assume a does not vary between species.

### Additive‐fixed‐distance model

JCEs occur over a fixed radius, r, in which predation pressure increases linearly with the number of conspecific adults (Figure 1b):

(3) $$\begin{array}{c} \begin{array}{cc} J_{i,i}\left(x\right) & \;=\exp\;\left[‐a\left(1+\sum_{m\in r}{𝟙}_{m}\left(i\right)\right)\right]\\ J_{i,k}\left(x\right) & \;=\exp\;\left[‐a\sum_{m\in r}{𝟙}_{m}\left(i\right)\right] \end{array} \end{array}$$

where m∈r depicts the trees falling within the JCE radius. ${34𝟙}_{m}\left(i\right)$ is an indicator function for which ${34𝟙}_{m}\left(i\right)=1$ if m=i and ${34𝟙}_{m}\left(i\right)=0$ if m≠i.

### Non‐additive‐distance‐decay model (ND) model

Predation pressure is non‐additive and decreases exponentially with distance (Figure 1c):

(4) $$\begin{array}{cc} J_{i,i}\left(x\right) & \;=\exp\;\left[‐a\right]\\ J_{i,k}\left(x\right) & \;=\exp\;\left[‐ae^{‐\text{min}\left(x_{i}\right)/v}\right] \end{array}$$

where v defines rate at which predation declines with distance and $\min(x_{i})$ is the same as defined in the NF model.

### Additive‐distance‐decay (AD) model

Predation pressure increases linearly as a function of local conspecific density and decreases exponentially with distance (Figure 1d):

(5) $$\begin{array}{c} \begin{array}{cc} J_{i,i}\left(x\right) & \;=\exp\;\left[‐a\left(1+\sum_{m=1}^{Mp_{i}}e^{‐x_{i,m}/v}\right)\right]\\ J_{i,k}\left(x\right) & \;=\exp\;\left[‐a\sum_{m=1}^{Mp_{i}}e^{‐x_{i,m}/v}\right] \end{array} \end{array}$$

where $x_{i,m}$ is the $m_{\mathrm{th}}$ smallest distance between the focal patch (x) and an adult of species i. $Mp_{i}$ represents all trees of species i in the community. See Appendices [Link], [Link], [Link], [Link], [Link] for more details on each functional form and the modelling assumptions therein.

### Tree dynamics

Each time‐step, adult trees die with probability δ. When a tree dies, it is immediately replaced by a randomly selected offspring on the patch (a lottery model; Chesson & Warner, 1981). Let $P_{A,B}\left(x\right)$ be the probability an offspring of species A colonises a patch previously occupied by species B at location x. Then, $P_{i,i}\left(x\right)=S_{i,i}\left(x\right)/S_{all,i}\left(x\right)$ and $P_{i,k}\left(x\right)=S_{i,k}\left(x\right)/S_{all,k}\left(x\right)$. In the spatially explicit model, patches are discretised in space on a gridded torus of M=275×275 patches. For the distance‐decay models (AD and ND), the distance between two patches is defined by the Euclidean distance between their centre points. For the fixed‐distance models (AF and NF), I incorporate JCEs using Moore neighbourhoods.

I developed a deterministic ODE approximation for each spatially explicit model by taking the expected offspring abundance on each patch type. The dynamics for the proportion of species i ($p_{i}$) in the community are as follows:

(6) $$\frac{dp_{i}}{\mathrm{dt}}=\delta \left[\frac{E\left[S_{i,i}\left(x\right)\right]}{E\left[S_{all,i}\left(x\right)\right]}p_{i}+\sum_{k\neq i}\frac{E\left[S_{i,k}\left(x\right)\right]}{E\left[S_{all,k}\left(x\right)\right]}p_{k}‐p_{i}\right]$$

for which i=1,2,...,N. The first term in the parentheses is the rate at which species i recolonises patches previously occupied by conspecifics, the second term is the rate at which species i colonises patches previously occupied by species k (k≠i) and the third term is the rate at which adults of species i die.

For the NF model, offspring abundances are:

(7) $$\begin{array}{c} \begin{array}{cc} E\left[S_{i,i}\left(x\right)\right] & \;=Y_{i}\left[\left(1‐D\right)+p_{i}D\right]e^{‐a}\\ E\left[S_{i,k}\left(x\right)\right] & \;=Y_{i}p_{i}D\left[e^{‐p_{i}E_{F}}+e^{‐a}\left(1‐e^{‐p_{i}E_{F}}\right)\right] \end{array} \end{array}$$

where $E_{F}=\pi gr^{2}$, where g is density (adults per square metre). $E_{F}$ is the expected number of trees that fall within the effect radius, r. For the AF model, the offspring abundances are:

(8) $$\begin{array}{c} \begin{array}{cc} E\left[S_{i,i}\left(x\right)\right] & \;=Y_{i}\left[\left(1‐D\right)+p_{i}D\right]e^{‐a}e^{‐\left(1‐e^{‐a}\right)p_{i}E_{F}}\\ E\left[S_{i,k}\left(x\right)\right] & \;=Y_{i}p_{i}De^{‐\left(1‐e^{‐a}\right)p_{i}E_{F}} \end{array} \end{array}$$

where $E_{F}$ is the same as in the NF model. For the ND model, the offspring abundances are:

(9) $$\begin{array}{c} \begin{array}{cc} E\left[S_{i,i}\left(x\right)\right] & \;=Y_{i}\left[\left(1‐D\right)+p_{i}D\right]e^{‐a}\\ E\left[S_{i,k}\left(x\right)\right] & \;=Y_{i}p_{i}De^{‐ae^{‐\sqrt{\frac{\pi }{2E_{D}p_{i}}}}}\left(1+ae^{‐\sqrt{\frac{\pi }{2E_{D}p_{i}}}}\left(ae^{‐\sqrt{\frac{\pi }{2E_{D}p_{i}}}}‐1\right)\left(1‐\frac{\pi }{4}\right)\frac{1}{p_{i}E_{D}}\right) \end{array} \end{array}$$

where $E_{D}=2\pi gv^{2}$. $E_{D}$ relates to the predation pressure each tree induces. For the AD model, the seedling equations are:

(10) $$\begin{array}{c} \begin{array}{cc} E\left[S_{i,i}\left(x\right)\right] & \;=Y_{i}\left[\left(1‐D\right)+p_{i}d\right]e^{‐a}e^{‐ap_{i}E_{D}H\left(a\right)}\\ E\left[S_{i,k}\left(x\right)\right] & \;=Y_{i}p_{i}De^{‐ap_{i}E_{D}H\left(a\right)} \end{array} \end{array}$$

where $H\left(a\right)={}_{3}F_{3}\left(1,1,1;2,2,2;‐a\right)$, a generalised hypergeometric function. See Appendix [Link], [Link], [Link], [Link] for details.

### Model parameterisations

I roughly parameterised several key quantities using data from the Barro Colorado Island (BCI) forest plot in Panama. g (adult density) can be estimated by dividing the total number of individuals in the community by its area in square metres. The plot at BCI is 50‐ha and contains approximately 86,069 individuals of reproductive diameter based on the 1995 BCI census (Chisholm & Fung, 2020; Condit et al., 2019). This yields g≈0.172 adults per square metre.

Comita et al. (2010) estimated a distance‐decay parameter at BCI (β) similar to v, finding best and second best fit values equivalent to v=5 and v=10, respectively. β differs from v in that it describes the distance decay of consepcific adult basal area on seedling survivorship based on a GLM using a logit link function, but they are conceptually similar. To examine a range of scenarios loosely based on this measurement, I examined v between 2.5 and 15.

Chisholm and Fung (2020) found interspecific fitness variation (Y) at BCI to be log‐normally distributed with interspecific variation approximately equivalent to $Y∼\;\mathrm{lognormal}\;[\mu =0,\sigma_{Y}=1.0]$. I examined *σ*_*Y*_ between 0.1 and 1.0. Note that the nature of the lottery model (in which one species always wins the lottery) means that only relative, rather than absolute, values of intrinsic fitness matter.

I examined several values of a (baseline predation pressure). In every model, $1‐e^{‐a}$ corresponds to the probability an offspring dies due to JCEs when it is on a patch occupied by a conspecific. I henceforth frame a in terms of $1‐e^{‐a}$ because it is easy to interpret. I examined when $1‐e^{‐a}=0.4$, 0.7 and 0.99, which encompasses when JCEs range from moderately strong to very strong.

### Model normalisations

To compare models, I perform two intermodel normalisations that equalise predation pressure across distance‐dependent and density‐dependent modelling assumptions. See Appendix E for full details.

To normalise distance‐decay and fixed‐distance functional forms, I equalise the predation pressure each individual tree induces over space. Let G(x) represent the relative predation pressure induced x metres away from a single adult tree (G(x)≤1). The total predation pressure induced by a single tree in two‐dimensional space is $2\pi g\int_{0}^{\infty }xG\left(x\right)dx$. For the distance‐decay models (predation declines exponentially with distance) $G\left(x\right)=e^{‐x/v}$, giving $2\pi g\int_{0}^{\infty }xe^{‐x/v}dx=2g\pi v^{2}$. For the fixed‐distance models (predation occurs within a fixed area, r), G(x)=1 for x≤r and G(x)=0 if x>r, giving $2\pi g\int_{0}^{r}xdx=g\pi r^{2}$. Setting these values equal yields $r=v\sqrt{2}$ (which also implies $E_{F}=E_{D}$). This normalisation is similar to methods used in previous models (Adler & Muller‐Landau, 2005; Sedio & Ostling, 2013) and can be interpreted as equalising the total number of predators that disperse from trees (Appendix E).

Second, I normalise additive and non‐additive models. To do so, I modify the baseline predation pressure of the non‐additive models such that additive and non‐additive models exhibit the same mean predation. Let E represent either $E_{D}$ or $E_{F}$ (which are equal under the first normalisation). On a random patch in a community of N species, it can be shown that additive predation increases mean predation pressure by aE/N relative to when predation is non‐additive. This is derived by taking the expected predation pressure a species' offspring experiences on a random patch in the community (see Appendix E). Then, letting $a_{n}$ and $a_{A}$ be the baseline predation pressure of the non‐additive and additive models, respectively, mean predation pressure is equal between models if $a_{n}=a_{A}+a_{A}E/N$. I henceforth use the notation $a_{A}$ when referring to baseline predation pressure.

### Presentation of results

#### Ordinary differential equation (ODE) analysis

Using the ODE model (Equation 6), I ran simulations with the above‐mentioned parameterisations and normalisations under different levels of dispersal limitation (D) to compare how each functional form maintains species richness. The initial number of species for each simulation was set to 300. Three hundred was selected on the basis that the forest plot at BCI contains approximately 300 woody plant species (Condit et al., 2019). Simulations were run for 10,000 generations, more than sufficient for the system to reach equilibrium (Appendix G, Figure G3). I considered species i to be extinct if $\log p_{i}<‐11$ (recalling $p_{i}$ the proportion of species i). This approximately corresponds to less than one individual at the BCI forest plot (log1/86,006≈‐11). The additive–non‐additive normalisation was implemented by running a simulation with additive models and using the number of species maintained to normalise baseline predation. For example, if the AD model maintained N species, then the NF and ND models were normalised by setting $a_{n}=a_{A}+a_{A}E_{D}/N$. Simulations were performed in R (R Core Team, 2020) using the package deSolve (Soetaert et al., 2010).

I performed additional analyses to explain the simulation outputs. First, I examined how each functional form induces negative frequency dependence by analysing Equations (7), (8), (9), (10). Then, I analysed approximate invasion criteria for each model. The invasion criteria quantify the minimum fitness that permits the invasion of a rare species in a community of N residents under the assumption of global dispersal (D=1; Table 1; Appendices [Link], [Link], [Link], [Link]).

**TABLE 1:** Model types, their abbreviations and their approximate invasion criterion. The right‐hand side of each approximate invasion criterion quantifies the minimum fitness of a rare species, $Y_{i}$, required for invasion (deterministic increase in abundance). I refer to this as the ‘minimum invader fitness’. $\bar{Y}=\frac{1}{N}\sum_{k=1}^{N}Y_{k}$. $\Gamma^{*}$ is a large and complicated expression. See Appendix C for the full expression and Appendices [Link], [Link], [Link], [Link] for derivations

Model	Abbreviation	Approximate invasion criteria
Non‐additive‐fixed‐distance	NF	$Y_{i}>{\underbrace{\bar{Y}\left(1‐\left(1‐e^{‐a}\right)\left(1‐e^{\frac{‐E_{F}}{N}}\right)\right)}}_{\text{mean JCE}‐\text{fitness}\text{term}}+{\underbrace{N\text{Cov}\left(p,Y\right)\left(e^{‐\frac{E_{F}}{N}}\left(1‐e^{‐a}\right)\left(1‐\frac{E_{F}}{N}\right)+e^{‐a}\right)}}_{\text{covariance}‐\text{JCE}\text{term}}$
Additive‐fixed‐distance	AF	$Y_{i}>{\underbrace{\bar{Y}e^{‐\left(1‐e^{‐a}\right)\frac{E_{F}}{N}}}}_{\text{mean JCE}‐\text{fitness}\text{term}}+{\underbrace{N\text{Cov}\;\left(p,Y\right)\left(1‐\left(1‐e^{‐a}\right)\frac{E_{F}}{N}\right)e^{‐\left(1‐e^{‐a}\right)\frac{E_{F}}{N}}}}_{\text{covariance}‐\text{JCE}\text{term}}$
Non‐additive‐distance‐decay	ND	$Y_{i}>{\underbrace{\bar{Y}e^{‐ae^{‐\sqrt{\frac{\pi N}{2E_{D}}}}}\left(1+ae^{‐\sqrt{\frac{\pi N}{2E_{D}}}}\left(ae^{‐\sqrt{\frac{\pi N}{2E_{D}}}}‐1\right)\left(1‐\frac{\pi }{4}\right)\frac{N}{E_{D}}\right)}}_{\text{mean JCE}‐\text{fitness}\text{term}}+{\underbrace{N\text{Cov}\;\left(p,Y\right)\Gamma^{*}}}_{\text{covariance}‐\text{JCE}\text{term}}$
Additive‐distance‐decay	AD	$Y_{i}>{\underbrace{\bar{Y}e^{‐aH\left(a\right)\frac{E_{D}}{N}}}}_{\text{mean JCE}‐\text{fitness}\text{term}}+{\underbrace{N\text{Cov}\;\left(p,Y\right)\left(1‐aH\left(a\right)\frac{E_{D}}{N}\right)e^{‐aH\left(a\right)\frac{E_{D}}{N}}}}_{\text{covariance}‐\text{JCE}\text{term}}$

#### ODE validation

I ran spatially explicit and ODE model simulations using identical parameterisations and compared species richness outputs. Spatially explicit simulations were run on the 275×275 patch community until the transient dynamics had approximately concluded (after species richness approximately stabilised; Appendices [Link], [Link], [Link], [Link]). I ran simulations with $\sigma_{Y}∼\left\{0.1,0.45,0.8\right\}$ and a∼{0.5,1.0,2.75,4.5}. For each of the 12 parameter combinations, I ran simulations with model v∼{5,7.5,10} for the distance‐decay functional forms with g=0.2. For the fixed‐distance model, I ran simulations with Moore neighbourhoods ranging in size from 3×3 (small) to 11×11 (large) which corresponds to r values between 4.0‐14 for g=0.2.

## RESULTS

### Species richness maintained by each model

**FIGURE 2:**
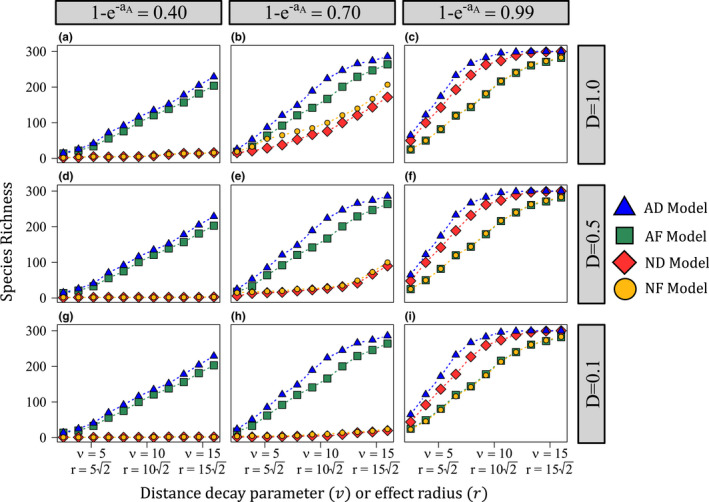
Species richness maintained by each Janzen–Connell effect (JCE) functional form under different values of baseline predation pressure ($a_{A}$), dispersal limitation (D) and the spatial scale of predation (v and r). For each plot, the x‐axis depicts either v or r (depending on the JCE functional form) and the y‐axis is species richness (the number of species maintained in the community at equilibrium). Models are differentiated by point shape and colour. Each column depicts a different value of $a_{A}$ (the baseline predation pressure for the additive models) and each row shows a different value of D (D=1, D=0.5 and D=0.1, respectively). The non‐additive models are normalised such that $a_{n}=a_{A}+a_{A}E/N_{A}$, where $a_{n}$ is the normalised predation pressure for the non‐additive models, $N_{A}$ is the diversity maintained by the additive model to which the non‐additive model is being compared and E is either $E_{F}$ or $E_{D}$ (which are equivalent). $a_{N}$ was calculated based on the diversity maintained by the AD model for the additive–non‐additive normalisations of both NF and ND models. Using AF model outputs to quantify $a_{n}$ yielded trivially similar results. All simulations were conducted with 300 species initially in the population. Parameters not noted on the figure are as follows: g=0.172 and $\sigma_{Y}=0.55$ ($Y∼\;\mathrm{lognormal}\;[\mu =0,\sigma_{Y}]$)

**FIGURE 3:**
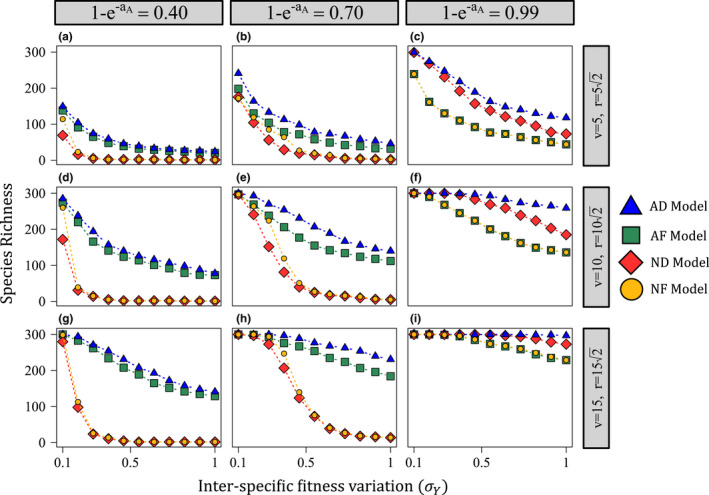
How the level of interspecific fitness variation ($\sigma_{Y}$) affects species richness for each Janzen–Connell effect (JCE) functional form ($Y∼\;\mathrm{lognormal}\;[\mu =0,\sigma_{Y}]$). For each plot, the x‐axis is $\sigma_{Y}$ and the y‐axis is species richness (the number of species maintained in the community at equilibrium). Each column depicts a different value of $a_{A}$ (the baseline predation pressure for the additive models) and each row shows a different value of v or r (the spatial scale of predation). Additive– non‐additive normalisations were calculated using the same method noted in Figure 2. For lower $a_{A}$ (columns 1 and 2), the additive models (AD and AF) are much more robust to interspecific fitness differences ($\sigma_{Y}$), particularly over larger spatial scales (rows 2 and 3). For large $a_{A}$ (column 3), distance‐decay models (AD and ND) are more robust to interspecific fitness differences than the fixed‐distance models. All simulations were conducted with 300 species initially in the population. Parameters not noted on the figure are as follows: g=0.172 and D=0.5

For all models, species richness increased with baseline predation pressure ($a_{A}$) and the spatial scale of predation (v and r; Figures 2 and 3) and decreased with interspecific fitness variation ($\sigma_{Y}$; Figure 3). The AD model promoted the highest species richness in all cases. Which model promoted the second highest species richness depended on $a_{A}$. For low to moderate $a_{A}$, the AF model maintained the second greatest species richness (Figures 2 and 3, columns 1 and 2). In these cases, the species richness maintained by the non‐additive models (ND and NF) declined rapidly with increasing $\sigma_{Y}$ (Figure 3, columns 1 and 2). While species richness also decreased with $\sigma_{Y}$ for the additive models (AD and AF), the decline was much less pronounced (particularly for large v and r). For large $a_{A}$, the ND model maintained the second highest diversity (Figures 2 and 3, column 3). While all models were capable of maintaining somewhat high species richness for large $a_{A}$, the distance‐decay models (AD and ND) were more robust to interspecific fitness variation than the fixed‐distance models (Figure 3, column 3).

The initial pool of 300 species limited species richness when JCEs were strong (e.g. Figure 2, column 3; Figure 3i). With a larger initial species pool (1000 species, similar to the number of trees in central Panama; Condit et al., 2013), differences in species richness maintained by each model were considerably more pronounced in these cases (Appendix G, Figures G4 and G5). Therefore, Figures 2 and 3 may understate model differences.

Dispersal limitation trivially affected the additive models, but decreased species richness for the non‐additive models (Figure 2; Appendix G, Figure G4). This can be understood as follows. In the absence of dispersal limitation (D=1), a rare species suffers no offspring mortality due to JCEs; when D<1, a rare species' locally dispersed offspring suffer JCE‐induced mortality. When predation is non‐additive, a rare species' locally dispersed offspring experience the same mortality as those of resident species. When predation is additive, resident species' locally dispersed offspring always experience greater mortality than those of rare species due to additive effects. Thus, dispersal limitation decreases rare species advantage when predation is non‐additive but not when it is additive. Dispersal limitation most strongly affected species richness for intermediate $a_{A}$. It can be shown that the dispersal limited and non‐dispersal limited cases converge when $a_{A}$ is very large or small (Appendix F).

### Mechanisms of diversity maintenance

The AD model always promotes the highest species richness, the AF model promotes the second highest species richness for lower baseline predation pressure ($a_{A}$), and the ND model promotes the second highest species richness for large $a_{A}$ (Figures 2 and 3; Appendix G, Figures G4 and G5). Below, I explain these results.

**FIGURE 4:**
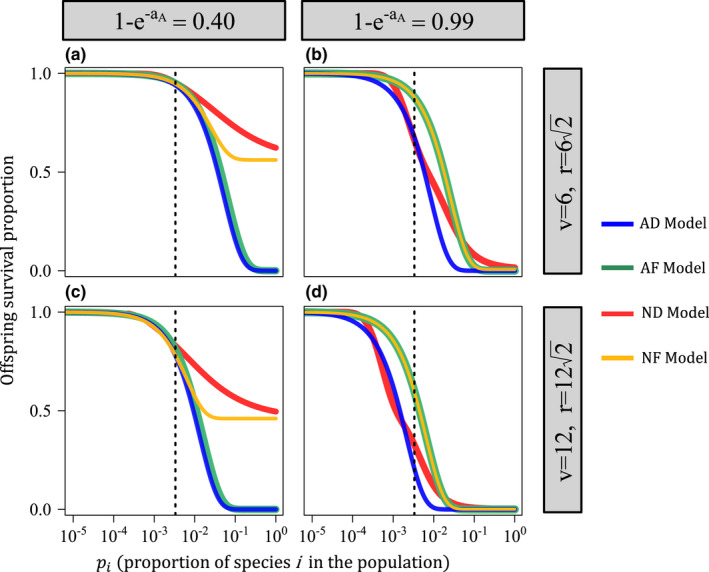
How offspring survivorship scales with conspecific adult proportion for each functional form. For each plot, the x‐axis is the proportion of adults of a focal species in the population ($p_{i}$) and the y‐axis is the expected proportion of surviving offspring on random patch occupied by a heterospecific. These values are taken from Equations (7), (8), (9), (10). For the AD model, for example (Equation 10): $E\left[S_{i,k}\left(x\right)\right]=Y_{i}p_{i}De^{‐a_{i}p_{i}E_{D}H\left(a_{i}\right)}$. The y‐axis of this figure is $e^{‐a_{i}p_{i}E_{D}H\left(a_{i}\right)}$, which is equivalent to $E[J_{i,k}\left(x\right)]$ (see Appendices [Link], [Link], [Link], [Link]). The other curves show the analogous terms from Equations (7), (8), (9). Models are indexed by colour. Each model assumes N=300; the dotted lines depict 1/N, the mean proportion of the population. Columns show different values of $1‐e^{‐a_{A}}$ (baseline predation pressure; 0.4 and 0.99, respectively). Rows show different values of v (5 and 10, respectively). First, consider low $1‐e^{‐a_{A}}$ (column 1; a and c). When $p_{i}$ is low (on the order of 1/N) all models produce similar offspring survival. When $p_{i}$ is large, the additive models (AD and AF; blue and green curves) produce lower offspring survival than the non‐additive models (ND and NF). Now, consider high $1‐e^{‐a_{A}}$ (column 2; b and d). All models produce low offspring survival when species i is common (large $p_{i}$). When $p_{i}$ is small (on the order of 1/N, the mean proportion) the distance‐decay models (AD an ND; blue and red curves) induce much lower survival than the fixed‐distance models. Note that the fixed‐distance (AF and NF) models converge for large $a_{A}$ (the orange and green curves are essentially identical). This is because nearly all offspring die if a conspecific adult is found with the effect area, in which case additive predation does not meaningfully increase offspring mortality. Parameters are as follows: g=0.172, D=1.0, N=300 and $a_{n}=a_{A}(1+E/N)$ where $E=E_{F}=E_{D}=2\pi v^{2}g$

Examining Equations (7), (8), (9), (10), the proportion of surviving offspring of species i on a random patch declines with increasing conspecific adult proportion in the community ($p_{i}$) for each model (Figure 4). The nature of this decline, which reflects the strength of negative frequency dependence, depends on whether predation is additive or non‐additive. When predation is additive (AD and AF models), offspring survival declines monotonically with $p_{i}$; when predation is non‐additive (ND and NF models), offspring survival decreases as a saturating function of $p_{i}$ (saturating to the fixed value set non‐additive predation pressure, $a_{n}$; Figure 4). This difference between additive and non‐additive functional forms is pronounced when $a_{A}$ is relatively small (Figure 4a,c). For large $a_{A}$, all models strongly reduce offspring survivorship when a species is common (Figure 4b,c), though still more so for the additive models (Appendix G, Figure G6). However, under large $a_{A}$, when species proportion is near the population mean ($p_{i}\approx 1/N$), the distance‐decay (AD and ND) models induce lower offspring survivorship than the fixed‐distance (AF and NF) models (Figure 4b,d; dotted lines). Thus, additive predation induces relatively strong negative frequency dependence for small $a_{A}$ while the distance‐decay functional form produces lower mean offspring survival for large $a_{A}$.

The invasion criteria quantify how these differences affect the growth rate of a rare species. The right hand side of each invasion criterion (henceforth the ‘minimum invader fitness’; Table 1) quantifies the minimum fitness required for a rare species to invade a community of N residents. The minimum invader fitness is broken into two parts. (1) The mean–JCE–fitness term, which consists of the product of the mean fitness of the resident community ($\bar{Y}$) and the average proportion of offspring that survive JCEs in the resident community (the latter component being equivalent to the dotted lines in Figure 4). This term quantifies the mean JCE‐scaled fitness of the resident species that an invader competes against. (2) The covariance‐JCE term, which consists of two components: the covariance between species proportion and intrinsic fitness (Cov(p,Y)) and several coefficients. Because species differ only in intrinsic fitness, Cov(p,Y) is positive (fitter species are more common). The coefficients, in part, quantify how JCEs reduce the offspring survivorship of relatively common species. The covariance–JCE term therefore quantifies the amount of competition the invader faces from relatively fit and common species; lower values correspond to stronger negative frequency dependence.

**FIGURE 5:**
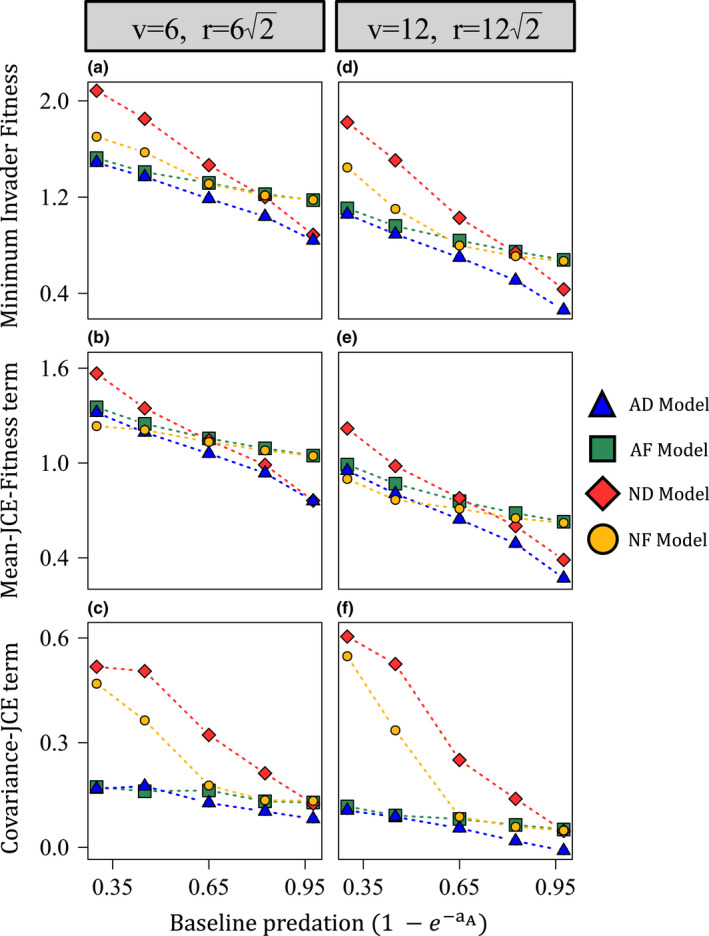
How the different functional forms promote invasion as defined by their invasion criteria quantified from simulated communities. All simulations began with 300 species. First, additive model simulations were run and normalisations were performed as described in the main text, Figure 2 and Appendix E. Simulations were run until each model reached equilibrium. Using the equilibrium values, the invasion criteria components were calculated for each model. Each row shows a different component of the invasion criteria. (a–c) show v=6 ($r=6\sqrt{2}$) and (d–f) show v=12 ($r=12\sqrt{2}$). The x‐axis of each plot shows the baseline predation pressure for the additive models ($1‐e^{‐a_{A}}$). (a, d) show the minimum invader fitness for each invasion criteria (i.e. the right‐hand side of the invasion criteria), (b, e) show the mean–Janzen–Connell effect (JCE)–fitness term, and (c, f) show the covariance–JCE term. Values for each model in (a) are the sum of values (b) and (c); values for each model in (d) are the sum of values in (e) and (f). The key results are as follows: when $a_{A}$ is small, the additive (AD and AF) models yield much lower covariance–JCE terms than the non‐additive (ND and NF) models. Hence, the additive models produce lower minimum invader fitness than the non‐additive models. When $a_{A}$ is large, the distance‐decay (AD and ND) models induce lower mean–JCE fitness terms than the fixed‐distance (AF and NF) models. Hence, the distance‐decay models produce lower minimum invader fitness than the fixed‐distance models. Parameters are as follows: D=1, g=0.172 and $\sigma_{Y}=0.375$. Appendix G, Figure G4, shows how the exact invasion criteria values (the minimum invader fitness values) compared to the approximations (a, d). Outputs are qualitatively similar

Examining the invasion criteria through numerical simulations, the AD model exhibited the lowest minimum invader fitness in all cases (Figure 5). For relatively weak predation pressure (low $a_{A}$), the AF model exhibited the second lowest minimum invader fitness whereas the non‐additive models (ND and NF) produced relatively high invader minimum fitness, especially for larger v and r (Figure 5a,d). The covariance–JCE terms of the non‐additive (ND and NF) models were much greater those of their additive counterparts while mean–JCE terms were similar, indicating the strength of negative frequency dependence underlies model differences for low $a_{A}$ (Figure 5c,f). When $a_{A}$ was large, the ND model produced the lowest minimum invader fitness (Figure 5a,d). In this case, the mean–JCE–fitness terms of the distance‐decay (AD and ND) models were considerably lower than those of the fixed‐distance (AF and NF) models whereas the covariance–JCE terms were similar. This indicates that mean offspring survival underlies model differences for large $a_{A}$ (Figure 5b and e; c and f). These results are consistent with the above analysis (Figure 4) and species richness outputs (Figures 2 and 3).

### ODE validation

The ODE model yielded species richness outputs highly similar to the spatially explicit models. Across simulations, the mean difference in species richness between the spatially explicit models and ODEs for each functional form was less than 3.5 species. The $r^{2}$ (coefficient of determination) for species richness between spatially explicit and ODE models was greater that 0.98 for all functional forms. (Appendices [Link], [Link], [Link], [Link]; see Appendix G, Figure G1 for a summary figure). Results for Shannon diversity were similar (Appendix G, Figure G2).

## DISCUSSION

### How the functional form of specialised predation affects species richness

In this paper, I demonstrate that the functional form of specialised predation strongly affects the ability of JCEs to inhibit competitive exclusion. This is important in the context of recent modelling studies which indicate JCEs are unable to maintain diversity if species exhibit interspecific fitness variation (e.g. Chisholm & Fung, 2020; Stump & Comita, 2018). These and similar studies (Levi et al., 2019) assume that specialised predation pressure affects offspring at a fixed rate within a discrete distance of a conspecific tree (the NF model; Figure 1a). Such functional forms leave out biological details as an approximation; previous work indicates that JCEs increase additively with adult density and decay monotonically with distance (the AD model; Comita et al., 2010; Hubbell et al., 2001). To investigate how the functional form of specialised predation affects competitive outcomes, I developed four models that utilise these combinations of assumptions (the AD, ND, AF and NF models). Overall, I find the AD model (the model with the greatest biological complexity) promotes the highest species richness.

The importance of each functional form assumption depended on the parameter space. When baseline predation pressure was relatively weak (low $a_{A}$), additive predation produced communities much more robust to high interspecific fitness variation than non‐additive predation (Figures 2 and 3; Appendix G, Figures G4 and G5). This result reflects how additive predation produces relatively strong negative frequency dependence (Figure 4) and limits how much competition invaders experience from highly fit species (Figure 5c,f). Notably, offspring recruitment near conspecific adults is likely not uncommon for low $a_{A}$. Therefore, contrary to previous arguments (Chisholm & Fung, 2020), the close proximity of conspecific adults is not incompatible with the operation of strongly stabilising JCEs if predation is additive.

For large $a_{A}$, the distance‐decay (AD and ND) models maintained higher diversity than the fixed‐distance (AF and NF) models. The fixed‐distance models induce concentrated predation pressure within a highly localised area while the distance‐decay models spread less concentrated predation over a wider space (Figure 1) which can generate lower mean offspring survival (Figures 4b,d and 5b,e). Therefore, contrary to how JCEs are often conceptualised (Terborgh, 2012), JCEs maintain higher species richness when predation pressure is less (rather than more) localised (conceptually similar to Stump & Chesson, 2015).

Results also suggest that dispersal limitation decreases species richness when predation is non‐additive but not when additive (Figure 2). This asymmetry helps to explain seemingly inconsistent results of previous JCE‐type models. Models that consider non‐additive predation find dispersal limitation decreases species richness (Chisholm & Fung, 2020; Stump & Chesson, 2015) whereas several studies using additive functional forms indicate dispersal limitation can increase species richness (Adler & Muller‐Landau, 2005; Detto & Muller‐Landau, 2016; Wiegand et al., 2021). Although Stump and Chesson (2015) argue that the combination dispersal limitation and localised predation decreases the stabilising effects of JCEs, results suggest this is the case only when predation is non‐additive (see Appendix F).

### Empirical measurements in relation to the model

The parameter $a_{A}$ encapsulates the sum of all JCE‐related mortality that occurs throughout juvenile development. Careful long‐term measurements of inter‐life history stage interactions are necessary to quantify the probability of conspecific recruitment. Meta‐analyses, such as Comita et al. (2014), indicate adult effects on conspecific seedlings are fairly strong. Song et al. (2021), examining 52 tree species across latitudes, found that seedlings near conspecific adult trees experienced an average reduction in survivorship near 63% (Song et al. (2021), Figure 5). Alvarez‐Loayza and Terborgh (2011) found that seeds and seedlings near adult trees experience nearly 100% mortality in an Amazonian forest. Overall, while estimates of $a_{A}$ are inconclusive, it seems likely that JCEs are often strong enough to stabilise large fitness differences if they occur on a sufficiently large spatial scale and predation is additive.

Determining the spatial scale of specialised predation requires accurately quantifying v (or r) and g (tree density). Hubbell et al. (2001) found that negative density‐dependent effect lost significance ∼15 m away from adult trees at BCI (although negative effects were measured up 30 m away) and Comita et al. (2010) measured a parameter similar tov, for which the best and second best fits were equal to 5 and 10, respectively (as noted earlier). While more measurements are warranted, these results point toward an intermediate value of v, for which the AD model can maintain moderately high species richness, at least in tropical communities such as BCI (Figures 2 and 3, Appendix G4 and G5).

The effective spatial scale over which JCEs operate is determined by the interaction of v and density (g). If individuals are tightly packed in space (high g), predation extending a modest distance affects many patches. Consistent with this, lower species richness is maintained when models are parameterised with lower g (Appendix G, Figure G7). This suggests JCEs are more stabilising in highly dense communities, consistent with the hypothesis that JCEs are particularly important in dense, species‐rich tropical forests.

### Limitations and future directions

Results are based on ODE approximations of spatially explicit models. The ODEs examine deterministic coexistence, ignoring ecological drift. Although JCEs can stabilise against drift in otherwise neutral communities (Levi et al., 2019), interspecific variation in fitness induces variance in species' equilibrium abundances, making weaker competitors more susceptible to stochastic extinction over long time periods (Miranda et al., 2015; Nisbet & Gurney, 1982). Similarly, this paper does not consider species immigration, which May et al. (2020) suggest can drown out the signal of JCEs.

The JCE functional form comparisons of this study depend on normalisations which have built‐in assumptions. While these normalisations are biologically meaningful (Appendix E), they are not the only feasible method by which they could be performed. A better understanding of the biological processes comprising JCEs would allow for more informed comparisons.

A major simplification of this study is that species are modelled identically except for intrinsic fitness (Y). However, life history traits such as shade tolerance and mycorrhizal association affect which natural enemies attack specific host species (Jia et al., 2020) which likely determines species‐specific natural enemy dispersal (v) and baseline predation strength (Zhu et al., 2018). Interspecific variation in these traits can reduce species richness (Stump & Comita, 2018). v also likely depends on host organism size: the roots, leaf litter and natural enemies of relatively large tree species are more likely to affect larger areas. Future models should better integrate JCEs into life history theory, building on the work of Stump and Comita (2020).

Similarly, adult density (g) was calculated by dividing the number of reproductive adults at the BCI forest plot (∼86,000) by its area (50 ha). This comprises disparate lifeforms, from shrubs to canopy trees. There are fewer canopy trees than smaller plants, yet canopy trees make up a considerable proportion of community DBH (diameter at 1.3 m above ground; Comita et al., 2007). Therefore, g may be inflated for larger organisms. Conversely, while the calculation of g included only adults, density‐dependent interactions occur at multiple life history stages. The BCI plot contains approximately 210,000 stems with DBH ≥1 cm and many more smaller seedlings (Condit et al., 2019) which also contribute to conspecific negative density effects (Comita et al., 2010; Zhu et al., 2015). These complications make g difficult to confidently parameterise when modelling only adult–offspring interactions. Future models should incorporate organism size structure and interactions between multiple plant life history stages.

Assessing the importance of JCEs also requires a better characterisation of distance and density‐dependent predation. A key factor to determine is the extent to which predation is additive. This paper examines when predation pressure is either non‐additive or linearly additive, which represent two non‐exhaustive baseline cases. Detto et al. (2019) found adult density effects on seedling survival to be additive, but sub‐linearly so. Empirical research should emphasise measuring the precise functional form and theoretical studies should quantify the impact of sub‐linear density effects on species richness.

## CONCLUSION

Unravelling the importance of JCEs in species‐rich communities remains a difficult empirical and theoretical challenge. It is my hope that this paper will motivate more precise empirical measurements of the functional form of specialised predation and motivate theoreticians to more critically examine how modelling assumptions affect distance‐ and density‐dependent processes.

## AUTHORSHIP

DJBS conceived the study, performed all analyses and wrote the paper.

### PEER REVIEW

The peer review history for this article is available at https://publons.com/publon/10.1111/ele.14014.

## Supporting information

Supplementary Material

Supplementary Material

Supplementary Material

Supplementary Material

Supplementary Material

Supplementary Material

Supplementary Material

## Data Availability

No new data were used in this study. The code to reproduce the figures from the main text and appendices is available at the Dryad Digital Repository (https://doi.org/10.5061/dryad.vhhmgqnw7) and Zenodo (https://doi.org/10.5281/zenodo.6462743).
