# Supplementary material for: The functional form of specialised predation affects whether Janzen–Connell effects can prevent competitive exclusion
Source: Ecol Lett. 2022 Apr 26;25(6):1458–70. doi: 10.1111/ele.14014 (PMC9324109; doi:10.1111/ele.14014)
Supplement: Supplementary file 1 — Supplementary Material [file ELE-25-1458-s002.pdf]

Appendix A: Non-additive−Fixed-distance model SEM and ODE approximation

Contents

1 Introduction **2**

2 SEM and derivation of ODE approximation **2**

2.1 Spatially Explicit Model (SEM) 2

2.2 Ordinary Differential Equation (ODE) Model 4

3 Comparison between ODE model and SEM **6**

3.1 ODE and SEM parameterization 6

3.2 Results of comparison 7

4 Derivation of invasion criteria **8**

5 Note on ODE approximation **11**

6 Figures **13**

1 Introduction

In this Appendix, I derive and analyze the non-additive−Fixed-distance (NF) model from the main text. This Appendix is composed of three sections: **(1)** I introduce a spatially explicit model (SEM) for the NF model. Then, I demonstrate that taking the expected offspring abundances on each patch yields the Ordinary Differential Equation (ODE) model discussed in the main text. **(2)** To asses the accuracy of the ODE approximation, I provide outputs of the ODE model and the SEM model under identical parameterizations. I show the outputs are highly similar, hence demonstrating that the ODE is a sufficiently accurate approximation of the SEM. **(3)** I provide the derivation of the approximate invasion criteria of the NF model.

2 SEM and derivation of ODE approximation

In this section, I assume the reader is generally familiar with the model discussed in the main text. First, I discuss the SEM, briefly reviewing within-patch dynamics. Then, I show the derivation of the ODE approximation.

2.1 Spatially Explicit Model (SEM)

Using the methods similar to recent JCH-type models (Levi *et al.*, 2019; Chisholm & Fung, 2020) I developed a spatially explicit model. The model depicts a community with M total patches modeled as an $L \times L$ grid ($L \times L = L^2 = M$). A torus was used to avoid edge effects. A single tree is present on every patch of the torus. At each time-step, each tree dies with probability δ. Tree replacements occur via a lottery model determined by the relative abundances of offspring of each species on each patch. As noted in the main text, offspring abundances are defined by

$$\begin{aligned} S_{i,i}(x) &= Y_i\big[(1-D) + p_i D\big] J_{i,i}(x) \\ S_{i,k}(x) &= Y_i p_i D J_{i,k}(x) \\ S_{all,i}(x) &= \sum_{n=1}^{N} S_{n,i}(x) \end{aligned} \tag{A.1}$$

where $S_{A,B}(x)$ is the offspring abundance of species A on a patch occupied by species B at location x, $J_{i,i}(x)$ and $J_{i,k}(x)$ are how JCEs affect offspring surivorship, p_i is the proportion of species i in the population, Y_i is the intrinsic fitness of species i, and D is the dispersal proportion. For the NF model, I consider when JCEs kill a fixed proportion of a species' offspring on the nearest E_F patches as defined by the Moore neighborhood surrounding the patch. $E_F = 9$ indicates a 3×3 Moore neighborhood, $E_F = 25$ indicates a 5×5 Moore neighborhood, and $E_F = 49$ indicates a 7×7 Moore neighborhood, etc. Then,

$$J_{i,i}(x) = e^{-a_i}$$
$$J_{i,k}(x) = \begin{cases} 1, & \text{if } i \notin E_F \\ e^{-a_i}, & \text{if } i \in E_F \end{cases} \tag{A.2}$$

This yields the following offspring abundances:

$$S_{i,i}(x) = Y_i\big[(1-D) + p_i D\big]e^{-a}$$
$$S_{i,k}(x) = \begin{cases} Y_i D p_i, & \text{if } i \notin E_F \\ Y_i D p_i e^{-a}, & \text{if } i \in E_F \end{cases} \tag{A.3}$$
$$S_{all,i}(x) = Y_i\big[(1-D) + p_i D\big]e^{-a} + D\sum_{k \in E_F} Y_k p_k e^{-a} + D \sum_{j \notin E_F} Y_j p_j$$

$i \in E_F$ is the condition that an adult of species i is within the Moore neighborhood around a patch occupied by species k and $i \notin E_F$ is the condition an adult is not within the Moore neighborhood around a patch occupied by species k ($k \neq i$). Noting that I use the notation $\in E_F$ rather than $\in r$, as I do in the main text. This is simply to emphasize that the SEM uses a discrete lattice to model the area of effect, but these two notations can be used interchangeably. $E_F = \pi r^2 g$ where g is tree density in individuals per square meters. To examine parameterizations of the SEM in

terms of r, values of r must be selected such that they correspond to viable Moore neighborhood values (such that $E_F = \pi r^2 g = 9$, 25, 49, etc.).

Tree replacement is determined by a lottery model. Let $P_{A,B}(x)$ be the probability species A colonizes a patch previously occupied by species B on patch x. Then, $P_{i,i}(x) = S_{i,i}(x)/S_{all,i})x)$ and $P_{i,k}(x) = S_{i,k}(x)/S_{all,k}(x)$.

2.2 Ordinary Differential Equation (ODE) Model

To derive the ODE model, I take approximations of the expected values of $P_{i,i}(x)$ and $P_{i,k}(x)$. To do so, I take the expected abundance of $S_{i,i}(x)$, $S_{i,k}(x)$, and $S_{all,i}(x)$ and then take their quotients. Expectations are taken with respect to space. Using this, I derive the the ODE approximation

$$\frac{dp_i}{dt} = \delta\left[\frac{\mathbb{E}[S_{i,i}(x)]}{\mathbb{E}[S_{all,i}(x)]}p_i + \sum_{k\neq i}\frac{\mathbb{E}[S_{i,k}]}{\mathbb{E}[S_{all,k}(x)]}p_k - p_i\right] \tag{A.4}$$

that captures the behavior of the SEM. See "Note" at the end of this Appendix for additional information about the assumptions of this approximation.

The SEM incorporates JCEs based on the nearest E_F neighbors of each patch that, computationally, are stored in a matrix. In contrast, the deterministic ODE model is spatially implicit. Therefore, it is necessary to use an approximation of the terms that does not require spatial information. With this in mind, the quantity $S_{i,k}(x)$ must be approximated as a spatially implicit term. I accomplish this by taking the expected offspring abundance on each patch. Note that by the linearity of expectation,

$$\begin{aligned}
\mathbb{E}[S_{all,i}(x)] &= \mathbb{E}\left[\sum_{n=1}^{N} S_{n,i}(x)\right] \\
&= \mathbb{E}\left[S_{i,i}(x) + \sum_{n\neq i}^{N} S_{n,i,x}\right] \\
&= \mathbb{E}[S_{i,i}(x)] + \sum_{n\neq i}\mathbb{E}\left[S_{n,i}(x)\right]
\end{aligned} \tag{A.5}$$

Additionally, $\mathbb{E}[S_{i,i}(x)]$ does not contain spatial information, as $\mathbb{E}[S_{i,i}(x)] = \mathbb{E}[Y_i\big[(1-D)+p_iD\big]e^{-a}] = Y_i\big[(1-D)+p_iD\big]e^{-a}$. Therefore, it suffices to calculate the expectation of $S_{i,k}(x)$, from which the other terms of interest (i.e. $\mathbb{E}[S_{all,k}(x)]$) can be calculated.

The expected offspring abundance of species i on a given patch is equal to the number of offspring it would have given it is not affected by JCEs multiplied the probability it does not experience JCEs added to the number of offspring it would have given it experiences JCEs multiplied the probability it does experience JCEs:

$$\mathbb{E}[S_{i,k}(x)] = \mathrm{P}[i \notin E_F] \times S_{i,k}^{i \notin E_F}(x) + \mathrm{P}[i \in E_F] \times S_{i,k}^{i \in E_F}(x) \tag{A.6}$$

Here, I again define E_F. As noted in the main text, if the neighborhood is of radius r meters and trees are of density g individuals per square meter, there will be on average $g\pi r^2$ individuals within the neighborhood defined by r. Therefore, $E_F = g\pi r^2$.

The calculation of $\mathbb{E}[S_{i,k}(x)]$ is as follows. Consider a single patch occupied by species k. It is necessary to calculate the probability an adult of species i is within E_F. Recall p_i be the proportion of species i in the population. Assuming species are approximately randomly distributed, the probability that a conspecific is within the radius r of a random patch can be captured with a 2D Poisson process (i.e. a spatial Poisson process) with a rate parameter of $\lambda = p_i g$. The waiting time of first event of a 2D Poisson process is exponentially distributed such that

$$\mathrm{P}[i \notin E_F] = e^{-\lambda \mathrm{A}} \tag{A.7}$$

where A is the area of the circle of radius r stemming from the focal patch. Therefore $\mathrm{A} = \pi r^2$

and $\lambda A = p_i g \pi r^2$ such that

$$\begin{aligned} \text{P}[i \notin E_F] &= e^{-p_i g \pi r^2} \\ \text{P}[i \in E_F] &= 1 - e^{-p_i g \pi r^2} \end{aligned} \tag{A.8}$$

Additionally, as defined by equation (A.3), $S_{i,k}^{i \notin E_F}(x) = Y_i p_i D$ and $S_{i,k}^{i \in E_F}(x) = Y_i p_i D e^{-a}$. Letting $g \pi r^2 = E_F$ and using the above probabilities, the expected offspring value of $S_{i,k}(x)$ is:

$$\begin{aligned} \mathbb{E}[S_{i,k}(x)] &= \text{P}[i \notin E_F] \times S_{i,k}^{i \notin E_F}(x) + \text{P}[i \in E_F] \times S_{i,k}^{i \in E_F}(x) \\ &= Y_i p_i D \big[e^{-p_i E_F} + e^{-a}(1 - e^{-p_i E_F}) \big] \end{aligned} \tag{A.9}$$

Plugging this value into the offspring abundance terms yields

$$\begin{aligned} \mathbb{E}[S_{i,i}(x)] &= Y_i \big[(1 - D) + p_i D \big] e^{-a} \\ \mathbb{E}[S_{i,k}(x)] &= Y_i p_i D \big[e^{-p_i E_F} + e^{-a}(1 - e^{-p_i E_F}) \big] \end{aligned} \tag{A.10}$$

noting that $S_{all,i}(x) = \sum_{n=1}^{N} S_{n,i}(x)$. These equations are identical to the offspring abundance equations for the NF model in the main text.

3 Comparison between ODE model and SEM

In this section, I describe simulations that compare the ODE model to the SEM. Results show that the SEM and ODE models yield highly similar outputs of species abundance and species richness.

3.1 ODE and SEM parameterization

Each SEM simulation began with 300 species at equal abundance, with individuals randomly distributed throughout the community. Simulations were conducted on a 275×275 torus (thus containing 275^2 individual trees). I use the following parameters: $Y \sim \text{lognormal}[\mu = 0, \sigma_Y]$

with $\sigma_Y \sim \{0.1, 0.45, 0.8\}$ and $a \sim \{0.5, 1.0, 2.75, 4.5\}$. In all simulations, $D = 1$. I tested each of the 12 parameter combinations of σ_Y and a with $E_F = 9$, 25, 49, 81, and 121. This corresponds to examining a range of r approximately between 4 and 14 (assuming $g = 0.2$). This generated 60 outputs. Simulations were run for about 75 generations, sufficient time for the community to approximately reach equilibrium without drift dominating the dynamics of the lower abundance species. However, it was noticed that the cases in which $\sigma_Y = 0.1$ had longer transient dynamics. These were run for 150 generation instead. See Fig. A6-A8 for examples of the transient dynamics.

I then ran a set of ODE simulations using the same parameterizations as the SEM. I compared the outputs of the SEM and ODE model in terms of species diversity, species abundance, and Shannon diversity. I considered a species to be extinct if it had less than 1 individual at any point of the simulation. This was implemented directly in the SEM; for the ODE model, I assumed a species, i, to be extinct if $p_i^* < 1/275^2$ where p_i^* is the equilibrium proportion of species i. Note that these simulations do not attempt to demonstrate the long-term resistance against extinction due to drift. Rather, they demonstrate that the ODE model and SEM yield similar outputs of expected species abundance and richness given the same parameterization.

3.2 Results of comparison

The ODE model and SEM yielded very similar results in species richness and Shannon diversity (Figs. A1, A2). The ODE model and SEM also produced very similar species proportions (Figs. A3-A5). To quantify the quality of the approximation, I calculated the mean difference in species richness between the ODE model and SEM, ΔR:

$$\Delta R = \frac{1}{S}\sum_{k=1}^{S}(R^k_{\text{SEM}} - R^k_{\text{ODE}}) \tag{A.11}$$

where S is the number of simulations, and R^k_{SEM} and R^k_{ODE} are the species diversity of the k_{th} simulation of the SEM and ODE model, respectively. I also examined the r^2 (coefficient of determination) between SEM and ODE diversity. For the comparisons, $\Delta R = 1.6$ and $r^2 = 0.987$. Overall, the ODE provides a highly similar, albeit non-exact, estimation of species diversity. Cases in which the ODE model predicted lower species richness are likely due to incomplete transient dynamics of the SEM.

4 Derivation of invasion criteria

In this section, I derive the approximate invasion criteria of the fixed neighborhood effect model when species experience inter-specific variation in intrinsic (Y) and $D = 1$. The invasion criteria of an invader can be expressed as when the per capita growth rate as $p_i \to 0$. Letting

$$\mathbb{E}[J_{k,i}(x)] = e^{-p_k E_F} + e^{-a}(1 - e^{-p_k E_F}) \tag{A.12}$$

The per capita growth rate of species i, substituting in the offspring abundance values, is

$$\begin{aligned}\frac{1}{p_i}\frac{dp_i}{dt} = r_i = \delta\Bigg[&\frac{Y_i\big[(1-D) + p_i D\big]e^{-a}}{Y_i\big[(1-D) + p_i D\big]e^{-a} + \sum_{k\neq i} Y_k p_k D\,\mathbb{E}[J_{k,i}(x)]} \\ &+ Y_i D \sum_{m\neq i} \frac{1}{Y_m e^{-a}\big[(1-D) + p_m D\big] + \sum_{k\neq m} Y_k p_k D\,\mathbb{E}[J_{k,m}(x)]} p_m - 1\Bigg]\end{aligned} \tag{A.13}$$

Species i can invade is this quantity if positive when it is rare ($p_i \to 0$). When $D = 1$ (the case of interest), the above reduces to

$$Y_i \sum_{m\neq i} \frac{p_m}{Y_m e^{-a} p_m + \sum_{k\neq m} Y_k p_k\,\mathbb{E}[J_{k,m}(x)]} > 1 \tag{A.14}$$

To simplify the above equation, I ignore the term $Y_m e^{-a} p_m$ in the denominator and incorporate an additional term the summation, yielding:

$$Y_i \sum_{m \neq i} \frac{p_m}{\sum_{k \neq i} Y_k p_k \, \mathbb{E}[J_{k,i}(x)]} > 1 \tag{A.15}$$

This simplification is equivalent to making the species identity of the tree previously occupying a patch (the tree that dies) irrelevant (i.e., JCEs only result from trees nearby the patch rather than the previous occupant of the patch). As long as neighborhood effects are somewhat strong – that is, so long as E_F is not very small – this assumption should not meaningfully affect the invasion criteria.

Importantly, the denominator of equation (A.15) is no longer directly dependent on m. That is, equation (A.15) can be rewritten as

$$Y_i \Bigg(\sum_{m \neq i} p_m \Bigg) \Bigg(\frac{1}{\sum_{k \neq i} Y_k p_k \, \mathbb{E}[J_{k,i}(x)]} \Bigg) > 1 \tag{A.16}$$

Because, by definition, $\sum_{m \neq i} p_m = 1$, the above equation can be rewritten as

$$Y_i > \sum_{k \neq i} Y_k p_k \, \mathbb{E}[J_{k,i}(x)] \tag{A.17}$$

Substituting the appropriate value for $\mathbb{E}[J_{k,i}(x)]$, the invasion criteria becomes

$$Y_i > \sum_{k \neq i} Y_k p_k \big(1 - (1 - e^{-a})(1 - e^{-p_k E_F}) \big) \tag{A.18}$$

noting that the above equation has been slightly rearranged, but is the same as equation (A.12). I then take the linearization of $p_k \Bigg(1 - (1 - e^{-a})(1 - e^{-p_k E_F}) \Bigg)$ around the point $1/N$ where N is the number of species in the resident community yields a close approximation of the expression so long

as variation in p_k does not span values much greater than $1/N$ (mean community abundance). Then,

$$
\begin{aligned}
p_k\left(1-(1-e^{-a})(1-e^{-p_k E_F})\right) \approx & \frac{1}{N}\left(1-(1-e^{-a})\big(1-e^{-\frac{E_F}{N}}\big)\right)+ \\
& \left(e^{-\frac{E_F}{N}}\left(1-e^{-a}\right)\left(1-\frac{E_F}{N}\right)+e^{-a}\right)\left(p_k-\frac{1}{N}\right)
\end{aligned}
\tag{A.19}
$$

Substituting this into the equation, the summation (the right hand side of the invasion criteria) can be rearranged and broken into two into three summations:

$$
\begin{aligned}
\frac{1}{N}\left(1-(1-e^{-a})\big(1-e^{-\frac{E_F}{N}}\big)\right)\sum_{k\neq i} Y_k + \left(e^{-\frac{E_F}{N}}\left(1-e^{-a}\right)\left(1-\frac{E_F}{N}\right)+e^{-a}\right)\sum_{k\neq i} Y_k p_k - \\
\left(e^{-\frac{E_F}{N}}\left(1-e^{-a}\right)\left(1-\frac{E_F}{N}\right)+e^{-a}\right)\frac{1}{N}\sum_{k\neq i} Y_k
\end{aligned}
\tag{A.20}
$$

The first and third summations are trivial to evaluate:

$$
\frac{1}{N}\left(1-(1-e^{-a})\big(1-e^{-\frac{E_F}{N}}\big)\right)\sum_{k\neq i} Y_k = \left(1-(1-e^{-a})\big(1-e^{-\frac{E_F}{N}}\big)\right)\overline{Y} \tag{A.21}
$$

and

$$
\left(e^{-\frac{E_F}{N}}\left(1-e^{-a}\right)\left(1-\frac{E_F}{N}\right)+e^{-a}\right)\frac{1}{N}\sum_{k\neq i} Y_k = \left(e^{-\frac{E_F}{N}}\left(1-e^{-a}\right)\left(1-\frac{E_F}{N}\right)+e^{-a}\right)\overline{Y} \tag{A.22}
$$

where $\overline{Y}$ is the mean fitness of the resident community.

The second summation can be evaluated by using the property

$$
\frac{1}{N}\sum_{m=1}^{N} A_m B_m = \overline{A}\times\overline{B} + \mathrm{Cov}(A,B) \tag{A.23}
$$

I apply this property with respect to p and Y, noting that $\overline{p} = \frac{1}{N}$. Using this property and

substituting A and B with p and Y. Doing this yields:

$$\left(e^{-\frac{E_F}{N}}\left(1-e^{-a}\right)\left(1-\frac{E_F}{N}\right)+e^{-a}\right)\sum_{k\neq i} Y_k p_k = \left(e^{-\frac{E_F}{N}}\left(1-e^{-a}\right)\left(1-\frac{E_F}{N}\right)+e^{-a}\right)\left[\overline{Y}+N\,\mathrm{Cov}(p,Y)\right] \tag{A.24}$$

noting that $\overline{p}=1/N$.

Adding all three sums together, equation (A.22) and the $\overline{Y}$ term from equation (A.24) will cancel. Then, after some rearraning, the invasion criteria becomes

$$Y_i > \underbrace{\overline{Y}\left(1-\left(1-e^{-a}\right)\left(1-e^{\frac{-E_F}{N}}\right)\right)}_{\substack{\text{mean} \\ \text{JCE-fitness term}}} + \underbrace{N\mathrm{Cov}(p,Y)\left(e^{-\frac{E_F}{N}}\left(1-e^{-a}\right)\left(1-\frac{E_F}{N}\right)+e^{-a}\right)}_{\text{covariance-JCE term}} \tag{A.25}$$

which is the same as the corresponding equation in Table 1 of the main text.

5 Note on ODE approximation

To derive the ODE model, I took approximations of the expected values of $P_{i,i}(x)$ and $P_{i,k}(x)$. To do so, I took the expected abundance of $S_{i,i}(x)$, $S_{i,k}(x)$, and $S_{all,i}(x)$ with respect to space and then examined their quotients. Note that this assumes $\mathbb{E}[S_{i,k}(x)/S_{all,i}(x)] \approx \mathbb{E}[S_{i,k}(x)]/\,\mathbb{E}[S_{all,k}(x)]$ (I take the expectation of the numerator and denominator and then take the quotient). Using a Taylor Expansion about the mean,

$$\mathbb{E}\left[\frac{S_{i,k}(x)}{S_{all,i}(x)}\right] \approx \frac{\mathbb{E}[S_{i,k}(x)]}{\mathbb{E}[S_{all,k}(x)]} - \frac{\mathrm{Cov}\big(S_{i,k}(x), S_{all,k}(x)\big)}{\mathbb{E}[S_{all,k}(x)]^2} + \mathrm{Var}\big(S_{all,k}(x)\big)\frac{\mathbb{E}[S_{i,k}(x)]}{\mathbb{E}[S_{all,i}(x)]^3}$$

Because there are many species in the community, $S_{all,i}(x) >> S_{i,k}(x)$. This implies that the covariance term and the term containing $\mathbb{E}[S_{all,i}(x)]^3$ are close to zero. Additionally, $\mathrm{Var}\big(S_{all,i}(x)\big)$ is likely small because it is assumed that dispersal is uniform across the community. Therefore, $\mathbb{E}[S_{i,k}(x)/S_{all,i}(x)] \approx \mathbb{E}[S_{i,k}(x)]/\,\mathbb{E}[S_{all,k}(x)]$ is likely a good approximation. I rely on the

quantitative similarity of the SEM and ODE model to validate this assumption.

References

Chisholm, R.A. & Fung, T. (2020). Janzen-connell effects are a weak impediment to competitive exclusion. *Am. Nat.*, 196, 649–661.

Levi, T., Barfield, M., Barrantes, S., Sullivan, C., Holt, R.D. & Terborgh, J. (2019). Tropical forests can maintain hyperdiversity because of enemies. *PNAS*, 116, 581–586.

6 Figures

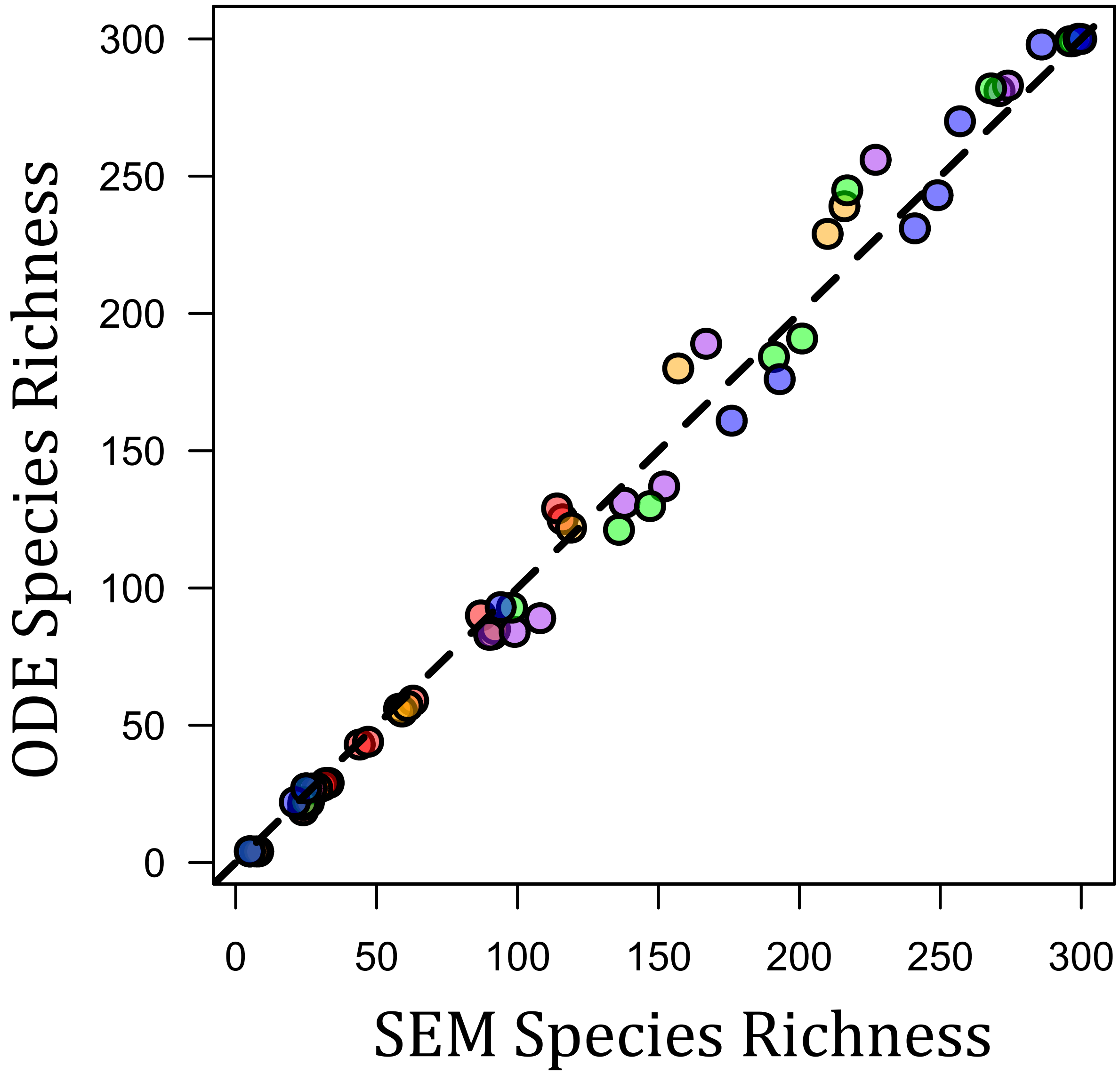

Fig. A1: ODE model validation. The figures compare species richness between SEM and ODE model simulations under identical parameterizations. The dashed line is the one-to-one line (points on the line represent when the SEM and ODE yield the exact same diversity output). Red points are when $E_F = 9$, orange/yellow points are when $E_F = 25$, purple points are when $E_F = 49$, green points are when $E_F = 81$, and blue points are when $E_F = 121$. To a first approximation, the ODE model yields the same output as the SEM. These parameter values span the most of the parameter space explored in Figs. 2 and 3 of the main text.

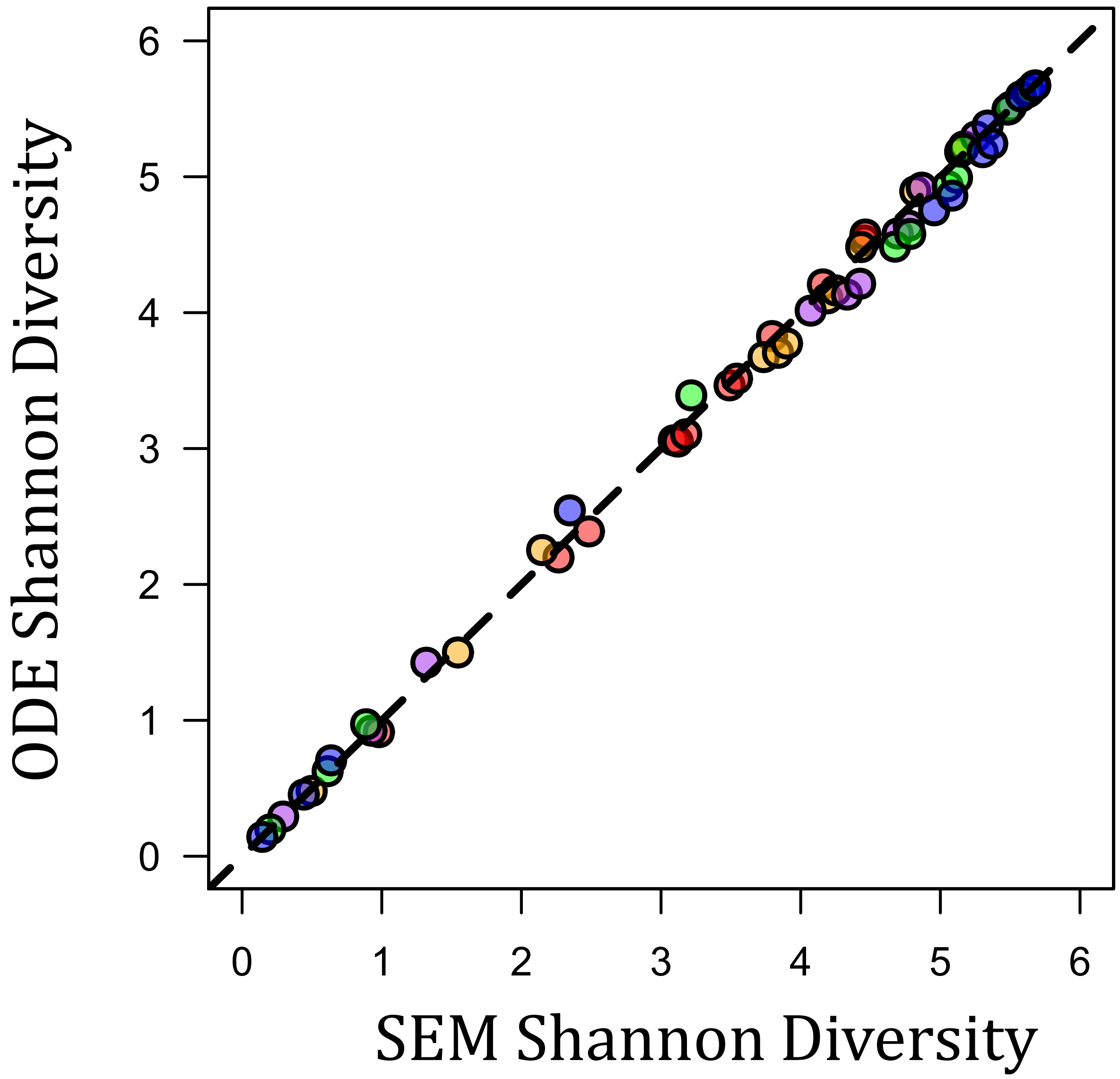

Fig. A2: The same as the Fig. A1, but showing Shannon Diversity instead of species richness. As in the above case, the SEM and ODE model yield very similar outputs.

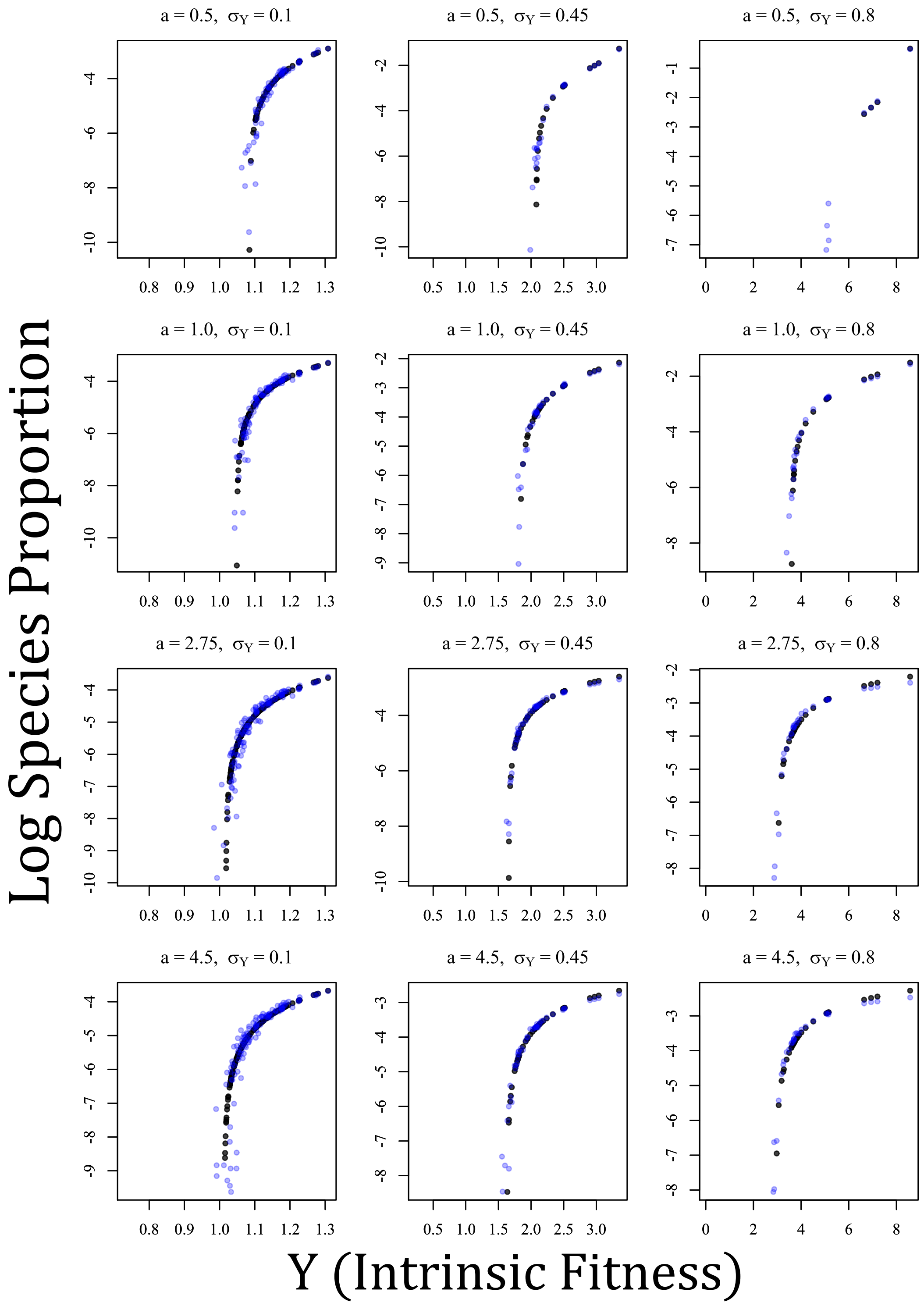

Fig. A3: Comparisons between identical parameterizations of the ODE approximation (black) and SEM (blue) outputs under twelve parameter values when species vary in intrinsic fitness (Y). The y-axis depicts the log-proportion of each species and the x-axis depicts Y of each species. In all plots, $E_F = 9$, and $D = 1.0$. Other relevant parameters are listed on each plot.

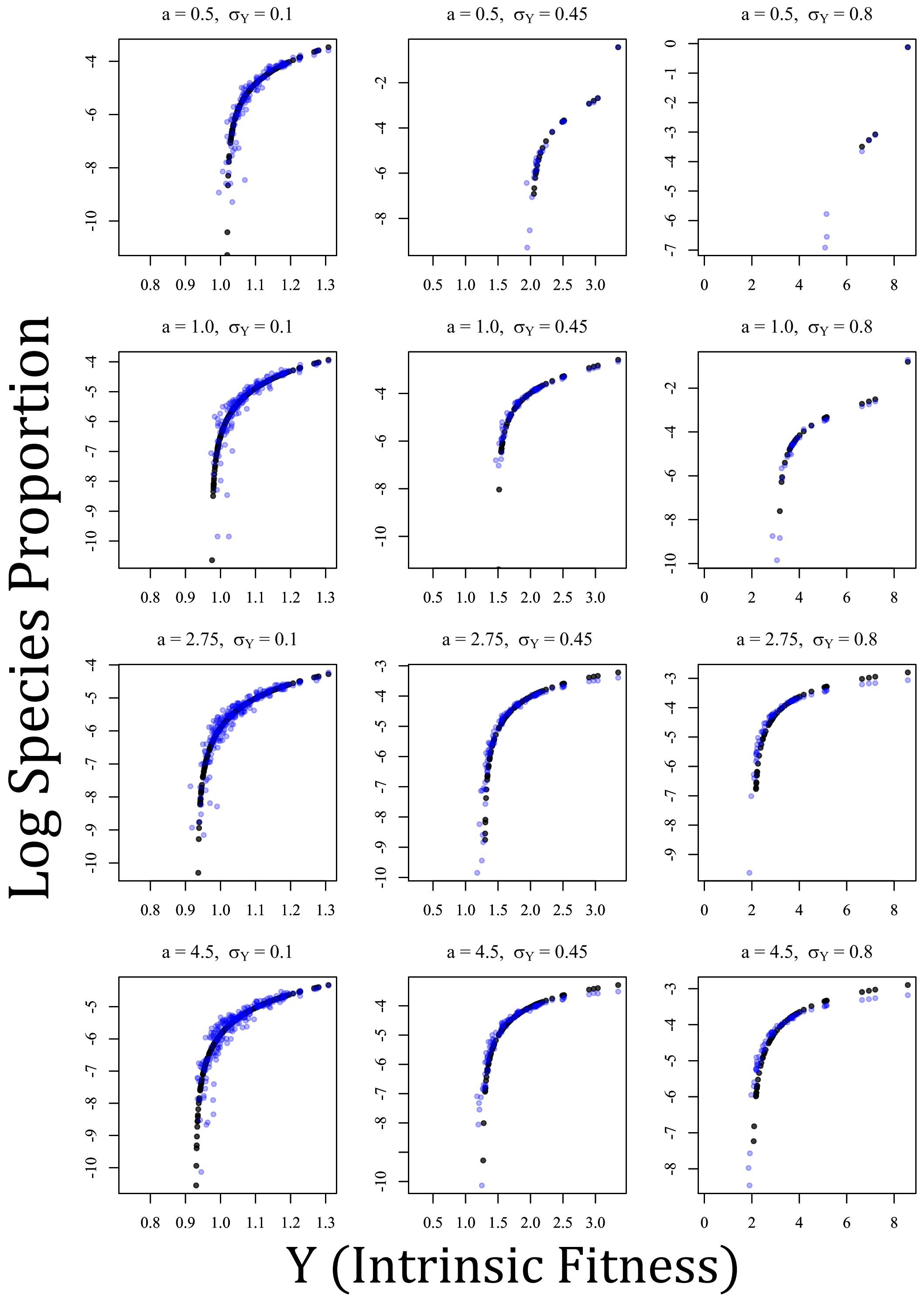

Fig. A4: The same format as Fig. A3, but with $E_F = 25$.

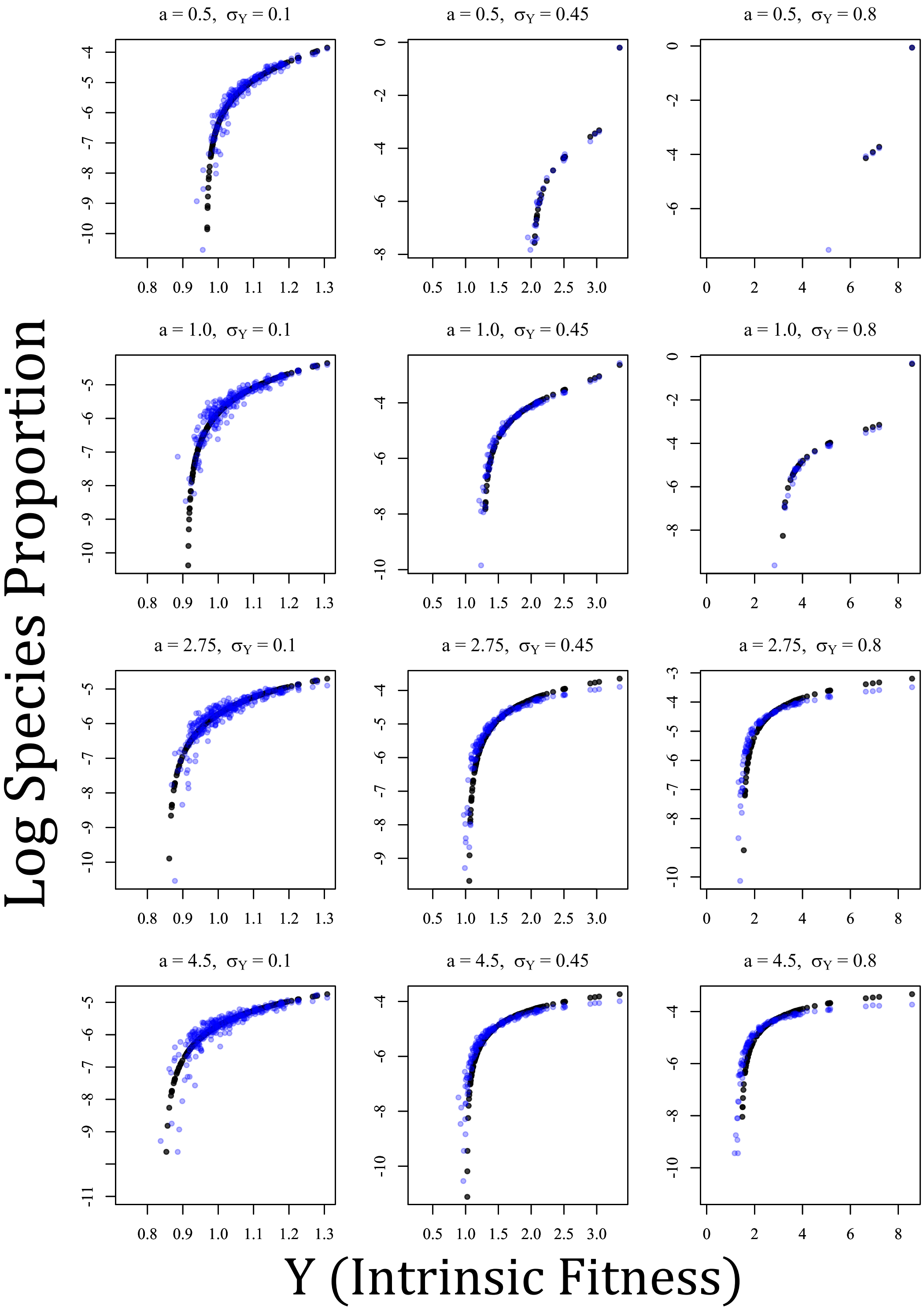

Fig. A5: The same format as Fig. A3, but with $E_F = 49$.

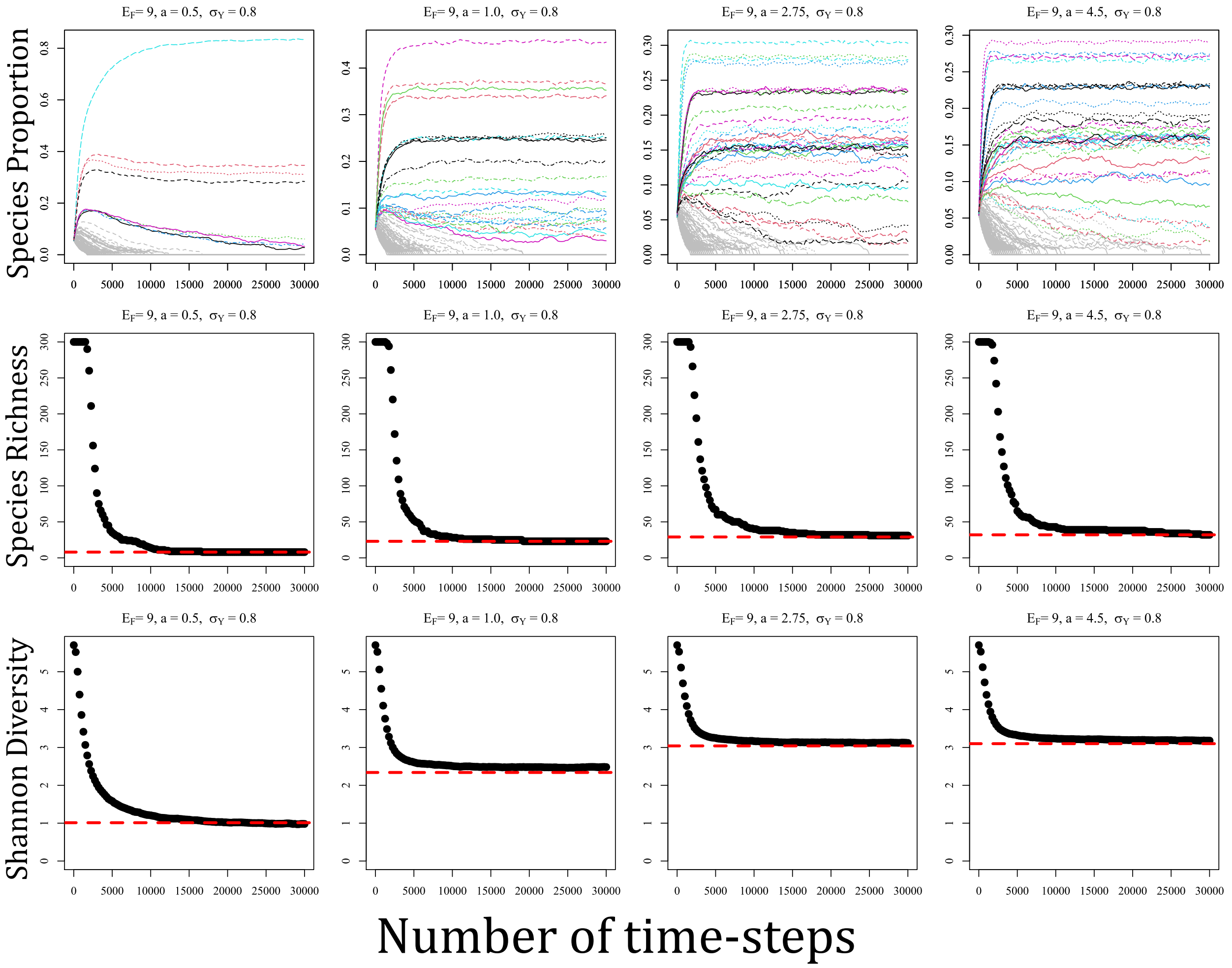

Fig. A6: Examples of the SEM simulation time series outputs, species richness over time in the simulations, and Shannon diversity over time in the simulations. The top row shows examples of the time series outputs of the SEMs. The x-axis is time and the y-axis is each species' proportion. Proportions have been square-root transformed to aid visualization. Colored trajectories indicate species that persisted throughout the simulation; grey trajectories indicate species that went extinct. Parameters are listed on each plot. Dynamics as shown are typical examples from the SEMs. Most species settle into a relatively stable pseudo-equilibrium, while lower abundance species fluctuate due to drift. The second row shows the number of persisting species in the community as a function of time. Each panel corresponds to the plot above it. Most species that go extinct do so in the early stages of the dynamics. Therefore, the vast majority of persisting species likely persist deterministically. The dashed red line is the diversity maintained by the ODE under the same parameterization. All SEMs saturate, approximately, to the dashed line. The third row is the same as the second row, except it shows Shannon diversity instead of species richness.

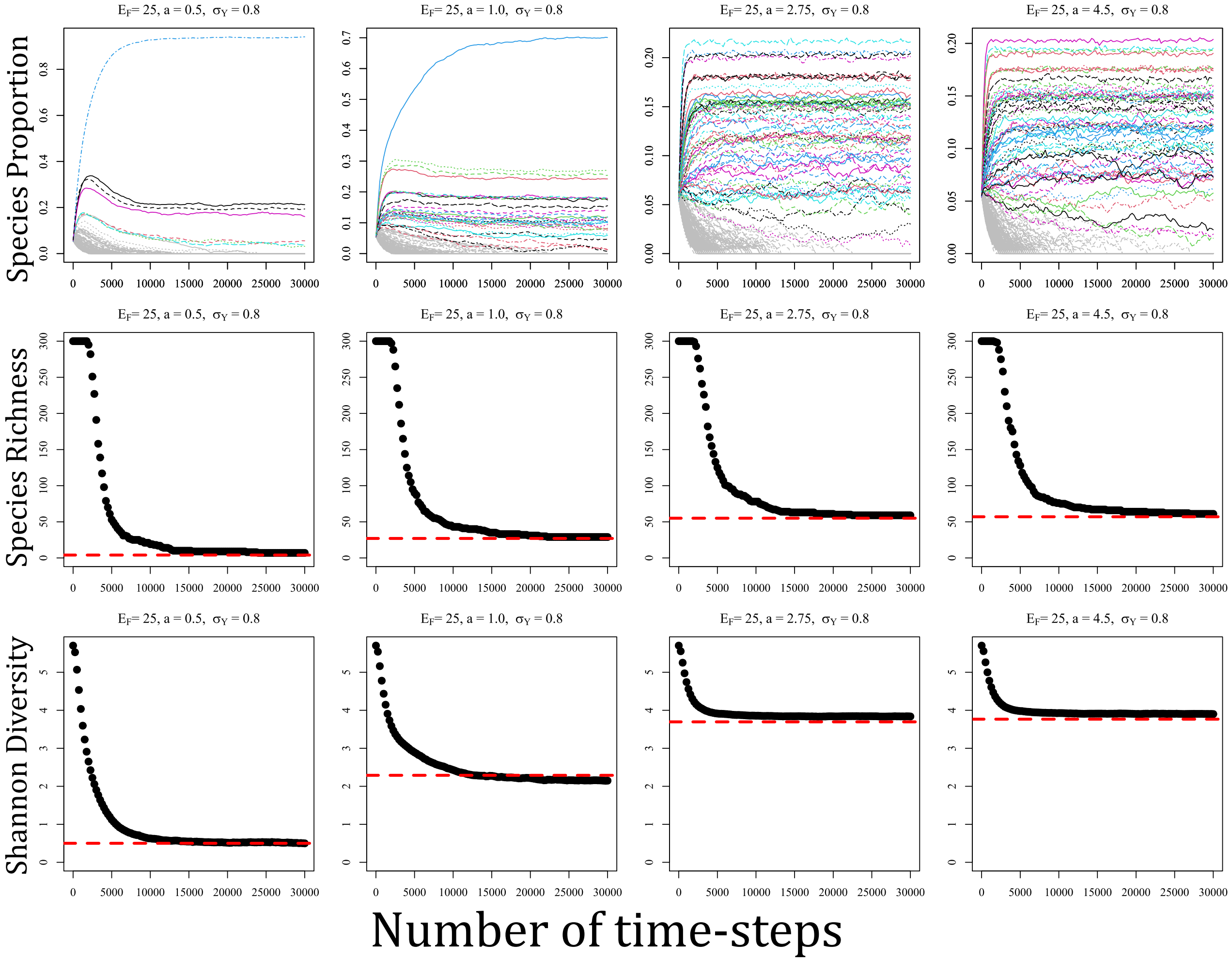

Fig. A7: The same format as Fig. A6, but with $E_F = 25$.

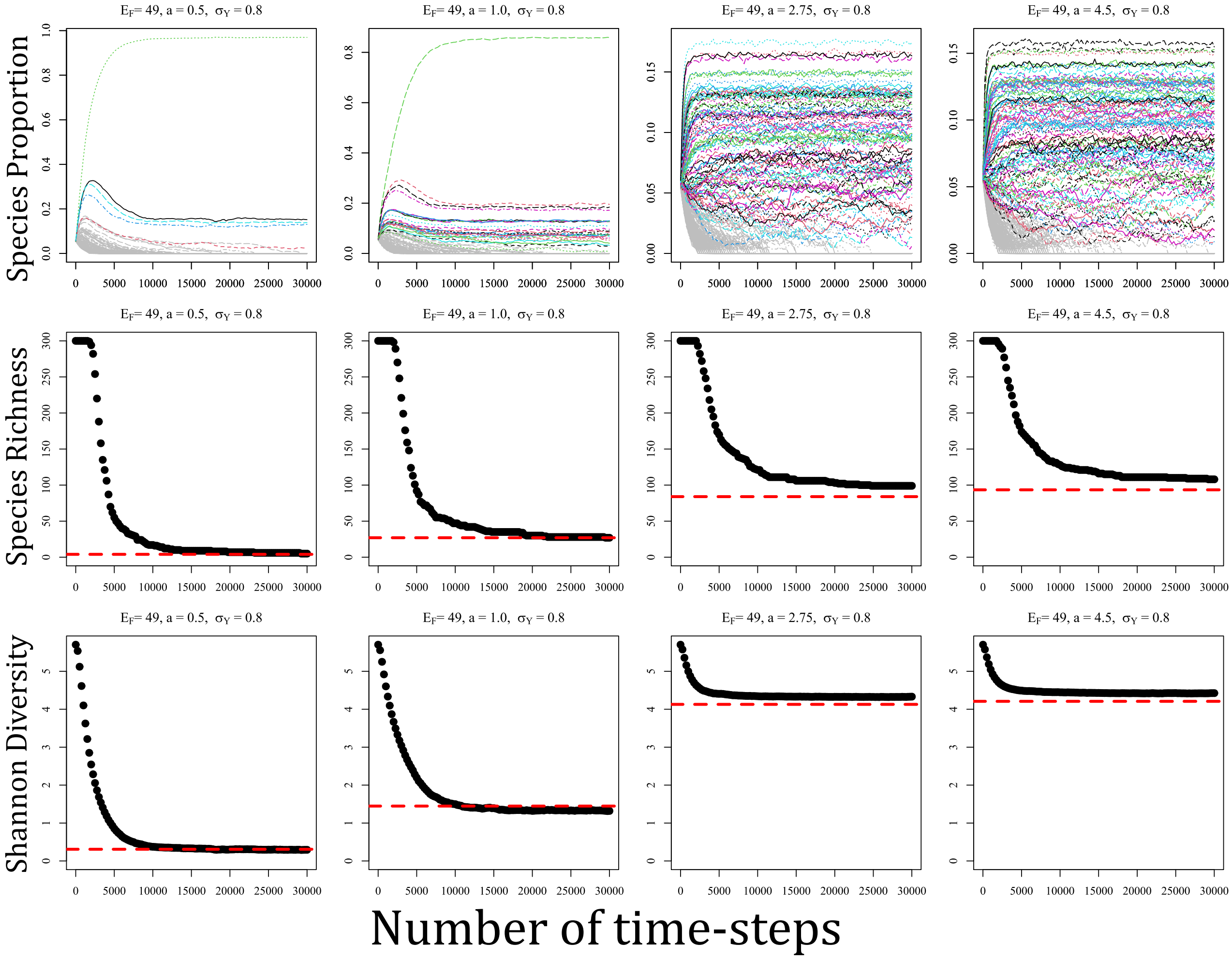

Fig. A8: The same format as Fig. A6, but with $E_F = 49$.
