# Supplementary material for: The functional form of specialised predation affects whether Janzen–Connell effects can prevent competitive exclusion
Source: Ecol Lett. 2022 Apr 26;25(6):1458–70. doi: 10.1111/ele.14014 (PMC9324109; doi:10.1111/ele.14014)
Supplement: Supplementary file 2 — Supplementary Material [file ELE-25-1458-s004.pdf]

Appendix B: Additive−Fixed-distance model SEM and ODE approximation

Contents

1 Introduction **2**

2 SEM and derivation of ODE approximation **2**

2.1 SEM 2

2.2 ODE Model 4

3 Comparison between ODE model and SEM **7**

3.1 ODE and SEM parameterization 7

3.2 Results of comparison 8

4 Derivation of invasion criteria **8**

5 Note on ODE approximation **12**

6 Figures **12**

1 Introduction

In this Appendix, I analyze the Additive−Fixed-distance (AF) model presented in the main text. This Appendix is composed of three main sections: **(1)** I introduce a spatially explicit model (SEM) of the AF model. I then demonstrate that taking the expected offspring abundances on each patch yields the ODE model discussed in the main text. **(2)** I provide outputs of the ODE model and the SEM model under the same parameterizations. I show the outputs are very similar, hence demonstrating that the ODE is a sufficiently accurate approximation of the SEM. **(3)** I provide the derivation of the invasion criteria approximation of the AF model.

2 SEM and derivation of ODE approximation

In this section, I discuss the SEM, briefly reviewing within-patch dynamics from the main text. Then, I show the derivation of the ODE approximation. I assume the reader is generally familiar with the model discussed in the main text.

2.1 SEM

I developed a spatially explicit model (SEM) that integrates the AF model. The model consists of a community on a grid of $L \times L$ patches (M total patches, $M = L^2$) modeled as a torus to avoid edge effects. A single tree is present on every space on the grid. At each time step, each tree dies with probability δ and tree replacements occur via a lottery model based on the relative abundance of offspring of each species on each patch. For the AF model, predation pressure increases linearly as a function of local conspecific density and occurs within a fixed area surrounding each tree (as decried in the main text). As noted in the main text, offspring

abundances are defined by

$$
\begin{aligned}
S_{i,i}(x) &= Y_i\big[(1-D)+p_i d\big]J_{i,i}(x)\\
S_{i,k}(x) &= Y_i p_i D J_{i,k}(x)\\
S_{all,i}(x) &= \sum_{n=1}^{N} S_{n,i}(x)
\end{aligned}
\tag{B.1}
$$

where $S_{A,B}(x)$ is the offspring abundance of species A on a patch occupied by species B at location x, $J_{i,i}(x)$ and $J_{i,k}(x)$ are how JCEs affect offspring surivorship, p_i is the proportion of species i in the population, Y_i is the intrinsic fitness of species i, and D is the dispersal proportion. For AF model,

$$
\begin{aligned}
J_{i,i}(x) &= \exp\Bigg[-a\bigg(1+\sum_{m\in r}\mathbb{1}_m(i)\bigg)\Bigg]\\
J_{i,k}(x) &= \exp\Bigg[-a\sum_{m\in r}\mathbb{1}_m(i)\Bigg]
\end{aligned}
\tag{B.2}
$$

where $\mathbb{1}_k(i)$ is an indicator function for which

$$
\mathbb{1}_m(i) = \begin{cases} 1, & \text{if } m = i \\ 0, & \text{if } m \neq i \end{cases}
\tag{B.3}
$$

in which case, the predation pressure is equal to the sum of individuals found within the effect area defined by r.

Offspring abundances are then determined by the following equations:

$$S_{i,i}(x) = Y_i\big[(1-D)+p_iD\big]\exp\Bigg[-a\bigg(1+\sum_{m\in r}\mathbb{1}_m(i)\bigg)\Bigg]$$
$$S_{i,k}(x) = Y_ip_iD\ \exp\Bigg[-a\sum_{m\in r}\mathbb{1}_m(i)\Bigg]$$
$$S_{all,i}(x) = Y_i\big[(1-D)+Dp_i\big]\exp\Bigg[-a\bigg(1+\sum_{m\in r}\mathbb{1}_m(i)\bigg)\Bigg]+\sum_{k\neq i}Y_kp_kD\ \exp\Bigg[-a\sum_{m\in r}\mathbb{1}_m(k)\Bigg] \tag{B.4}$$

For the discrete grid cells, it makes more sense to think about the number of grid-cells that fall within r rather than r itself. Therefore, let E_F represent the number of patches that wall within an $n\times n$ Moore neighborhood around a focal tree (as would be contained within r). Thus, $E_F = \pi r^2 g$, where g is tree density in individuals per meters squared. Notably, for the SEM, values of r must be selected such that they correspond to viable Moore neighborhood values (such that $E_F = 9$, 25, 49, etc.). Technically, this is not exactly the same as the model described above (Moore neighborhoods are square rather than circular) but is essentially mathematically identical.

The lottery is determined by the relative seedling abundances. Let $P_{A,B}(x)$ be the probability species A colonizes a patch previously occupied by species B at location x. Then, $P_{i,i}(x) = S_{i,i}(x)/S_{all,i}(x)$ and $P_{i,k} = S_{i,k}(x)/S_{all,k}(x)$.

2.2 ODE Model

To derive the ODE model, I take approximations of the expected values of $P_{i,i}(x)$ and $P_{i,k}(x)$. To do so, I take the expected abundance of $S_{i,i}(x)$, $S_{i,k}(x)$, and $S_{all,i}(x)$ and then take their quotients.

Expectations are taken with respect to space. Using this, I derive the the ODE approximation

$$\frac{dp_i}{dt} = \delta\left[\frac{\mathbb{E}[S_{i,i}(x)]}{\mathbb{E}[S_{all,i}(x)]}p_i + \sum_{k\neq i}\frac{\mathbb{E}[S_{i,k}]}{\mathbb{E}[S_{all,k}(x)]}p_k - p_i\right] \tag{B.5}$$

that captures the behavior of the SEM. See "Note" at the end of this Appendix for additional information about the assumptions of this approximation.

The additive predation function in the SEM is implemented spatially explicitly – the location of trees are, computationally, is stored in a matrix. The deterministic ODE model is implicit. Therefore, it is necessary to use an approximation of the terms that does not require spatial information. In particular, the quantity $S_{i,k}(x)/S_{all,k,x}$, which contains the term

$$\mathbb{E}[S_{i,k}(x)] = \exp\left[-a\sum_{m\in r}\mathbb{1}_m(i)\right] \tag{B.6}$$

This quantity must be approximated as a spatially implicit term.

Consider a single patch occupied by species k. It is necessary to calculate the expected predation experienced by species i given that the JCE effect radius is r. Recall that g is the density of trees in square meters, r is the distance in meters from the local patch that defines the effect area, and p_i is the proportion of species i in the population. Assuming trees are approximately randomly distributed, the number of trees of species i within r meters of the focal patch can be described by with a Poisson distribution with a rate parameter of $\lambda = \pi g r^2 p_i$. Then, letting X_i be the number of individuals of species i within r, the probability that there are m individuals of species i within radius r is

$$\mathrm{P}[X_i = m] = \frac{(\pi g r^2 p_i)^m}{m!}e^{-\pi g r^2 p_i}$$

Now, recalling that $E_F = \pi r^2 g$ and that offspring survival decreases exponentially with the

number of conspecific adults falling within r, the expectation can be calculated as follows:

$$\begin{aligned}\mathbb{E}[S_{i,k}(x)] &= \mathbb{E}\left[\exp\left[-a\sum_{m=1}^{Mp_i}\mathbb{1}_m(i)\right]\right] \\ &= \sum_{m=0}^{\infty}\mathrm{P}(X_i = m)\times[\text{probability of survival given } X_i = m] \\ &= \sum_{m=0}^{\infty}\frac{(E_F p_i)^m}{m!}e^{-E_F p_i}e^{-a\,m} \\ &= e^{-E_F p_i}\sum_{m=0}^{\infty}\frac{(E_F p_i e^{-a})^m}{m!} \\ &= e^{-(1-e^{-a})p_i E_F}\end{aligned} \tag{B.7}$$

Similarly,

$$\begin{aligned}\mathbb{E}[S_{i,i}(x)] &= \mathbb{E}\left[\exp\left[-a\left(1+\sum_{m=1}^{Mp_i}\mathbb{1}_{x_m^i}(r)\right)\right]\right] \\ &= e^{-a}\sum_{m=0}^{\infty}\mathrm{P}(X_i = m)\times[\text{probability of survival given } X_i = m] \\ &= e^{-a}\sum_{m=0}^{\infty}\mathrm{P}(X_i = m)e^{-am} \\ &= e^{-a}e^{-E_F p_i}\sum_{m=0}^{\infty}\frac{(E_F p_i e^{-a\,m})^m}{m!} \\ &= e^{-a}e^{-(1-e^{-a})p_i E_F}\end{aligned} \tag{B.8}$$

Plugging these values value into the appropriated post-JCE offspring abundance terms yields

$$\begin{aligned}\mathbb{E}[S_{i,i}(x)] &= Y_i\big[(1-d)+dp_i\big]e^{-a}e^{-(1-e^{-a})p_i E_F} \\ \mathbb{E}[S_{i,k}(x)] &= Y_i dp_i e^{-(1-e^{-a})p_i E_F}\end{aligned} \tag{B.9}$$

also noting that $S_{all,i}(x) = \sum_{n=1}^{N} S_{n,i}(x)$. This is identical to the relevant expression for the AF model in the main text.

3 Comparison between ODE model and SEM

In this section, I describe simulations that compare the ODE model to the SEM. I demonstrate that the SEM and ODE model highly similar species abundance and species richness outputs.

3.1 ODE and SEM parameterization

Each SEM simulation began with 300 species at equal abundance, with individuals randomly distributed throughout the community. Simulations were conducted on a 275×275 torus (thus containing 275^2 individual trees). I use the following parameters: $Y \sim \text{lognormal}[\mu = 0, \sigma_Y]$ with $\sigma_Y \sim \{0.1, 0.45, 0.8\}$ and $a \sim \{0.5, 1.0, 2.75, 4.5\}$. In all simulations, $D = 1$. I tested each of the 12 parameter combinations with $E_F = 9$, 25, 49, 81, and 121. This corresponds to examining a range of r between 3.8 and 14 (assuming $g = 0.2$). This generated 60 outputs. Simulations were run for about 75 generations, sufficient time for the community to approximately reach equilibrium without drift dominating the dynamics of the lower abundance species. However, it was noticed that the cases in which $\sigma_Y = 0.1$ had longer transient times. These were run for 150 generation instead. See Fig. B6-B8 for examples of the transient dynamics.

I then ran a set of ODE simulations using the same parameterizations as the SEM. I compared the outputs of the SEM and ODE model in terms of species diversity, species abundance, and Shannon diversity. I considered a species to be extinct if it had less than 1 individual at any point of the simulation. This was implemented directly in the SEM; for the ODE model, I assumed a species, i, to be extinct if $p_i^* < 1/275^2$ where p_i^* is the equilibrium proportion of species i. Note that these simulations do not attempt to demonstrate the long-term resistance against extinction due to drift. Rather, they demonstrate that the ODE model and SEM yield similar outputs of expected species abundance and species richness given the same parameterization.

3.2 Results of comparison

ODE model and SEM produced very similar species richness and Shannon diversity (Figs. B1, B2). The ODE model and SEM also produced very similar species proportions (Figs. B3-B5). To quantify the quality of the approximation, I calculated the mean difference in species richness between the ODE model and SEM, ΔR:

$$\Delta R = \frac{1}{S}\sum_{k=1}^{S}(R^k_{\text{SEM}} - R^k_{\text{ODE}}) \tag{B.10}$$

where S is the number of simulations, and R^k_{SEM} and R^k_{ODE} are the species richness of the k_{th} simulation of the SEM and ODE model, respectively. I also examined the r^2 (coefficient of determination) between SEM and ODE richness. For the comparisons, $\Delta R = 2.1$ and $r^2 = 0.985$. Overall, the ODE provides a highly similar, albeit non-exact, estimation of species diversity. Error in which the ODE model predicted greater diversity than the SEM is most likely due to stochastic extinction due to drift. This is particularly likely when diversity is high, where the expected abundance of each species is correspondingly smaller. Cases in which the ODE model predicted lower species richness are likely due to incomplete transient dynamics of the SEM.

4 Derivation of invasion criteria

In this section, I derive the approximate invasion criteria of the additive−fixed-distance model when species experience inter-specific variation in intrinsic (Y) and $D = 1$. The invasion criteria of an invader can be expressed as when the per capita growth rate as $p_i \to 0$. Recalling that

$$\mathbb{E}[J_{k,i}(x)] = e^{-(1-e^{-a})p_i E_F} \tag{B.11}$$

and

$$\mathbb{E}[J_{i,i}(x)] = e^{-a} e^{-(1-e^{-a})p_i E_F} \tag{B.12}$$

and using variables previous defined in this appendix, the per capita growth rate of species i (substituting in the seedling abundance values) is

$$\begin{aligned}\frac{1}{p_i}\frac{dp_i}{dt} = r_i = \delta\Bigg[&\frac{Y_i\big[(1-D)+p_iD\big]}{Y_i\big[(1-D)+p_iD\big]\,\mathbb{E}[J_{i,i}(x)] + \sum_{k\neq i} Y_k p_k D\,\mathbb{E}[J_{k,i}(x)]}\\ &+ Y_i D \sum_{m\neq i} \frac{1}{Y_m\big[(1-D)+p_mD\big]\,\mathbb{E}[J_{m,m}(x)] + \sum_{k\neq m} Y_k p_k D\,\mathbb{E}[J_{k,m}(x)]} p_m - 1\Bigg]\end{aligned} \tag{B.13}$$

Species i can invade is this quantity if positive when it is rare ($p_i \to 0$). When $D = 1$ (the case of interest), the above reduces to

$$Y_i \sum_{m\neq i} \frac{p_m}{Y_m\,\mathbb{E}[J_{m,m}(x)]p_m + \sum_{k\neq m} Y_k p_k\,\mathbb{E}[J_{k,m}(x)]} > 1 \tag{B.14}$$

To simplify the above equation, I ignore the term $Y_m\,\mathbb{E}[J_{m,m}(x)]p_m$ in the denominator and incorporate an additional term representing species m into the summation, yielding:

$$Y_i \sum_{m\neq i} \frac{p_m}{\sum_{k\neq i} Y_k p_k\,\mathbb{E}[J_{k,i}(x)]} > 1 \tag{B.15}$$

This simplification is equivalent to making the species identity of the tree previously occupying a patch (the tree that dies) irrelevant (i.e., JCEs only result from trees nearby the patch rather than the previous occupant of the patch). As long as neighborhood effects are somewhat strong – that is, so long as E_F is not very small – this assumption does not meaningfully affect the invasion criteria.

With this simplification, the denominator of equation (B.15) is no longer directly dependent

on m. That is, equation (B.15) can be rewritten as

$$Y_i \left(\sum_{m \neq i} p_m \right) \left(\frac{1}{\sum_{k \neq i} Y_k p_k \, \mathbb{E}[J_{k,i}(x)]} \right) > 1 \tag{B.16}$$

Because, by definition, $\sum_{m \neq i} p_m = 1$, the above equation can be rewritten as

$$Y_i > \sum_{k \neq i} Y_k p_k \, \mathbb{E}[J_{k,i}(x)] \tag{B.17}$$

Substituting the appropriate value for $\mathbb{E}[J_{k,i}(x)]$, the invasion criteria becomes

$$Y_i > \sum_{k \neq i} Y_k p_k e^{-(1-e^{-a}) p_k E_F} \tag{B.18}$$

recalling that $E_F = \pi r^2 g$. I take the linearization of $p_k e^{-(1-e^{-a}) p_k E_F}$ about the point $1/N$ where N is the number of species in the resident community (and thus, $1/N$ is the average abundance). This yields a close approximation of the expression so long as no species exhibits an abundance much greater than the mean abundance. For simplicity, let

$$J = (1 - e^{-a}) E_F \tag{B.19}$$

Taking the linearization yields

$$p_k e^{-J p_k} \approx \frac{e^{-\frac{J}{N}}}{N} + \frac{e^{-\frac{J}{N}} \left(p_k - \frac{1}{N} \right)}{N} \left(1 - \frac{J}{N} \right) \tag{B.20}$$

Substituting this into the original expression, the summation can be rearranged and broken up

into three parts:

$$e^{-\frac{J}{N}}\frac{1}{N}\sum_{k\neq i}Y_k + e^{-\frac{J}{N}}\left(1-\frac{J}{N}\right)\sum_{k\neq i}Y_k p_k - e^{-\frac{J}{N}}\left(1-\frac{J}{N}\right)\frac{1}{N}\sum_{k\neq i}Y_k \tag{B.21}$$

The first summation is straightforward to calculate:

$$e^{-\frac{J}{N}}\frac{1}{N}\sum_{k\neq i}Y_k = e^{-\frac{J}{N}}\overline{Y} \tag{B.22}$$

where $\overline{Y}$ is the mean intrinsic fitness of the community.

The second term can be expressed by using the property

$$\frac{1}{N}\sum_{m=1}^{N}A_m B_m = \overline{A}\times\overline{B} + \mathrm{Cov}(A,B) \tag{B.23}$$

I apply this property with respect to p and Y, noting that $\overline{p} = \frac{1}{N}$. Using this property and substituting A and B with p and Y for the first and second summations yields

$$\begin{aligned} e^{-\frac{J}{N}}\left(1-\frac{J}{N}\right)\sum_{k\neq i}p_k Y_k &= e^{-\frac{J}{N}}\left(1-\frac{J}{N}\right)N\frac{1}{N}\sum_{k\neq i}p_k Y_k \\ &= e^{-\frac{J}{N}}\left(1-\frac{J}{N}\right)N\left[\overline{Y}\frac{1}{N} + \mathrm{Cov}(p,Y)\right] \\ &= e^{-\frac{J}{N}}\left(1-\frac{J}{N}\right)\left[\overline{Y} + N\ \mathrm{Cov}(p,Y)\right] \end{aligned} \tag{B.24}$$

The third summation is easy to calculate:

$$-e^{-\frac{J}{N}}\left(1-\frac{J}{N}\right)\frac{1}{N}\sum_{k\neq i}Y_k = -e^{-\frac{J}{N}}\left(1-\frac{J}{N}\right)\overline{Y} \tag{B.25}$$

Adding all three summations together, the third summation term will cancel with the $\overline{Y}$ term of the second summation. Then, after some rearranging, I substitute $J = (1-e^{-a})E_F$ back into

the equation. This yields:

$$Y_i > \underbrace{\overline{Y} e^{-\left(1-e^{-a}\right)\frac{E_F}{N}}}_{\substack{\text{mean} \\ \text{JCE-fitness term}}} + \underbrace{N\text{Cov}(p, Y)\left(1 - \left(1 - e^{-a}\right)\frac{E_F}{N}\right)e^{-\left(1-e^{-a}\right)\frac{E_F}{N}}}_{\text{covariance-JCE term}} \tag{B.26}$$

which is the same as the expression given for the AF model in Table 1 of the manuscript.

5 Note on ODE approximation

To derive the ODE model, I took approximations of the expected values of $P_{i,i}(x)$ and $P_{i,k}(x)$. To do so, I took the expected abundance of $S_{i,i}(x)$, $S_{i,k}(x)$, and $S_{all,i}(x)$ with respect to space and then examined their quotients. Note that this assumes $\mathbb{E}[S_{i,k}(x)/S_{all,i}(x)] \approx \mathbb{E}[S_{i,k}(x)]/\,\mathbb{E}[S_{all,k}(x)]$ (I take the expectation of the numerator and denominator and then take the quotient). Using a Taylor Expansion about the mean,

$$\mathbb{E}\left[\frac{S_{i,k}(x)}{S_{all,i}(x)}\right] \approx \frac{\mathbb{E}[S_{i,k}(x)]}{\mathbb{E}[S_{all,k}(x)]} - \frac{\text{Cov}\big(S_{i,k}(x), S_{all,k}(x)\big)}{\mathbb{E}[S_{all,k}(x)]^2} + \text{Var}\big(S_{all,k}(x)\big)\frac{\mathbb{E}[S_{i,k}(x)]}{\mathbb{E}[S_{all,i}(x)]^3}$$

Because there are many species in the community, $S_{all,i}(x) >> S_{i,k}(x)$. This implies that the covariance term and the term containing $\mathbb{E}[S_{all,i}(x)]^3$ are close to zero. Additionally, $\text{Var}\big(S_{all,i}(x)\big)$ is likely small because it is assumed that dispersal is uniform across the community. Therefore, $\mathbb{E}[S_{i,k}(x)/S_{all,i}(x)] \approx \mathbb{E}[S_{i,k}(x)]/\,\mathbb{E}[S_{all,k}(x)]$ is likely a good approximation. I rely on the quantitative similarity of the SEM and ODE model to validate this assumption.

6 Figures

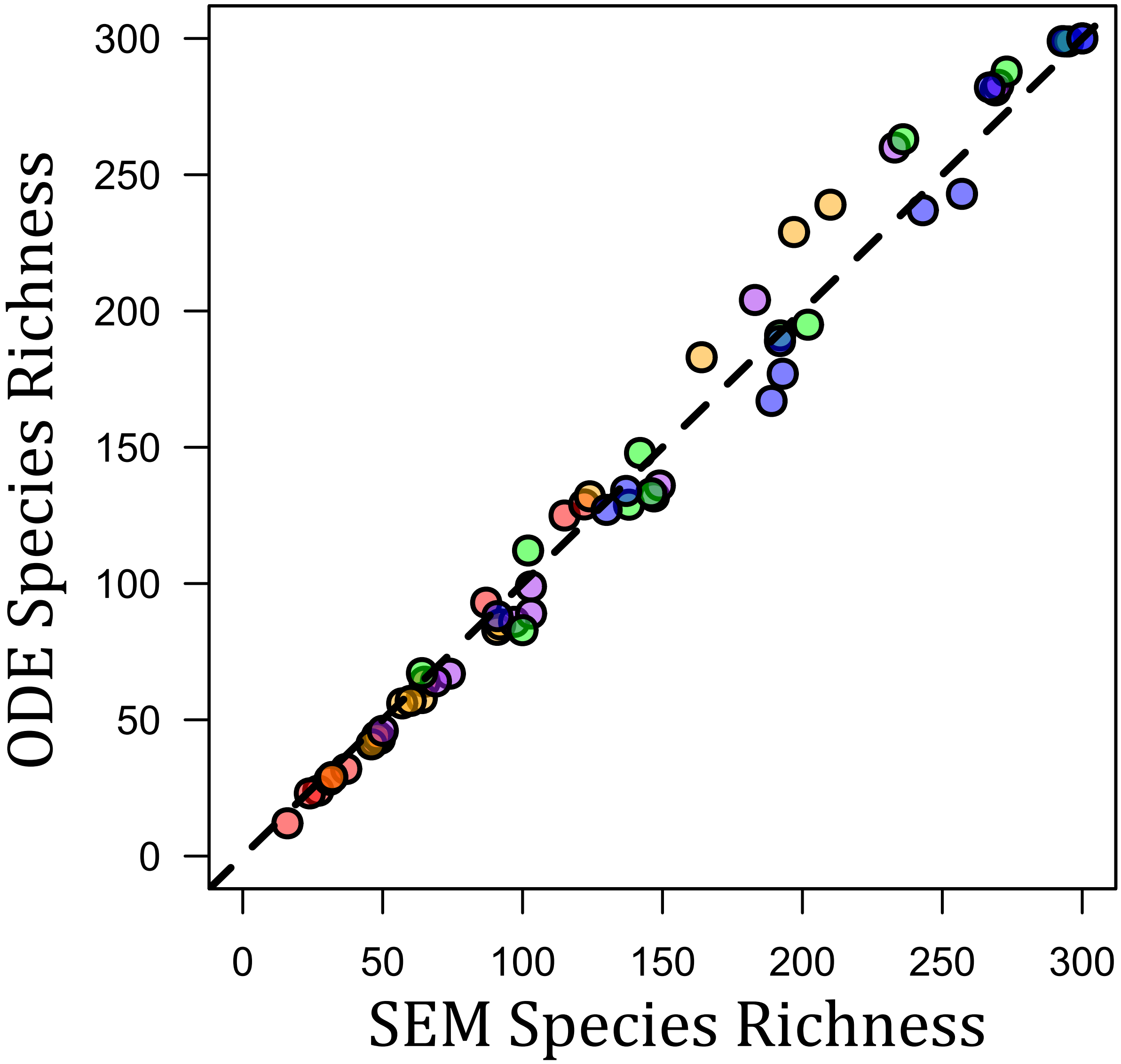

Fig. B1 ODE model validation. The figures compare species richness between SEM and ODE model simulations under identical parameterizations. The dashed line is the one-to-one line (points on the line represent when the SEM and ODE yield the exact same diversity output). Red points are when $E_F = 9$, orange/yellow points are when $E_F = 25$, purple points are when $E_F = 49$, green points are when $E_F = 81$, and blue points are when $E_F = 121$. To a first approximation, the ODE model yields the same output as the SEM.

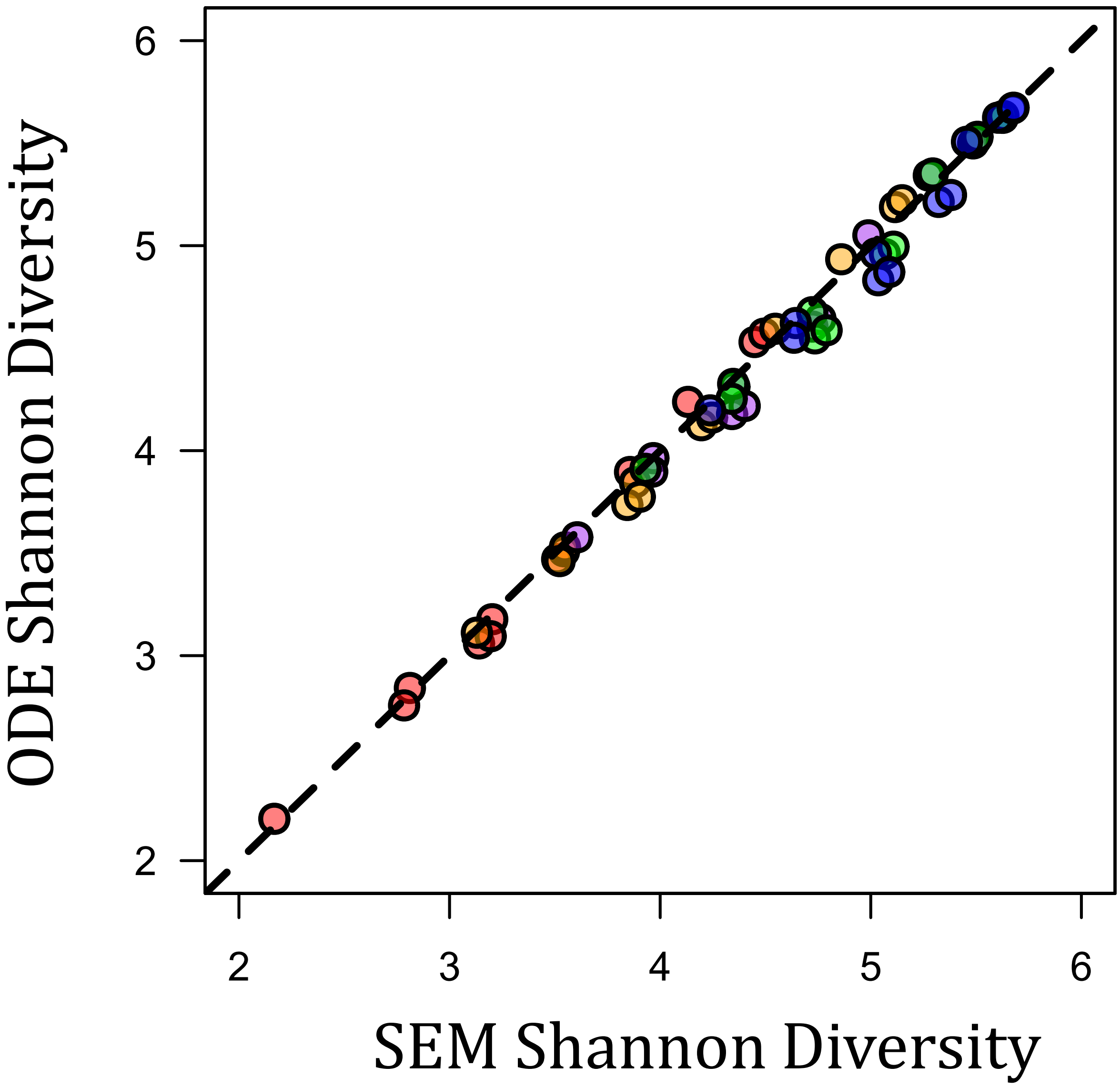

Fig. B2: The same as the Fig. B1, but showing Shannon Diversity instead of species richness. As in the above case, the SEM and ODE model yield very similar outputs.

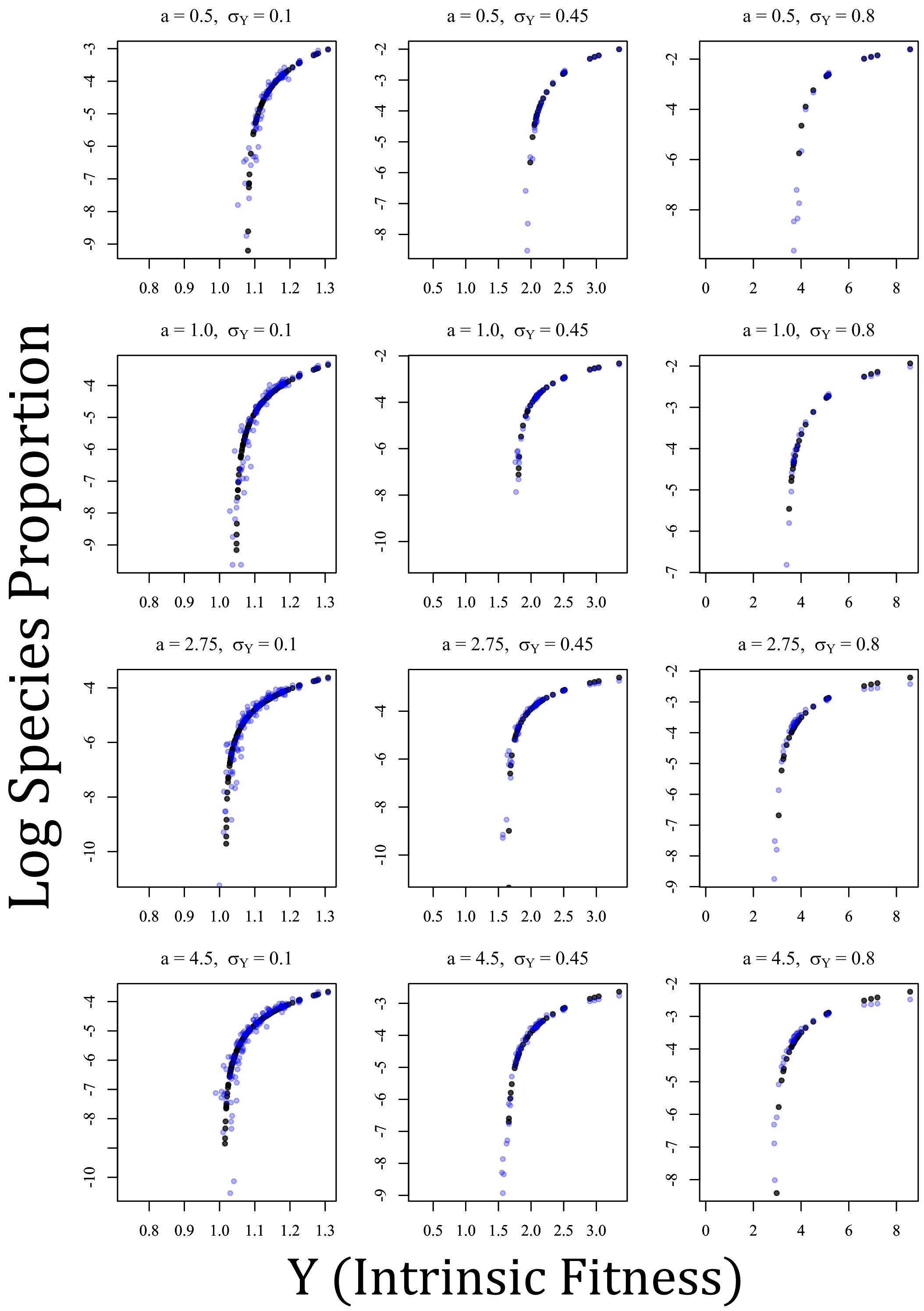

Fig. B3 Comparisons between identical parameterizations of the ODE approximation (black) and SEM (blue) outputs under nine parameter values when species vary in intrinsic fitness (Y). They-axis depicts the log-proportion of each species and the x-axis depicts Y of each species. These parameter values span the most of the parameter space explored in Fig. 4 of the main text. In all plots, $E_F = 9$, $g = 0.2$, and $D = 1.0$. Other relevant parameters are listed on each plot.

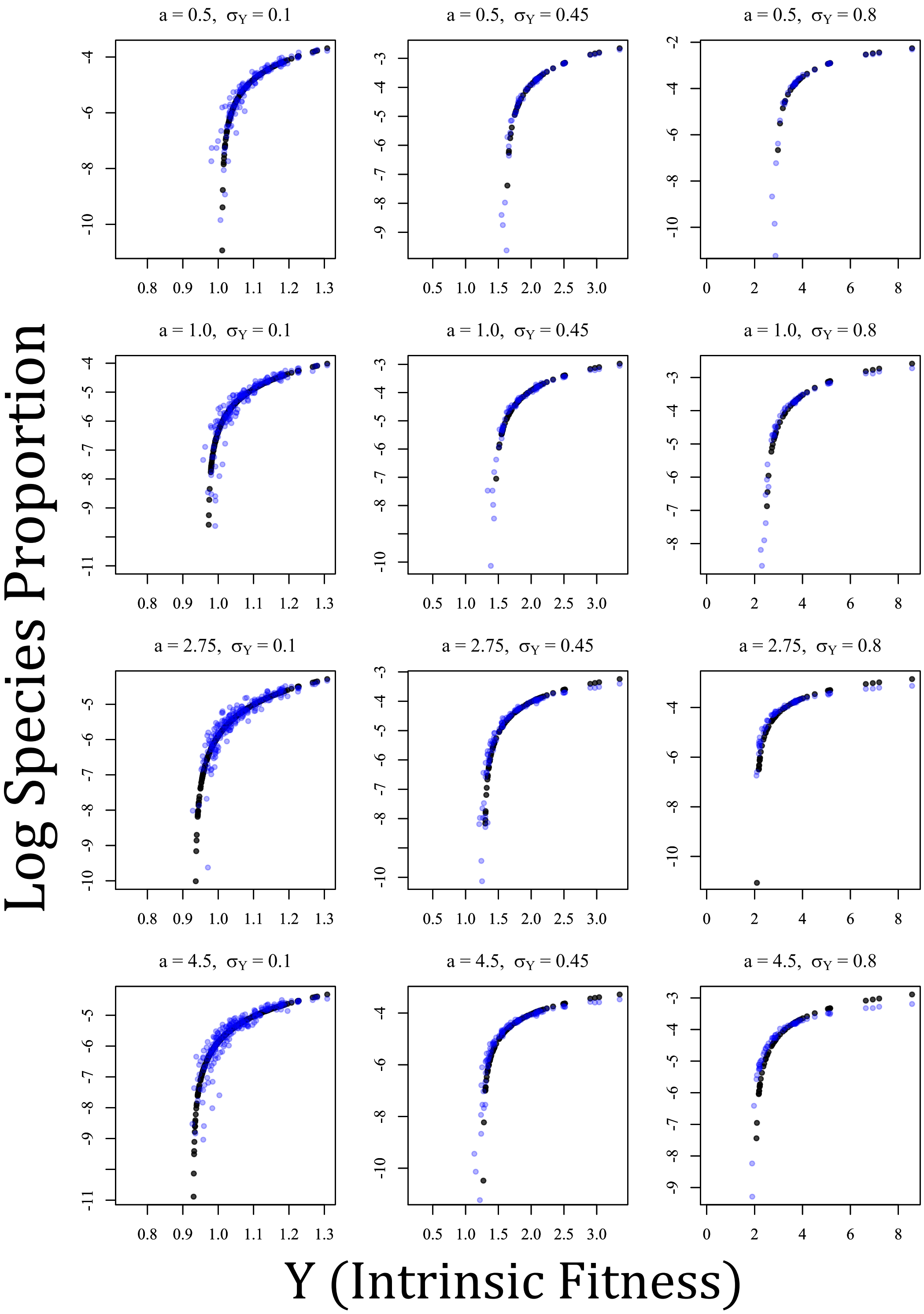

Fig. B4 The same format as Fig. B3, but with $E_F = 25$.

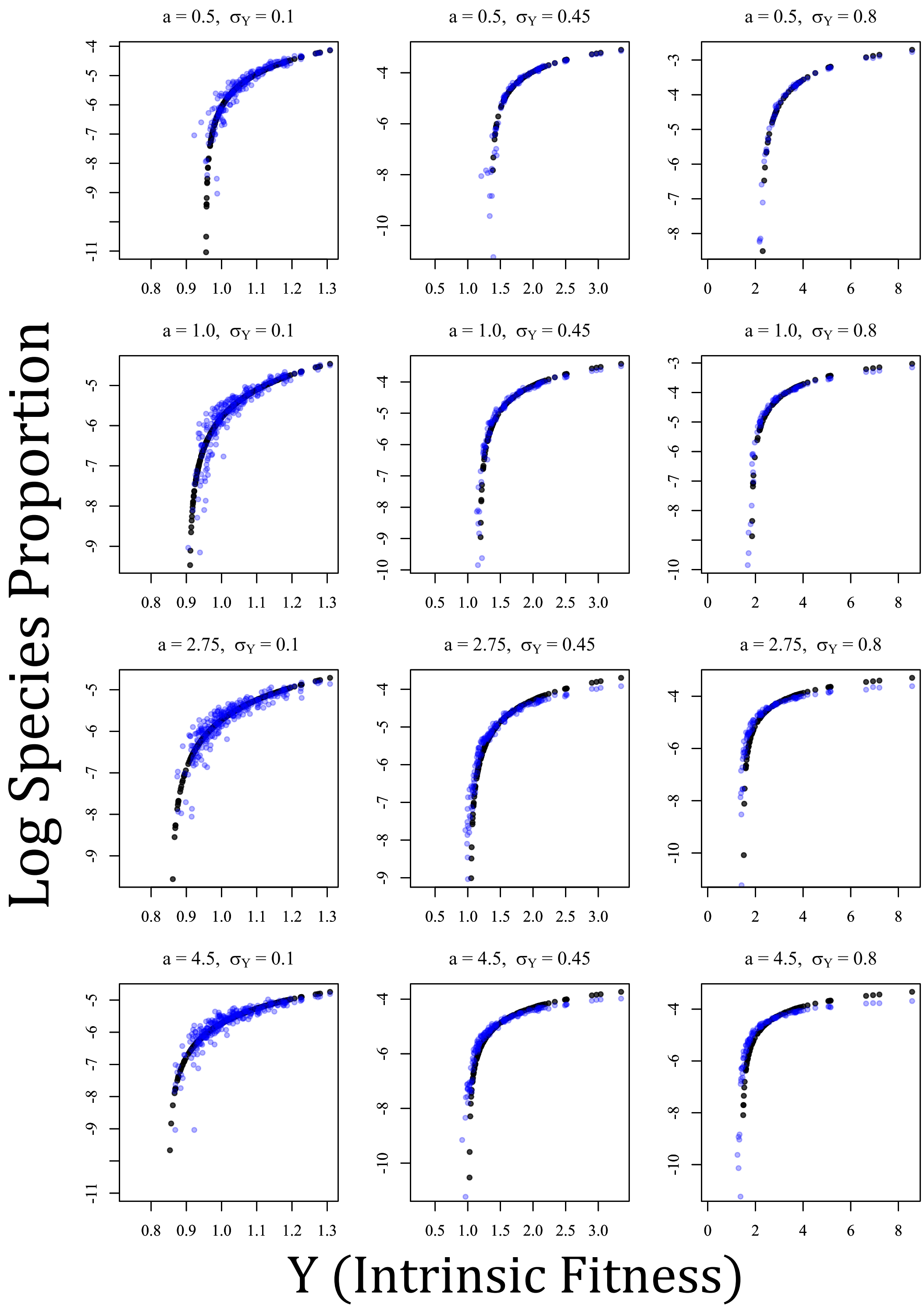

Fig. B5 The same format as Fig. B2, but with $E_F = 49$.

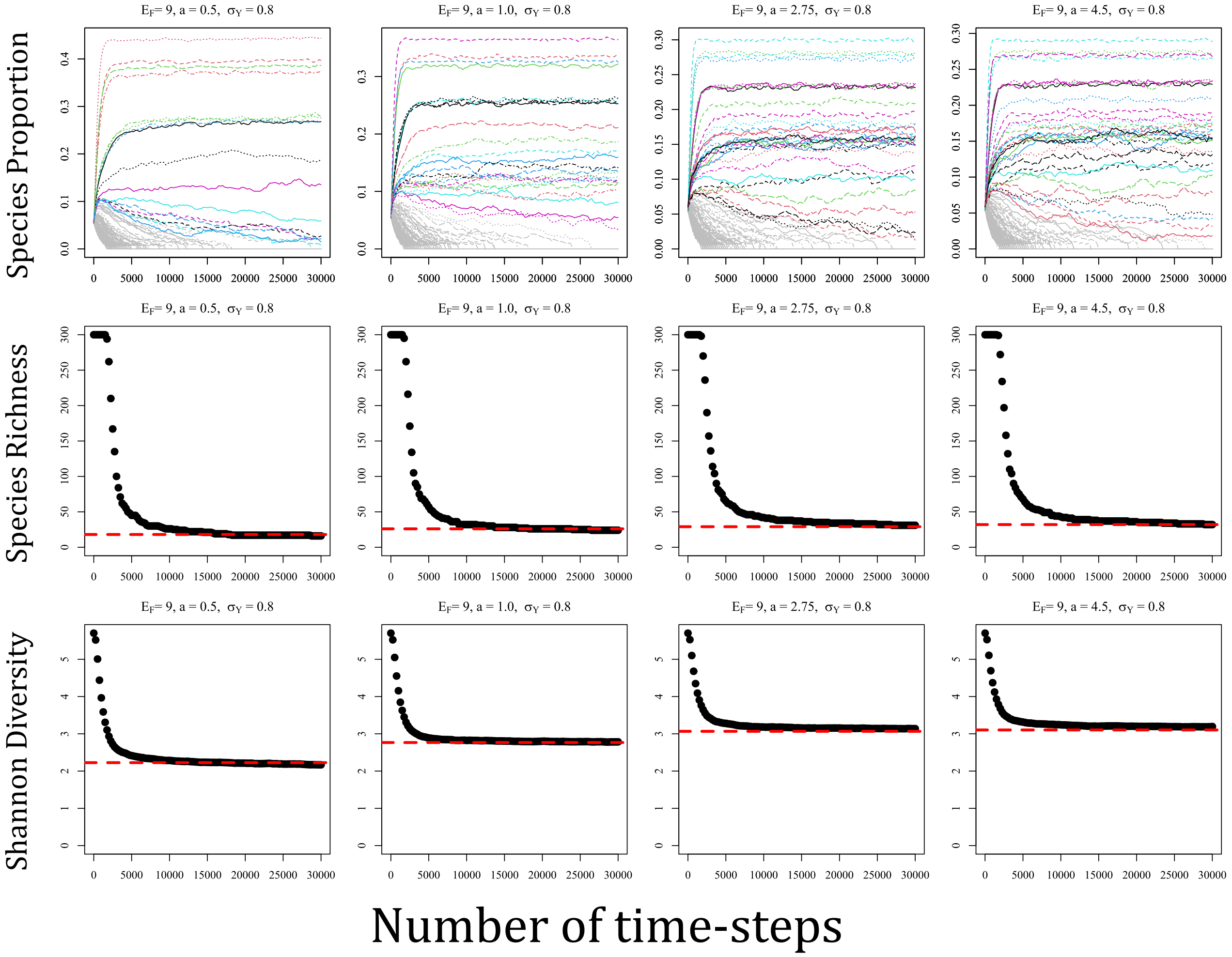

Fig. B6 Examples of the SEM simulation time series outputs, species richness over time in the simulations, and Shannon diversity over time in the simulations. The top row shows examples of the time series outputs of the SEMs. The x-axis is time and the y-axis is each species' proportion. Proportions have been square-root transformed to aid visualization. Colored trajectories indicate species that persisted throughout the simulation; grey trajectories indicate species that went extinct. Parameters are listed on each plot. Dynamics as shown are typical examples from the SEMs. Most species settle into a relatively stable pseudo-equilibrium, while lower abundance species fluctuate due to drift. The second row shows the number of persisting species in the community as a function of time. Each panel corresponds to the plot above it. Most species that go extinct do so in the early stages of the dynamics. Therefore, the vast majority of persisting species likely persist deterministically. The dashed red line is the diversity maintained by the ODE under the same parameterization. All SEMs saturate, approximately, to the dashed line. The third row is the same as the second row, except it shows Shannon diversity instead of species richness.

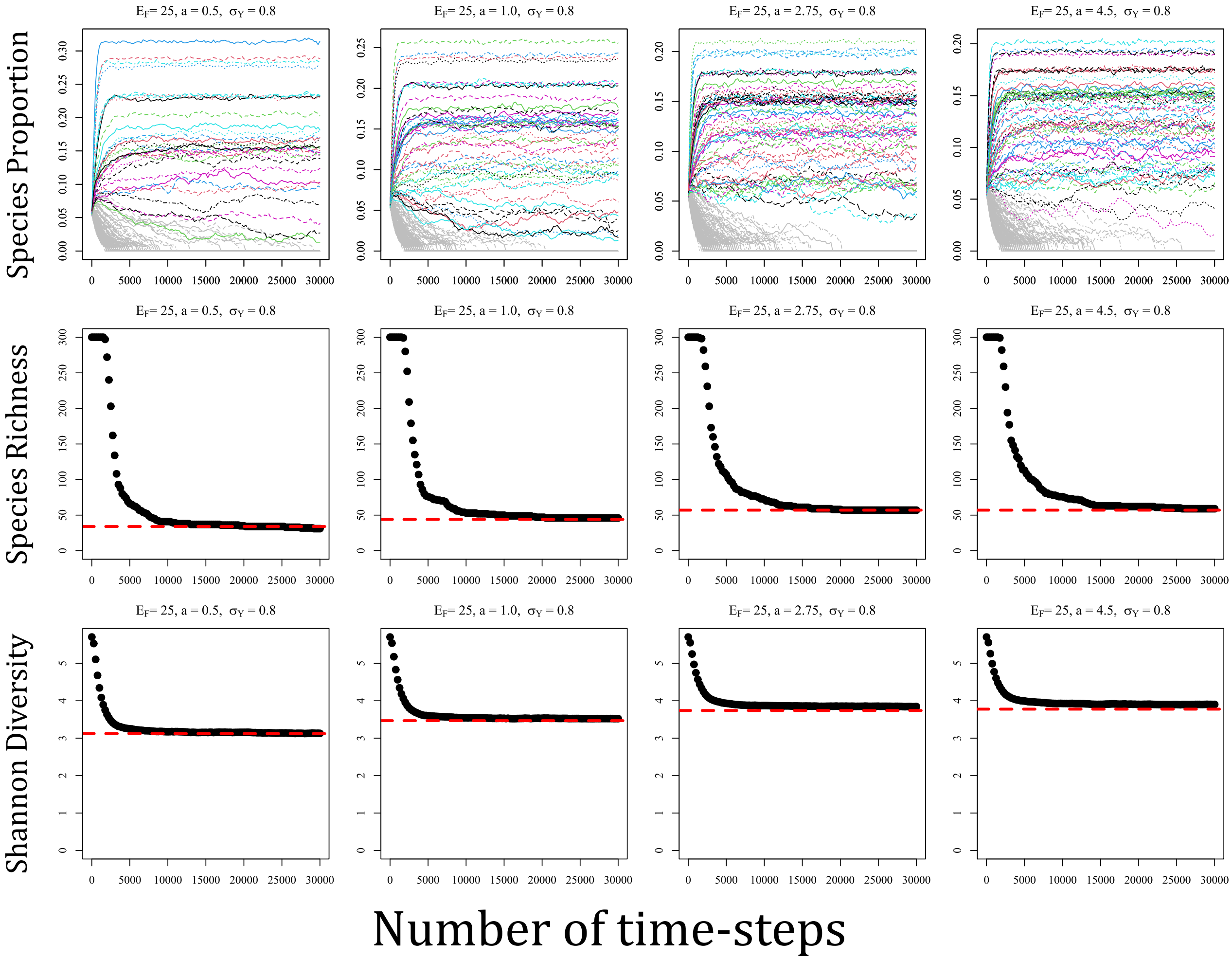

Fig. B7 The same as Fig. B6, but with $E_F = 25$.

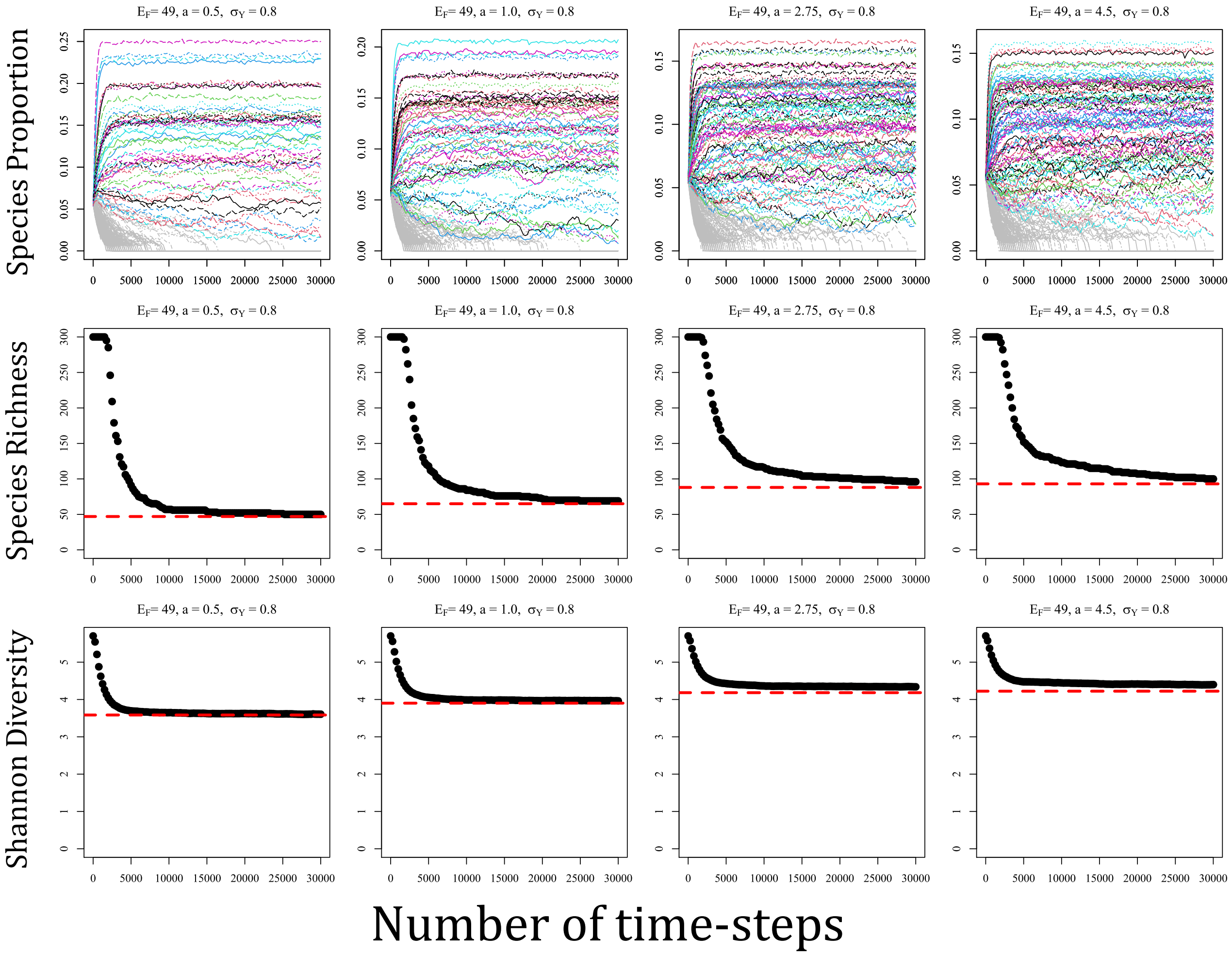

Fig. B8 The same as Fig. B6, but with $E_F = 49$.
