# Supplementary material for: The functional form of specialised predation affects whether Janzen–Connell effects can prevent competitive exclusion
Source: Ecol Lett. 2022 Apr 26;25(6):1458–70. doi: 10.1111/ele.14014 (PMC9324109; doi:10.1111/ele.14014)
Supplement: Supplementary file 3 — Supplementary Material [file ELE-25-1458-s006.pdf]

Appendix C: non-additive−distance-decay SEM and ODE approximation

Contents

1 Introduction **2**

2 Spatially Explicit Model and derivation of ODE approximation **2**

2.1 SEM 2

2.2 ODE Model 4

3 Comparison between ODE model and SEM model **6**

3.1 ODE and SEM parameterization 6

3.2 Results of comparison 7

4 Derivation of invasion criteria **7**

5 Note on ODE approximation **11**

6 Figures **11**

1 Introduction

In this Appendix, I analyze the Non-additive−Distance-decay (ND) model presented in the main text. This Appendix is composed of three main sections: **(1)** I introduce a spatially explicit model (SEM) of the ND model. I then demonstrate that taking the expected offspring abundances on each patch yields the ODE model discussed in the main text. **(2)** I provide outputs of the ODE model and the SEM model under the same parameterizations. I show the outputs are very similar, hence demonstrating that the ODE is a sufficiently accurate approximation of the SEM. **(3)** I provide the derivation of the approximate invasion criteria of the ND model (Table 1 in the main text). The structure of this Appendix is identical to that of Appendices A and B.

2 Spatially Explicit Model and derivation of ODE approximation

In this section, I discuss the SEM, briefly review within-patch dynamics from the main text, and show the derivation of the ODE approximation. I assume the reader is familiar with the general model discussed in the main text.

2.1 SEM

I developed a spatially explicit model that integrates the non-additive−distance-decay functional form. The model consists of a community on a grid of $L \times L$ patches (M total patches, $M = L^2$) modeled as a torus to avoid edge effects. A single tree is present on every space on the grid. At each time-step, each tree dies with probability δ and tree replacement occurs via a lottery based on offspring abundances on each patch. The ND model assumes that predation pressure is non-additive and that predation pressure declines exponentially with distance. As

noted in the main text, offspring abundances on each patch type are equal to

$$\begin{aligned} S_{i,i}(x) &= Y_i\big[(1-D)+p_iD\big]J_{i,i}(x) \\ S_{i,k}(x) &= Y_ip_iDJ_{i,k}(x) \\ S_{all,i}(x) &= \sum_{n=1}^{N} S_{n,i}(x) \end{aligned} \tag{C.1}$$

where $S_{A,B}(x)$ is the offspring abundance of species A on a patch occupied by species B at location x, $J_{i,i}(x)$ and $J_{i,k}(x)$ are how JCEs affect offspring surivorship, p_i is the proportion of species i in the population, Y_i is the intrinsic fitness of species i, and D is the dispersal proportion. For the ND model:

$$\begin{aligned} J_{i,i}(x) &= \exp\big[-a\big] \\ J_{i,k}(x) &= \exp\Big[-ae^{-\min(x_i)/v}\Big] \end{aligned} \tag{C.2}$$

where $\min(x_i)$ is distance between the focal patch and the closest individual of species i. Offspring abundances are determined by the following equations:

$$\begin{aligned} S_{i,i}(x) &= Y_i\big[(1-D)+p_iD\big]e^{-a} \\ S_{i,k}(x) &= Y_ip_iD\ \exp\Big[-ae^{-\min(x_i)/v}\Big] \\ S_{all,i}(x) &= Y_i\big[(1-D)+p_iD\big]e^{-a}+D\sum_{k\neq i}Y_kp_k\ \exp\Big[-ae^{-\min(x_k)/v}\Big] \end{aligned} \tag{C.3}$$

where $S_{A,B}(x)$ is the offspring abundance of species A on a patch occupied by species B at location x. v defines rate at which predataion declines with distance (higher v indicates a lower rate of predation decay). The lottery is determined by the relative abundance of offspring. Let $P_{A,B}(x)$ be the probability species A colonizes a patch previously occupied by species B at location x. Then, $P_{i,i}(x) = S_{i,i}(x)/S_{all,i}(x)$ and $P_{i,k}(x) = S_{i,k}(x)/S_{all,k}(x)$. For the SEM, patch-specific JCEs

are determined by the euclidean distance between patches. Distances are calculated between the center points of each patch and it is assumed offspring are at the center of each patch.

2.2 ODE Model

To derive the ODE model, I take approximations of the expected values of $P_{i,i}(x)$ and $P_{i,k}(x)$. To do so, I take the expected abundance of $S_{i,i}(x)$, $S_{i,k}(x)$, and $S_{all,i}(x)$ and then take their quotients. Expectations are taken with respect to space. Using this, I derive the the ODE approximation

$$\frac{dp_i}{dt} = \delta\left[\frac{\mathbb{E}[S_{i,i}(x)]}{\mathbb{E}[S_{all,i}(x)]}p_i + \sum_{k\neq i}\frac{\mathbb{E}[S_{i,k}]}{\mathbb{E}[S_{all,k}(x)]}p_k - p_i\right] \tag{C.4}$$

that captures the behavior of the SEM. See "Note" at the end of this Appendix for additional information about the assumptions of this approximation.

JCEs in the SEM are implemented using the spatial proximity of trees that, computationally, is stored in a matrix. The deterministic ODE model is spatially implicit. Therefore, it is necessary to use an approximation of the terms that do not require spatial information. In particular, the quantity $S_{i,k}(x)$, which contains the term

$$\exp\left[-ae^{-\min(x_i)/v}\right] \tag{C.5}$$

must be approximated as a spatially implicit term. To do so, I approximate the expected survival of each species' offspring on a random patch by evaluating

$$\mathbb{E}\left[\exp\left[-ae^{-\min(x_i)/v}\right]\right].$$

To evaluate this quantity, I use a Taylor Expansion to evaluate the first moment approxima-

tion the function of $f(X) = \exp\big[- ae^{-X/v}\big]$. The approximation is defined by:

$$\begin{aligned} \mathbb{E}[f(X)] &\approx f(\mathbb{E}[X]) + \frac{1}{2} f''(\mathbb{E}[X])\, \mathbb{E}\left[(X - \mathbb{E}[X])^2\right] \\ &= f(\mathbb{E}[X]) + \frac{1}{2} f''(\mathbb{E}[X]) \sigma_X^2 \end{aligned} \tag{C.6}$$

where X is the a random variable of the nearest neighbor distribution in space (i.e. the probability distribution of $\min(x_i)$) and σ_X^2 is the variance of X. Assuming individuals are approximately randomly distributed in space, X follows a Rayleigh distribution with probability distribution function $f_X = 2\pi g p_i x e^{-g p_i \pi x^2}$ where x represents radial distance from a point in 2D space (Clark & Evans, 1954). Relevant to the approximation,

$$\begin{aligned} \mathbb{E}[X] &= \frac{1}{2\sqrt{p_i g}} \\ \sigma_X^2 &= \left(1 - \frac{\pi}{4}\right) \frac{1}{\pi g p_i} \end{aligned} \tag{C.7}$$

for the Rayleigh distribution. In addition,

$$f''(X) = \frac{1}{v^2} a e^{-ae^{-\frac{X}{v}}} e^{-\frac{X}{v}} \left(ae^{-\frac{X}{v}} - 1 \right) \tag{C.8}$$

Putting it all together and doing some rearranging yields

$$\mathbb{E}[f(X)] \approx e^{-ae^{-\sqrt{\frac{\pi}{2E_D p_i}}}} \left(1 + ae^{-\sqrt{\frac{\pi}{2E_D p_i}}} \left(ae^{-\sqrt{\frac{\pi}{2E_D p_i}}} - 1 \right) \left(1 - \frac{\pi}{4} \right) \frac{1}{p_i E_D} \right) \tag{C.9}$$

where

$$E_D = 2\pi g v^2$$

Plugged into the appropriate offspring abundance equations, the approximation yields

$$\begin{aligned}
\mathbb{E}[S_{i,i}(x)] &= Y_i\big[(1-D)+p_iD\big]e^{-a} \\
\mathbb{E}[S_{i,k}(x)] &= Y_iDp_ie^{-ae^{-\sqrt{\frac{\pi}{2E_Dp_i}}}}\left(1+ae^{-\sqrt{\frac{\pi}{2E_Dp_i}}}\left(ae^{-\sqrt{\frac{\pi}{2E_Dp_i}}}-1\right)\left(1-\frac{\pi}{4}\right)\frac{1}{p_iE_D}\right)
\end{aligned} \tag{C.10}$$

which are identical to the ND model offspring abundance expressions in the main text. $\mathbb{E}[S_{all,i}(x)] = \sum_{n=1}^{N}\mathbb{E}[S_{n,i}(x)]$.

3 Comparison between ODE model and SEM model

In this section, I describe simulations that compare the ODE model to the SEM. I demonstrate that the SEM and ODE model yield highly similar outputs of species abundance and species richness. I provide 36 comparisons of the SEM to the ODE (12 cases in which $v = 5$, 12 cases in which $v = 7.5$, and 12 cases in which $v = 10$). Parameter values were chosen to approximately encapsulate the range of parameters shown in the main text.

3.1 ODE and SEM parameterization

Each SEM simulation began with 300 species at equal abundance, with individuals randomly distributed throughout the community. Simulations were conducted on a 275×275 torus (thus containing 275^2 individual trees). I used the following parameters: $Y \sim \text{lognormal}[\mu = 0, \sigma_Y]$ with $\sigma_Y \sim \{0.1, 0.45, 0.8\}$ and $a \sim \{0.5, 1.0, 2.75, 4.5\}$. In all simulations, $g = 0.20$ and $D = 1$. I tested each of the 12 parameter combinations of σ_Y and a with $v \sim \{5, 7.5, 10\}$. This generated 36 outputs. Simulations were run for about 65 generations, sufficient time for the community to approximately reach equilibrium without drift dominating the dynamics of the lower abundance species. See Figs. C6-C8 for typical outputs of the SEM time series dynamics.

A corresponding set of 36 ODE simulations were run using the same parameterizations as the SEM. I compared the outputs of the SEM and ODE model in terms of species diversity, species abundance, and Shannon diversity. I considered a species to be extinct if it had less than 1

individual at any point of the simulation. This was implemented directly in the SEM; for the ODE model, I assumed a species, i, to be extinct if $p_i^* < 1/275^2$ where p_i^* is the equilibrium proportion of species i. Note that these simulations do not attempt to demonstrate the long-term resistance against extinction due to drift. Rather, they demonstrate that the ODE model and SEM yield similar outputs of expected species abundance and richness given the same parameterization.

3.2 Results of comparison

ODE model and SEM produced very similar species richness and Shannon diversity (Figs. C1, C2). The ODE model and SEM also produced very similar species proportions (Figs. C3-C5). To quantify the quality of the approximation, I calculated the mean difference in species richness between the ODE model and SEM, ΔR:

$$\Delta R = \frac{1}{S}\sum_{k=1}^{S}(R^k_{\text{SEM}} - R^k_{\text{ODE}}) \tag{C.11}$$

where S is the number of simulations, and R^k_{SEM} and R^k_{ODE} are the species richness of the k_{th} simulation of the SEM and ODE model, respectively. I also examined the r^2 (coefficient of determination) between SEM and ODE species richness. For the comparisons, $\Delta R = -3.37$ and $r^2 = 0.98$. Overall, the ODE provides a highly similar, albeit non-exact, estimation of species diversity. Error in which the ODE model predicted greater diversity than the SEM is most likely due to stochastic extinction due to drift. This is particularly likely when diversity is high, where the expected abundance of each species is correspondingly smaller. Cases in which the ODE model predicted lower species richness are likely due to incomplete transient dynamics of the SEM.

4 Derivation of invasion criteria

In this section, I derive the approximate invasion criteria of the ND model when species experience inter-specific variation in intrinsic fitness (Y) with $D = 1$. Compared to the other

sections, the algebra of this is quite messy. Therefore, I provide a brief description of the steps. Interested readers can verify the abbreviated algebra or contact the author for details. For simplicity, let

$$\mathbb{E}[J(p_i)] = e^{-ae^{-\sqrt{\frac{\pi}{2E_D p_i}}}} \left(1 + ae^{-\sqrt{\frac{\pi}{2E_D p_i}}} \left(ae^{-\sqrt{\frac{\pi}{2E_D p_i}}} - 1\right) \left(1 - \frac{\pi}{4}\right) \frac{1}{p_i E_D}\right)$$

Then, the per capita growth rate of species i, substituting in the offspring abundance values, is

$$\begin{aligned} \frac{1}{p_i}\frac{dp_i}{dt} = r_i = \delta \Bigg[& \frac{Y_i\big[(1-D) + p_i d\big]e^{-a}\,\mathbb{E}[J(p_i)]}{Y_i\big[(1-D) + p_i D\big]e^{-a}J(p_i) + D\sum_{k\neq i} Y_k p_k\,\mathbb{E}[J(p_k)]} \\ & + Y_i D \sum_{m\neq i} \frac{1}{Y_m\big[(1-D) + p_m D\big]e^{-a}\,\mathbb{E}[J(p_m)] + D\sum_{k\neq m} Y_k p_k\,\mathbb{E}[J(p_k)]} p_k - 1\Bigg] \end{aligned} \tag{C.12}$$

Species i can invade is this quantity if positive when it is rare ($p_i \to 0$). When $D = 1$ (the case of interest), the above reduces to

$$Y_i \sum_{m\neq i} \frac{1}{Y_m p_m e^{-a} + \sum_{k\neq m} Y_k p_k\,\mathbb{E}[J(p_k)]} p_m > 1 \tag{C.13}$$

To further simplify the above equation, I remove the term $Y_m p_m e^{-a}$ and substitute it with $Y_m p_m\,\mathbb{E}[J(p_m)]$ in the denominator of the summation. Doing so yields

$$Y_i \sum_{m\neq i} \frac{p_m}{\sum_{k\neq i} Y_k p_k\,\mathbb{E}[J(p_k)]} > 1 \tag{C.14}$$

This simplification is equivalent to making the species identity of the tree previously occupying a patch (the tree that dies) irrelevant (i.e., JCEs only result from trees nearby the patch rather than the previous occupant of the patch). As long as predation occurs over a non-trivial distance – that is, so long as v is not very small – this assumption should not meaningfully affect the invasion criteria.

With this simplification, the denominator of equation (C.14) is no longer directly dependent on m and equation (C.14) can be rewritten as

$$Y_i\left(\sum_{m\neq i} p_m\right)\left(\frac{1}{\sum_{k\neq i} Y_k p_k\,\mathbb{E}[J(p_k)]}\right) > 1 \tag{C.15}$$

Because $\sum_{m\neq i} p_m = 1$, the inequality can be written as

$$Y_i > \sum_{k\neq i} Y_k p_k\,\mathbb{E}[J(p_k)] \tag{C.16}$$

I then take the linearization of $p_k\,\mathbb{E}[J(p_k)]$ around the point $1/N$ where N is the number of species in the resident community yields a close approximation of the expression so long as inter-specific variation in p_k is not very large. This yields

$$p_k\,\mathbb{E}[J(p_k)] \approx \frac{1}{N}J\left(\frac{1}{N}\right) + J'\left(\frac{1}{N}\right)\left(p_k - \frac{1}{N}\right) \tag{C.17}$$

where $J'\left(\frac{1}{N}\right)$ is the derivative of $\mathbb{E}[J(p_k)]$ with respect to p_k at the point $1/N$. Plugging this into the summation yields:

$$\frac{1}{N}J\left(\frac{1}{N}\right)\sum_{k\neq i} Y_k + J'\left(\frac{1}{N}\right)\sum_{k\neq i} Y_k p_k - J'\left(\frac{1}{N}\right)\frac{1}{N}\sum_{k\neq i} Y_k \tag{C.18}$$

The first and third summations in the above expression are easy to evaluate:

$$\frac{1}{N}J\left(\frac{1}{N}\right)\sum_{k\neq i} Y_k = J\left(\frac{1}{N}\right)\overline{Y} \tag{C.19}$$

and

$$-J'\left(\frac{1}{N}\right)\frac{1}{N}\sum_{k\neq i} Y_k = -J'\left(\frac{1}{N}\right)\overline{Y} \tag{C.20}$$

where $\overline{Y}$ is the mean fitness of the resident community.

The second summation term, which contains a $Y_k p_k$ term can be evaluated using the property

$$\frac{1}{N}\sum_{m=1}^{N} A_m B_m = \overline{A} \times \overline{B} + \mathrm{Cov}(A, B) \tag{C.21}$$

substituting A and B with p and Y. Ultimately, doing this procedure and rearranging terms yields:

$$J'\left(\frac{1}{N}\right)\sum_{k\neq i} Y_k p_k = J'\left(\frac{1}{N}\right)\left[\overline{Y} + N\mathrm{Cov}(p, Y)\right] \tag{C.22}$$

noting that $\overline{p} = 1/N$.

I then add all the summation terms together. Notably, the third summation term, $-J'\left(\frac{1}{N}\right)\overline{Y}$, and the $\overline{Y}$ term from the second summation term, $J'\left(\frac{1}{N}\right)\overline{Y}$, will cancel. After some algebra and substituting the correct values of $J\left(\frac{1}{N}\right)$ and $J'\left(\frac{1}{N}\right)$, the invasion criteria can be expressed as

$$\begin{aligned} Y_i >& \underbrace{\overline{Y} e^{-ae^{-\sqrt{\frac{\pi N}{2E_D}}}}\left(1 + ae^{-\sqrt{\frac{\pi N}{2E_D}}}\left(ae^{-\sqrt{\frac{\pi N}{2E_D}}} - 1\right)\left(1 - \frac{\pi}{4}\right)\frac{N}{E_D}\right)}_{\text{mean JCE-fitness term}} \\ &+ N\mathrm{Cov}(p, Y)\times \\ &\underbrace{e^{-ae^{-\sqrt{\frac{\pi N}{2E_D}}}}\left[1 - \frac{\left(1 - \frac{\pi}{4}\right) a^3 N\sqrt{\frac{\pi N}{2E_D}} e^{-3\sqrt{\frac{\pi N}{2E_D}}}}{2E_D} + \frac{3\left(1 - \frac{\pi}{4}\right) a^2 N\sqrt{\frac{\pi N}{2E_D}} e^{-2\sqrt{\frac{\pi N}{2E_D}}}}{2E_D} - \frac{a\sqrt{\frac{\pi N}{2E_D}}\left(E_D + \left(1 - \frac{\pi}{4}\right) N\right) e^{-\sqrt{\frac{\pi N}{2E_D}}}}{2E_D}\right]}_{\text{covariance-JCE term}} \end{aligned} \tag{C.23}$$

which is identical to the expression in Table 1 in the main text with the covariance-JCE term added.

5 Note on ODE approximation

To derive the ODE model, I took approximations of the expected values of $P_{i,i}(x)$ and $P_{i,k}(x)$. To do so, I took the expected abundance of $S_{i,i}(x)$, $S_{i,k}(x)$, and $S_{all,i}(x)$ with respect to space and then examined their quotients. Note that this assumes $\mathbb{E}[S_{i,k}(x)/S_{all,i}(x)] \approx \mathbb{E}[S_{i,k}(x)]/\,\mathbb{E}[S_{all,k}(x)]$ (I take the expectation of the numerator and denominator and then take the quotient). Using a Taylor Expansion about the mean,

$$\mathbb{E}\left[\frac{S_{i,k}(x)}{S_{all,i}(x)}\right] \approx \frac{\mathbb{E}[S_{i,k}(x)]}{\mathbb{E}[S_{all,k}(x)]} - \frac{\mathrm{Cov}\big(S_{i,k}(x), S_{all,k}(x)\big)}{\mathbb{E}[S_{all,k}(x)]^2} + \mathrm{Var}\big(S_{all,k}(x)\big)\frac{\mathbb{E}[S_{i,k}(x)]}{\mathbb{E}[S_{all,i}(x)]^3}$$

Because there are many species in the community, $S_{all,i}(x) >> S_{i,k}(x)$. This implies that the covariance term and the term containing $\mathbb{E}[S_{all,i}(x)]^3$ are close to zero. Additionally, $\mathrm{Var}\big(S_{all,i}(x)\big)$ is likely small because it is assumed that dispersal is uniform across the community. Therefore, $\mathbb{E}[S_{i,k}(x)/S_{all,i}(x)] \approx \mathbb{E}[S_{i,k}(x)]/\,\mathbb{E}[S_{all,k}(x)]$ is likely a good approximation. I rely on the quantitative similarity of the SEM and ODE model to validate this assumption.

References

Clark, P.J. & Evans, F.C. (1954). Distance to nearest neighbor as a measure of spatial relationships in populations. *Ecology*, 35, 445–453.

6 Figures

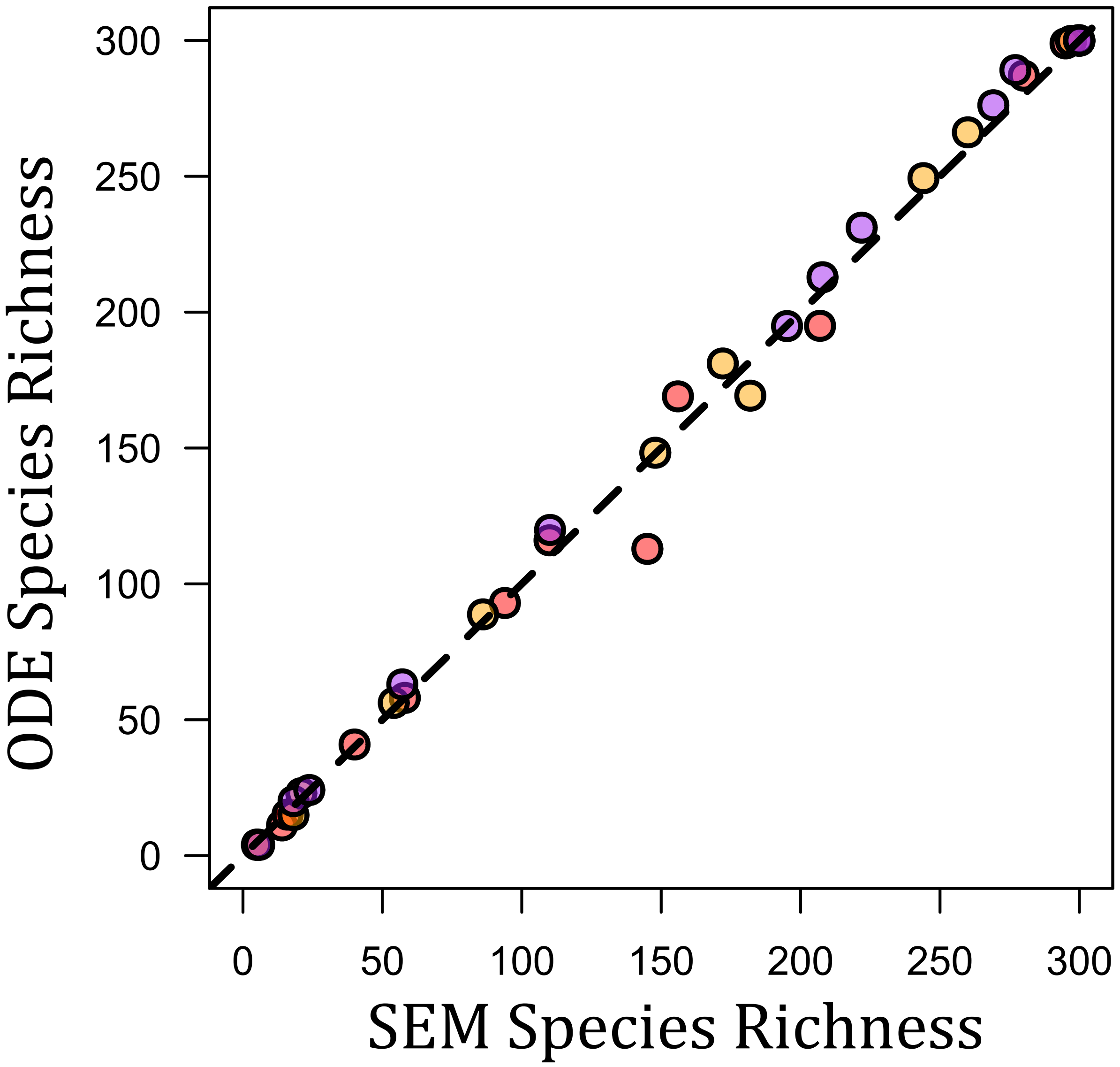

Fig. C1 ODE model validation. The figure compares species richness between SEM and ODE model simulations under identical parameterizations. The dashed line is the one-to-one line (points on the line represent when the SEM and ODE yield the exact same diversity output). Red points are when $v = 5$, orange/yellow points are when $v = 7.5$, and purple points are when $v = 10$. To a first approximation, the ODE model yields the same output as the SEM.

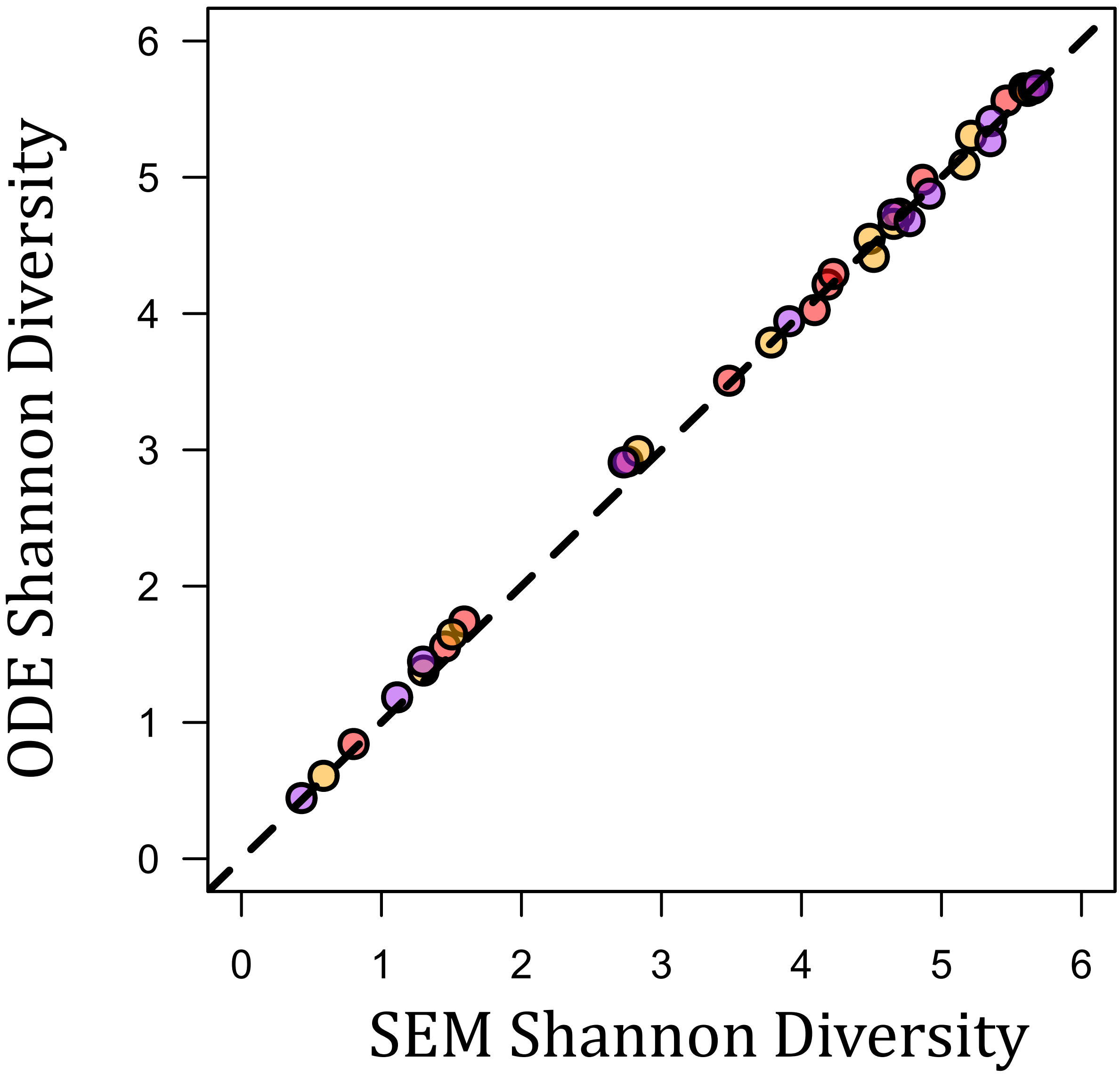

Fig. C2 The same as the Fig. C1, but showing Shannon Diversity instead of species richness. As in the above case, the SEM and ODE model yield very similar outputs.

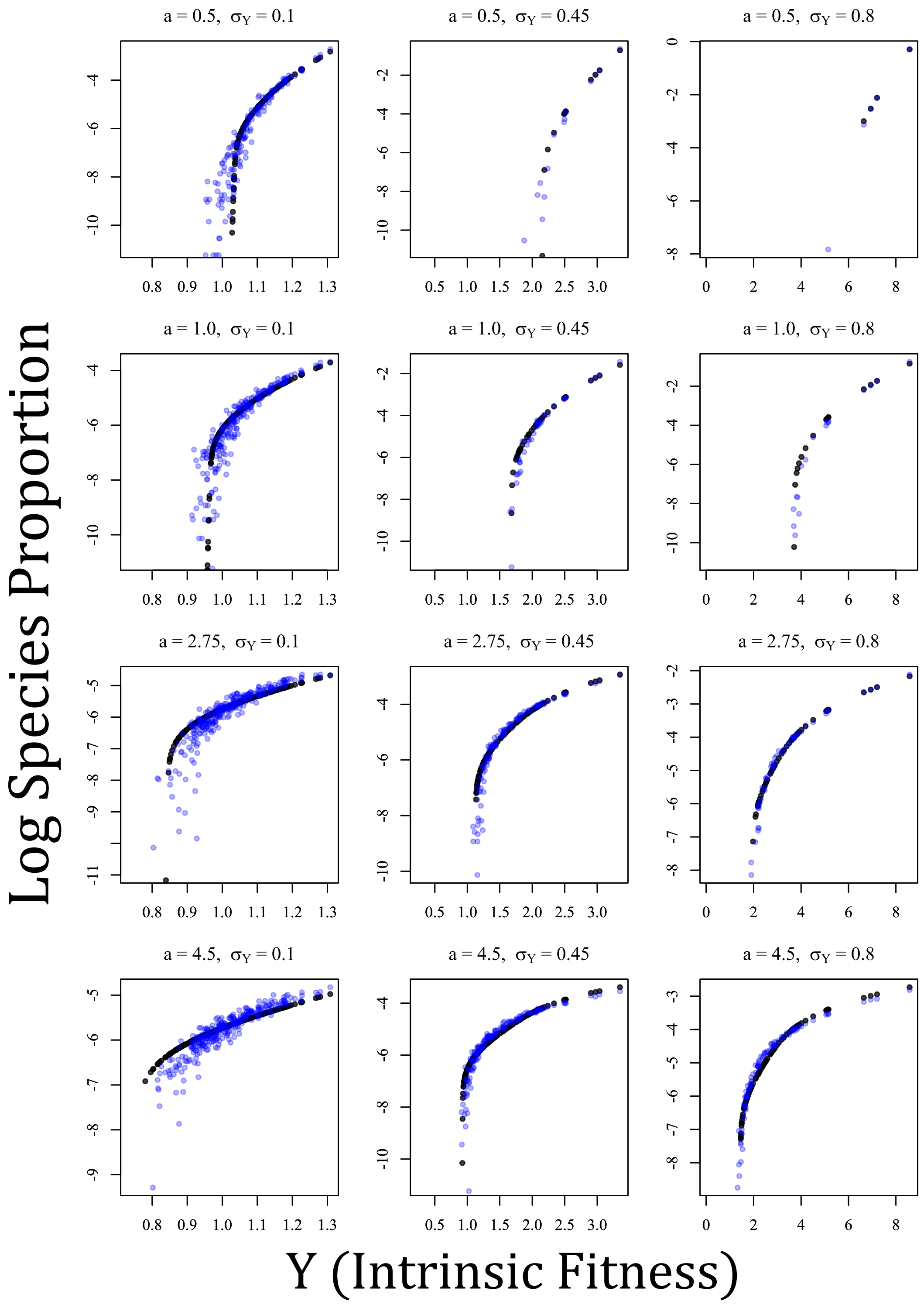

Fig. C3 Comparisons between identical parameterizations of the ODE approximation (black) and SEM (blue) outputs under twelve parameter values when species vary in intrinsic fitness (Y). The y-axis depicts the log-proportion of each species and the x-axis depicts Y of each species. In all plots, $v = 5$, $g = 0.2$, and $D = 1.0$. Other relevant parameters are listed on each plot.

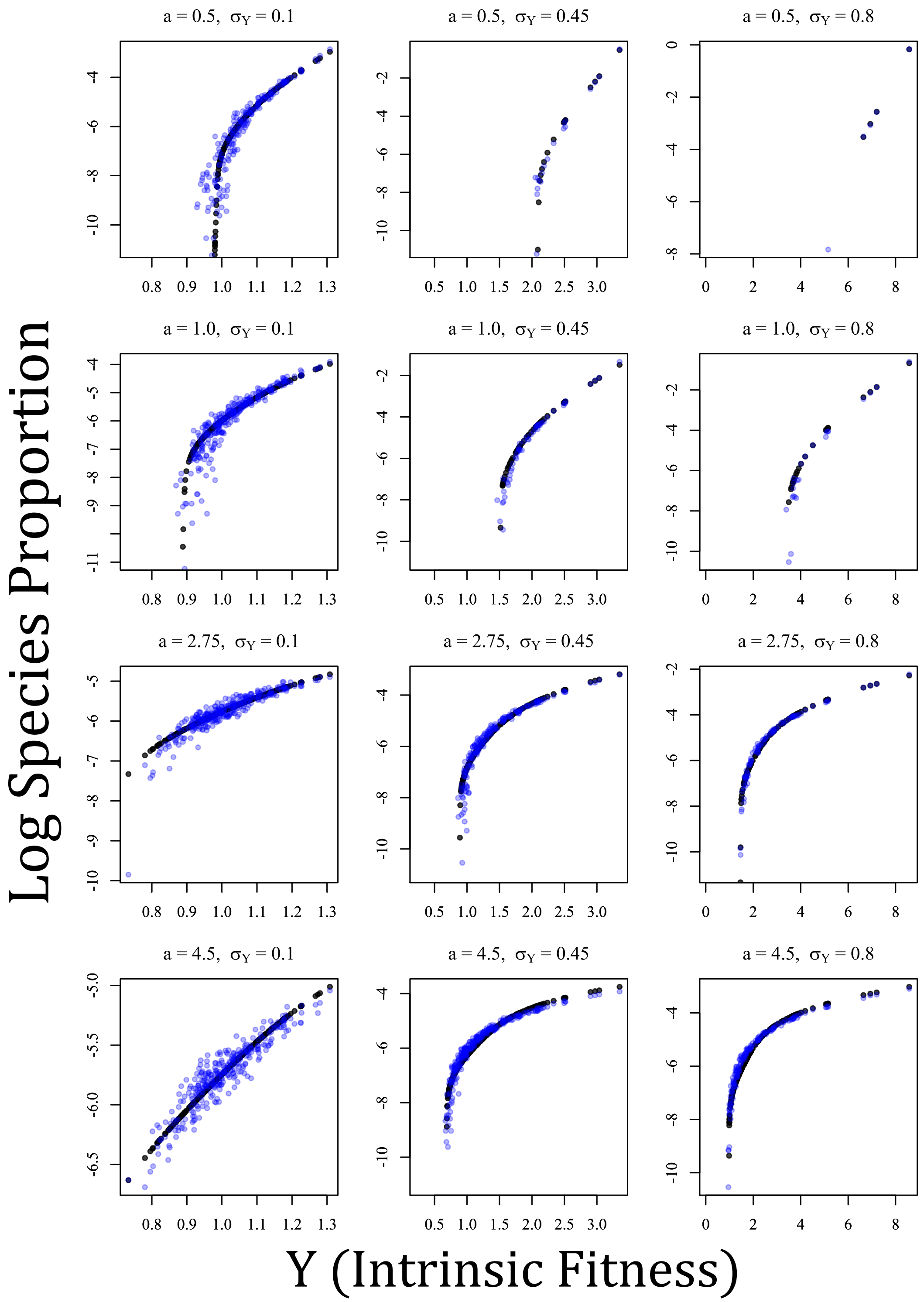

Fig. C4 The same format as Fig. C3, but with $v = 7.5$.

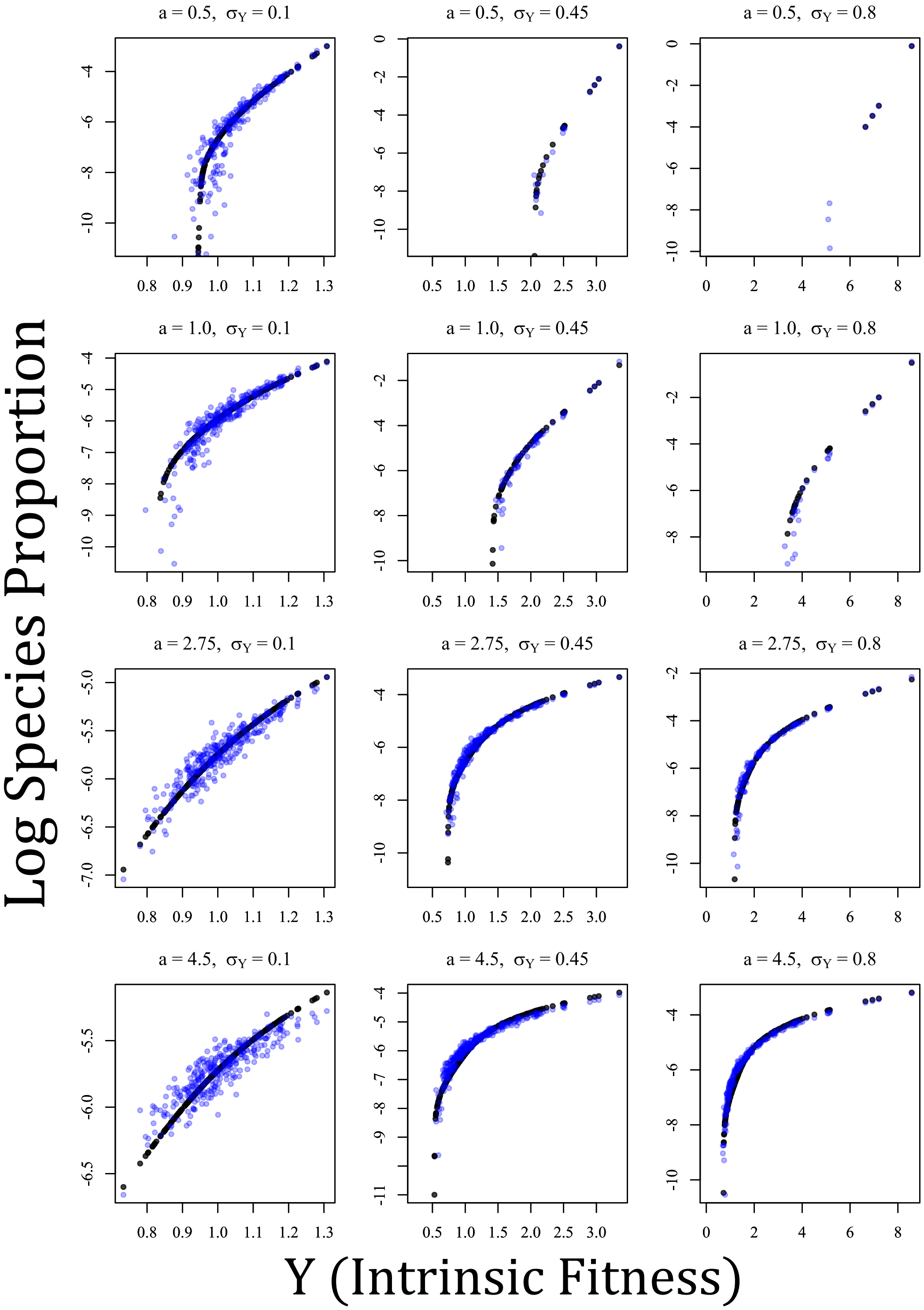

Fig. C5 The same format as Fig. C3, but with $v = 10$.

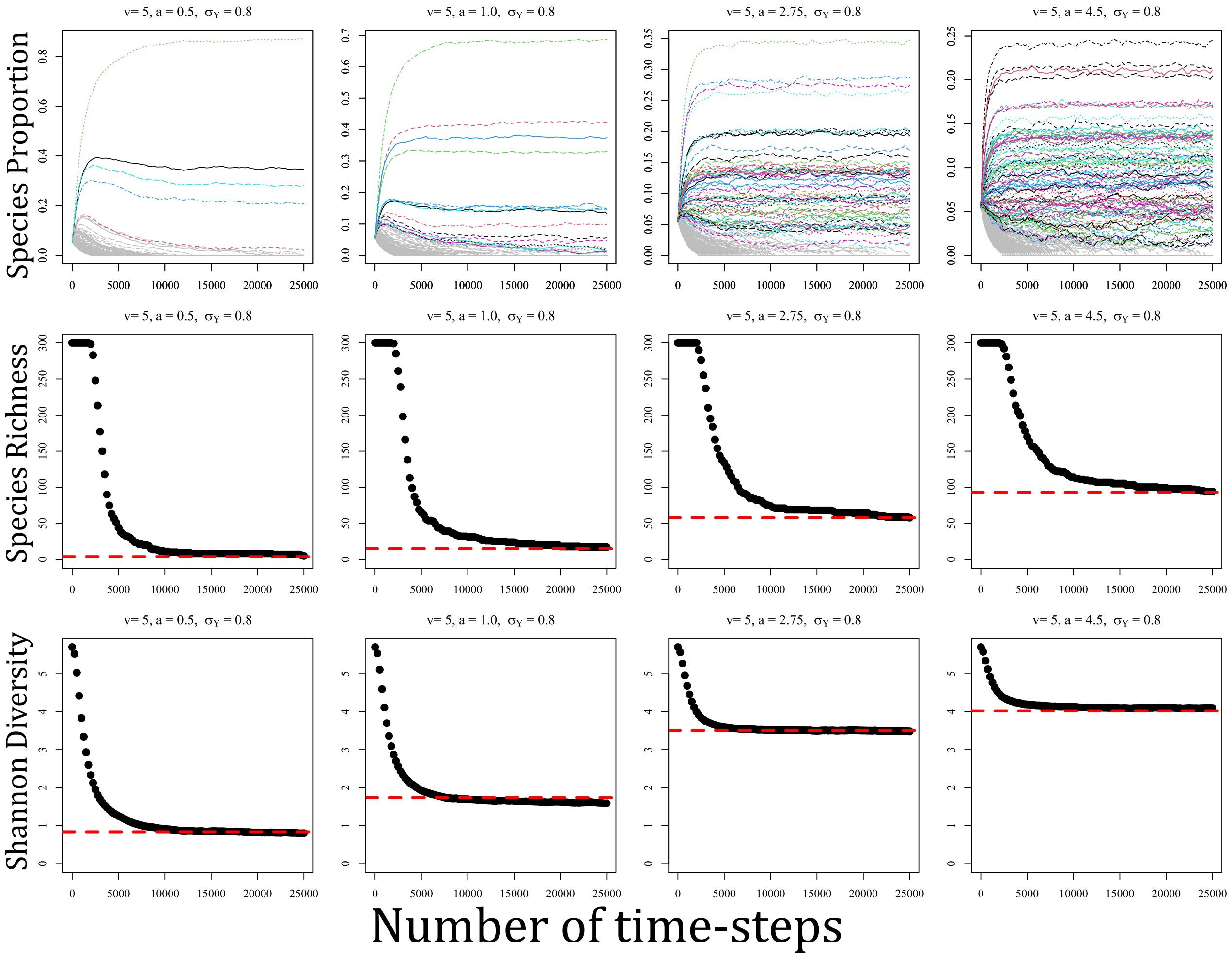

Fig. C6 Examples of the SEM simulation time series outputs, species richness over time in the simulations, and Shannon diversity over time in the simulations. The top row shows examples of the time series outputs of the SEMs. The x-axis is time and the y-axis is each species' proportion. Proportions have been square-root transformed to aid visualization. Colored trajectories indicate species that persisted throughout the simulation; grey trajectories indicate species that went extinct. Parameters are listed on each plot. Dynamics as shown are typical examples from the SEMs. Most species settle into a relatively stable pseudo-equilibrium, while lower abundance species fluctuate due to drift. The second row shows the number of persisting species in the community as a function of time. Each panel corresponds to the plot above it. Most species that go extinct do so in the early stages of the dynamics. Therefore, the vast majority of persisting species likely persist deterministically. The dashed red line is the diversity maintained by the ODE under the same parameterization. All SEMs saturate, approximately, to the dashed line. The third row is the same as the second row, except it shows Shannon diversity instead of species richness.

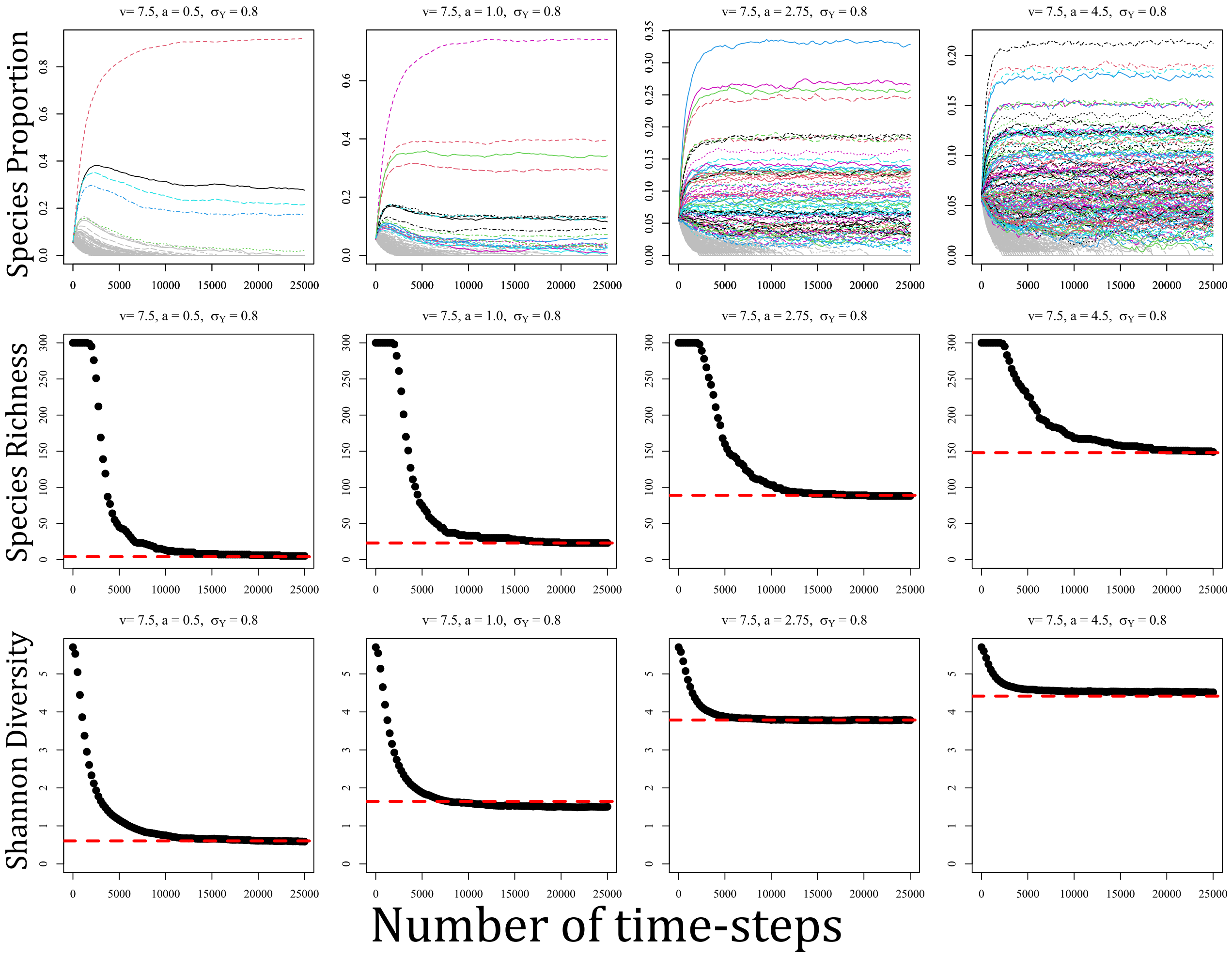

Fig. C7 The same as Fig. C6, but with $v = 7.5$.

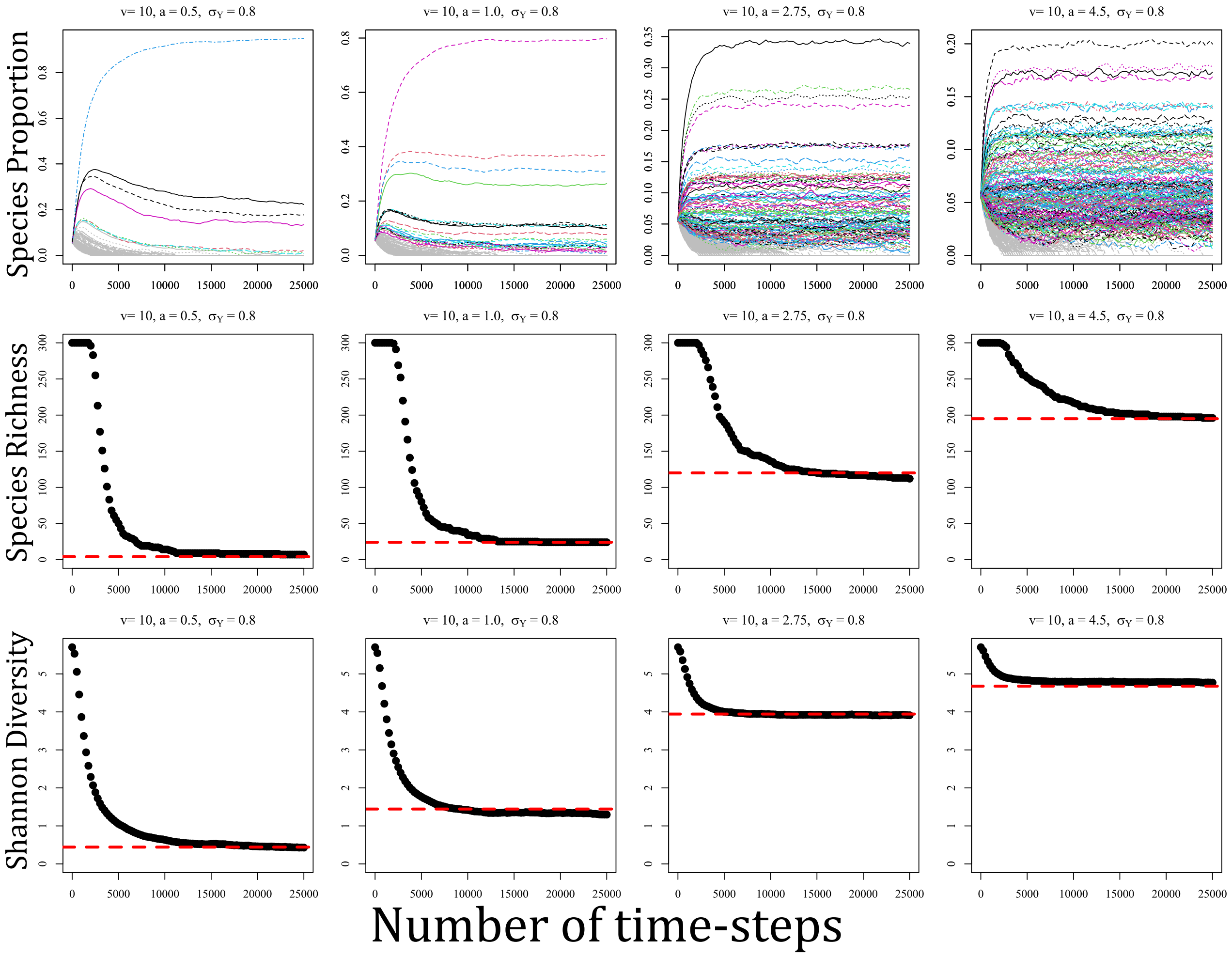

Fig. C8 The same as Fig. C6, but with $v = 10$.
