# Supplementary material for: The functional form of specialised predation affects whether Janzen–Connell effects can prevent competitive exclusion
Source: Ecol Lett. 2022 Apr 26;25(6):1458–70. doi: 10.1111/ele.14014 (PMC9324109; doi:10.1111/ele.14014)
Supplement: Supplementary file 4 — Supplementary Material [file ELE-25-1458-s007.pdf]

Appendix D: Additive−Distance-decay model SEM and ODE approximation

Contents

1 Introduction **2**

2 Spatially Explicit Model and derivation of ODE approximation **2**

2.1 Spatially Explicit Model 2

2.2 ODE Model 4

3 Comparison between ODE model and SEM model **8**

3.1 ODE and SEM parameterization 9

3.2 Results of comparison 9

4 Derivation of invasion criteria **10**

5 Note on ODE approximation **13**

6 Figures **15**

1 Introduction

In this Appendix, I analyze the additive−distance-decay (AD) model presented in the main text. This Appendix is composed of three main sections: **(1)** I introduce a spatially explicit model (SEM) for the AD model. I then demonstrate that taking the expected offspring abundances on each patch yields the Ordinary Differential Equation (ODE) model discussed in the main text. **(2)** I provide outputs of the ODE model and the SEM model under the same parameterizations. I show the outputs are very similar, hence demonstrating that the ODE is a sufficiently accurate approximation of the SEM. **(3)** I provide the derivation of the approximate invasion criteria for the AD model (Table 1 in the main text). The structure of this appendix is identical to that of appendices A-C.

2 Spatially Explicit Model and derivation of ODE approximation

In this section, I discuss the SEM, briefly reviewing within-patch dynamics from the main text. Then, I show the derivation of the ODE approximation. I assume the reader is generally familiar with the model discussed in the main text.

2.1 Spatially Explicit Model

I developed a Spatially Explicit Model (SEM) that incorporates additive predation pressure that decays with distance. The model consists of a community on a grid of $L \times L$ patches (M total patches, $M = L^2$) modeled as a torus to avoid edge effects. A single tree is present on every patch on the grid. At each time step, each tree dies with probability δ and tree replacement occur via a lottery based on seedling abundances on each patch. I assume predation pressure increases linearly as a function of conspecific density and that predation induced by conspecific adults on a patch decreases exponentially with distance. As noted in the main text, offspring

abundances are defined by

$$
\begin{aligned}
S_{i,i}(x) &= Y_i\big[(1-D)+p_iD\big]J_{i,i}(x)\\
S_{i,k}(x) &= Y_ip_iDJ_{i,k}(x)\\
S_{all,i}(x) &= \sum_{n=1}^{N} S_{n,i,x}
\end{aligned}
\tag{D.1}
$$

where $S_{A,B}(x)$ is the offspring abundance of species A on a patch occupied by species B at location x, $J_{i,i}(x)$ and $J_{i,k}(x)$ are how JCEs affect offspring surivorship, p_i is the proportion of species i in the population, Y_i is the intrinsic fitness of species i, and D is the dispersal proportion. For the AD model:

$$
\begin{aligned}
J_{i,i}(x) &= \exp\Bigg[-a\Bigg(1+\sum_{m=1}^{Mp_i} e^{-x_{i,m}/v}\Bigg)\Bigg]\\
J_{i,k}(x) &= \exp\Bigg[-a\sum_{m=1}^{Mp_i} e^{-x_{i,m}/v}\Bigg]
\end{aligned}
\tag{D.2}
$$

Mp_i is the total number of individuals of species i in the community and $x_{i,m}$ is the distance between the m_{th} closest individual of species i and the focal patch at location x. $J_{i,i}(x)$ contains the $+1$ term because the focal patch contains an individual of species i. v defines rate at which predataion declines with distance (higher v indicates a lower rate of predation decay) and a is the baseline rate of predation pressure. Offspring abundances are then determined by the following equations:

$$S_{i,i}(x) = Y_i\big[(1-D)+p_iD\big]\ \exp\Bigg[-a\bigg(1+\sum_{m=1}^{Mp_i} e^{-x_{i,m}/v}\bigg)\Bigg]$$

$$S_{i,k}(x) = Y_ip_iD\ \exp\Bigg[-a\sum_{m=1}^{Mp_i} e^{-x_{i,m}/v}\Bigg]$$

$$S_{all,i}(x) = Y_i\big[(1-D)+p_iD\big]\ \exp\Bigg[-a\bigg(1+\sum_{m=1}^{Mp_i} e^{-x_{i,m}/v}\bigg)\Bigg] + D\sum_{k\neq i} Y_kp_k\ \exp\Bigg[-a\sum_{m=1}^{Mp_k} e^{-x_{k,m}/v}\Bigg] \tag{D.3}$$

As noted before, replacements are determined by a lottery model. The lottery is determined by the abundance of offspring. Let $P_{A,B}(x)$ be the probability species A colonizes a patch previously occupied by species B at location x. Then, $P_{i,i}(x) = S_{i,i}(x)/S_{all,i}(x)$ and $P_{i,k}(x) = S_{i,k}(x)/S_{all,k}(x)$. For the SEM, patch-specific JCEs are determined by the euclidean distances of adults surrounding each patch. The position of each patch is defined by its center point and it is assumed offspring are at the center of each patch.

2.2 ODE Model

To derive the ODE model, I take approximations of the expected values of $P_{i,i}(x)$ and $P_{i,k}(x)$. To do so, I take the expected abundance of $S_{i,i}(x)$, $S_{i,k}(x)$, and $S_{all,i}(x)$ and then take their quotients. Expectations are taken with respect to space. Using this, I derive the the ODE approximation

$$\frac{dp_i}{dt} = \delta\Bigg[\frac{\mathbb{E}[S_{i,i}(x)]}{\mathbb{E}[S_{all,i}(x)]}p_i + \sum_{k\neq i}\frac{\mathbb{E}[S_{i,k}]}{\mathbb{E}[S_{all,k}(x)]}p_k - p_i\Bigg] \tag{D.4}$$

that captures the behavior of the SEM. See "Note" at the end of this Appendix for additional information about the assumptions of this approximation.

Predation pressure in the SEM is implemented using the proximity trees in space that, computationally, is stored in a matrix. The deterministic ODE model is spatially implicit. Therefore, it is necessary to use an approximation of the terms that does not require spatial information.

Specifically, it is necessary to approximate $J_{i,i}(x)$ and $J_{i,k}(x)$ in spatially implicit terms. To do this, I evaluate

$$\mathbb{E}\left[J_{i,k}(x)\right] \tag{D.5}$$

noting that $J_{i,i}(x) = e^{-a}J_{i,k}(x)$ (in which case, it is sufficient to just compute $J_{i,k}(x)$).

To accomplish this, I evaluate the total mortality an individual of species i induces given the AD model. Then, I assume individuals (and thus, mortality induced) are randomly distributed in space. Then, I calculate the probability that mortality does not occur on a random patch. The total mortality induced by an individual of species i is equal to

$$\lambda = 2\pi g \int_0^\infty x\left(1 - e^{-ae^{-x/v}}\right)\mathrm{d}x \tag{D.6}$$

where x represents distance and $\left(1 - e^{-ae^{-x/v}}\right)$ is the proportion of offspring that do not survive x meters away from an adult of species i. The 2π term reflects that predation pressure occurs in 2D space. Therefore, λ then represents the sum of mortality induced by an individual of species i.

Species i is of proportion p_i in the population and I assume adults of species i are approximately randomly distributed in space. Then adults of species i are Poission distributed in 2D space and, by extension, mortality is also approximately Poisson distributed with rate parameter $p_i\lambda$. Then, the probability that no mortality event occurs on a random patch (i.e. the probability of survival) is relatively easy to define:

$$\mathrm{P(no\ mortality)} = \mathrm{P}(\mathrm{Poi}(p_i\lambda)\ = 0)\ = \frac{1^0 e^{-p_i\lambda}}{0!} = e^{-p_i\lambda}.$$

This yields:

$$\mathbb{E}[J_{i,k}(x)] = \exp\left[-p_i\ 2\pi g \int_0^\infty x\left(1 - e^{-ae^{-x/v}}\right)\mathrm{d}x\right]. \tag{D.7}$$

which is the quantity of interested (the probability of offspring survival). It is necessary to evaluate this quantity.

The integral (λ) is not trivial to evaluate. One approach is to take the Taylor series of $e^{-ae^{-x/v}}$. The expression becomes

$$2\pi g \int_0^\infty x\left(1 - \sum_{n=0}^{\infty} \frac{(-ae^{-x/v})^n}{n!}\right)\mathrm{d}x \tag{D.8}$$

When $n = 1$, $\frac{(-ae^{-x/v})^n}{n!} = 1$. Therefore, the above can be rewritten as

$$2\pi g \int_0^\infty x\left(1 - \left[1 + \sum_{n=1}^{\infty} \frac{(-ae^{-x/v})^n}{n!}\right]\right) = -2\pi g \int_0^\infty x \sum_{n=1}^{\infty} \frac{(-ae^{-x/v})^n}{n!}\mathrm{d}x \tag{D.9}$$

noting the change in the index of summation ($n = 1$). Then, changing the order of integration and summation and evaluating the integral yields

$$-2\pi g \sum_{n=1}^{\infty} \frac{v(nx+v)\left(-ae^{-x/v}\right)^n}{n^2 n!}\Bigg|_0^\infty \tag{D.10}$$

When all the constants are positive (as is the case for this expression) it is easy to show that

$$\lim_{x\to\infty} \frac{v(nx+v)\left(-ae^{-x/v}\right)^n}{n^2 n!} \to 0 \tag{D.11}$$

for all $n \geq 1$ on the basis that $\lim_{x\to\infty}(x + K_1)e^{-xK_2} \to 0$ if K_1 and K_2 are positive constants

(which is the case for the equivalent terms involving v and n in the equation above). Therefore,

$$-2\pi g \sum_{n=1}^{\infty} \frac{v(nx+v)\left(-ae^{-x/v}\right)^n}{n^2 n!}\Bigg|_0^{\infty} =$$
$$\lim_{x\to\infty}\Bigg(-2\pi g \sum_{n=1}^{\infty} \frac{v(nx+v)\left(-ae^{-x/v}\right)^n}{n^2 n!}\Bigg) - \lim_{x\to 0}\Bigg(-2\pi \sum_{n=1}^{\infty} \frac{v(nx+v)\left(-ae^{-x/v}\right)^n}{n^2 n!}\Bigg)$$
$$= 2\pi g v^2 a \sum_{n=1}^{\infty} \frac{(-a)^{n-1}}{n^2 n!} \tag{D.12}$$

after some small algebraic manipulations. Importantly, while the summation

$$\sum_{n=1}^{\infty} \frac{(-a)^{n-1}}{n^2 n!}$$

has no elementary solution I am aware of, it has all the properties of a Generalized Hypergeometric Function (henceforth, GHF). To see this, note that the summation follows the form

$$\sum_{n=1}^{\infty} \frac{(-a)^{n-1}}{n^2 n!} = 1 + \frac{1}{2^2 2!}(-a)^1 + \frac{1}{3^2 3!}(-a)^2 + ... + \frac{1}{n^2 n!}(-a)^{n-1} \tag{D.13}$$

Let β_n be the n_{th} coefficient of the summation (i.e. $\beta_0 = 1$, $\beta_1 = \frac{1}{2^2 2!}$, etc.). Therefore,

$$\frac{\beta_{n+1}}{\beta_n} = \frac{(n+2)^2(n+2)!}{(n+1)^2(n+1)!} = \frac{(n+2)^3}{(n+1)^2} \tag{D.14}$$

This yields the GHF

$$\sum_{n=1}^{\infty} \frac{(-a)^{n-1}}{n^2 n!} = {}_3F_3(1,1,1;2,2,2;-a) \tag{D.15}$$

(a result the interested reader can confirm using Wolfram Mathematica; (Wolfram Inc., 2021)).

For simplicity, I use the notation

$$H(a) = {}_3F_3(1,1,1;2,2,2;-a) \tag{D.16}$$

from this point forward. Therefore, finally,

$$2\pi g \int_0^\infty x\left(1 - e^{-ae^{-x/v}}\right)\mathrm{d}x = 2a\pi g v^2 H(a) \tag{D.17}$$

in which case,

$$\mathbb{E}[J_{i,k}(x)] \approx e^{-2ap_i \pi g v^2 H(a)} \tag{D.18}$$

Substituting this into the offspring abundance equations

$$\begin{aligned} \mathbb{E}[S_{i,k}(x)] &= Y_i\big[(1-D) + p_i D\big]e^{-a}e^{-ap_i E_D H(a)} \\ \mathbb{E}[S_{i,k}(x)] &= Y_i p_i D e^{-ap_i E_D H(a)} \end{aligned} \tag{D.19}$$

where

$$E_D = 2\pi g v^2$$

and where $S_{all,i}(x) = \sum_{n=1}^{N} S_{n,i}(x)$. These expressions are identical to the AD model offspring abundance equations in the main text.

3 Comparison between ODE model and SEM model

In this section, I describe simulations that compare the ODE model to the SEM. I demonstrate that the SEM and ODE model yield highly similar outputs of species abundance and species richness. I provide 36 comparisons of the SEM to the ODE (12 cases in which $v = 5$, 12 cases in which $v = 7.5$, and 12 cases in which $v = 10$).

3.1 ODE and SEM parameterization

Each SEM simulation began with 300 species at equal abundance, with individuals randomly distributed throughout the community. Simulations were conducted on a 275×275 torus (thus containing 275^2 individual trees). I used the following parameters: $Y \sim \text{lognormal}[\mu = 0, \sigma_Y]$ with $\sigma_Y \sim \{0.1, 0.45, 0.8\}$ and $a \sim \{0.5, 1.0, 2.75, 4.5\}$. In all simulations, $g = 0.20$ and $D = 1$. I tested each of the 12 parameter combinations of σ_Y and a with $v \sim \{5, 7.5, 10\}$. This generated 36 outputs. Simulations were run for about 65 generations, sufficient time for the community to approximately reach equilibrium without drift dominating the dynamics of the lower abundance species. See Figs. D6-D8 for typical outputs of the SEM time series dynamics.

A corresponding set of 36 ODE simulations were run using the same parameterizations as the SEM. I compared the outputs of the SEM and ODE model in terms of species diversity, species abundance, and Shannon diversity. I considered a species to be extinct if it had less than 1 individual at any point of the simulation. This was implemented directly in the SEM; for the ODE model, I assumed a species, i, to be extinct if $p_i^* < 1/275^2$ where p_i^* is the equilibrium proportion of species i. Note that these simulations do not attempt to demonstrate the long-term resistance against extinction due to drift. Rather, they demonstrate that the ODE model and SEM yield similar outputs of expected species abundance and richness given the same parameterization.

3.2 Results of comparison

ODE model and SEM produced very similar species richness and Shannon diversity (Figs. D1, D2). The ODE model and SEM also produced very similar species proportions (Figs. D3-D5). To quantify the quality of the approximation, I calculated the mean difference in species richness between the ODE model and SEM, ΔR:

$$\Delta R = \frac{1}{S} \sum_{k=1}^{S} (R^k_{\text{SEM}} - R^k_{\text{ODE}}) \tag{D.20}$$

where S is the number of simulations, and R^k_{SEM} and R^k_{ODE} are the species richness of the k_{th} simulation of the SEM and ODE model, respectively. I also examined the r^2 (coefficient of determination) between SEM and ODE species richness. For the comparisons, $\Delta R = -.52$ and $r^2 = 0.99$. Overall, the ODE provides a highly similar, albeit non-exact, estimation of species diversity. Error in which the ODE model predicted greater diversity than the SEM is most likely due to stochastic extinction due to drift. This is particularly likely when species richness is high, where the expected abundance of each species is correspondingly smaller. Cases in which the ODE model predicted lower species richness are likely due to incomplete transient dynamics of the SEM.

4 Derivation of invasion criteria

In this section, I derive the approximate invasion criteria of the additive−fixed-distance model when species experience inter-specific variation in intrinsic (Y) and $D = 1$. Recalling the derivations above,

$$\mathbb{E}[J_{k,i}(x)] = e^{-ap_i E_D H(a)} \tag{D.21}$$

and

$$\mathbb{E}[J_{i,i}(x)] = e^{-a}e^{-ap_i E_D H(a)} \tag{D.22}$$

The invasion criteria of an invader can be expressed as when the per capita growth rate as $p_i \to 0$. Using variables previous defined in this Appendix, the per capita growth rate of species i (substituting in the seedling abundance values) is

$$\begin{aligned}\frac{1}{p_i}\frac{dp_i}{dt} = r_i = \delta\Bigg[&\frac{Y_i\big[(1-D)+p_iD\big]}{Y_i\big[(1-D)+p_iD\big]\,\mathbb{E}[J_{i,i}(x)] + \sum_{k\neq i} Y_k p_k D\,\mathbb{E}[J_{k,i}(x)]} \\ &+ Y_iD\sum_{m\neq i}\frac{1}{Y_m\big[(1-D)+p_mD\big]\,\mathbb{E}[J_{m,m}(x)] + \sum_{k\neq m} Y_k p_k D\,\mathbb{E}[J_{k,m}(x)]}p_m - 1\Bigg]\end{aligned} \tag{D.23}$$

Species i can invade is this quantity if positive when it is rare ($p_i \to 0$). When $D = 1$ (the case of interest), the above reduces to

$$Y_i \sum_{m \neq i} \frac{p_m}{Y_m \, \mathbb{E}[J_{m,m}(x)] p_m + \sum_{k \neq m} Y_k p_k \, \mathbb{E}[J_{k,m}(x)]} > 1 \tag{D.24}$$

To simplify the above equation, I ignore the term $Y_m \, \mathbb{E}[J_{m,m}(x)] p_m$ in the denominator and incorporate an additional term representing species m into the summation, yielding:

$$Y_i \sum_{m \neq i} \frac{p_m}{\sum_{k \neq i} Y_k p_k \, \mathbb{E}[J_{k,i}(x)]} > 1 \tag{D.25}$$

This simplification is equivalent to making the species identity of the tree previously occupying a patch (the tree that dies) irrelevant (i.e., JCEs only result from trees nearby the patch rather than the previous occupant of the patch). As long as JCEs occur over a non-trivially small area (such that v is not very small) this assumption does not meaningfully affect the invasion criteria.

Importantly, the denominator of equation (D.25) is no longer directly dependent on m. That is, equation (D.25) can be rewritten as

$$Y_i \left(\sum_{m \neq i} p_m \right) \left(\frac{1}{\sum_{k \neq i} Y_k p_k \, \mathbb{E}[J_{k,i}(x)]} \right) > 1 \tag{D.26}$$

Because, by definition, $\sum_{m \neq i} p_m = 1$, the above equation can be rewritten as

$$Y_i > \sum_{k \neq i} Y_k p_k \, \mathbb{E}[J_{k,i}(x)] \tag{D.27}$$

Substituting the appropriate value for $\mathbb{E}[J_{k,i}(x)]$, the invasion criteria becomes

$$Y_i > \sum_{k \neq i} Y_k p_k e^{-a p_k E_D H(a)} \tag{D.28}$$

recalling that $E_D = 2\pi v^2 g$. I take the linearization of $p_k e^{-ap_k E_D H(a)}$ about the point $1/N$ where N is the number of species in the resident community (and thus, $1/N$ is the average abundance). This yields a close approximation of the expression so long as no species exhibits an abundance much greater than the mean abundance. For simplicity, let

$$J = aE_D H(a) \tag{D.29}$$

The, taking the linearization yields

$$p_k e^{-Jp_k} \approx \frac{e^{-\frac{J}{N}}}{N} + \frac{e^{-\frac{J}{N}}\left(p_k - \frac{1}{N}\right)}{N}\left(1 - \frac{J}{N}\right) \tag{D.30}$$

Substituting this into the original expression, the summation can be rearranged and broken up into three parts:

$$e^{-\frac{J}{N}}\frac{1}{N}\sum_{k\neq i} Y_k + e^{-\frac{J}{N}}\left(1 - \frac{J}{N}\right)\sum_{k\neq i} Y_k p_k - e^{-\frac{J}{N}}\left(1 - \frac{J}{N}\right)\frac{1}{N}\sum_{k\neq i} Y_k \tag{D.31}$$

The first summation is straightforward to calculate:

$$e^{-\frac{J}{N}}\frac{1}{N}\sum_{k\neq i} Y_k = e^{-\frac{J}{N}}\overline{Y} \tag{D.32}$$

where $\overline{Y}$ is the mean intrinsic fitness of the community.

The second term can be expressed by using the property

$$\frac{1}{N}\sum_{m=1}^{N} A_m B_m = \overline{A} \times \overline{B} + \mathrm{Cov}(A, B) \tag{D.33}$$

I apply this property with respect to p and Y, noting that $\overline{p} = \frac{1}{N}$. Using this property and

substituting A and B with p and Y for the first and second summations yields

$$\begin{aligned}
e^{-\frac{J}{N}}\left(1-\frac{J}{N}\right)\sum_{k\neq i}p_kY_k &= e^{-\frac{J}{N}}\left(1-\frac{J}{N}\right)N\frac{1}{N}\sum_{k\neq i}p_kY_k \\
&= e^{-\frac{J}{N}}\left(1-\frac{J}{N}\right)N\left[\overline{Y}\frac{1}{N}+\mathrm{Cov}(p,Y)\right] \\
&= e^{-\frac{J}{N}}\left(1-\frac{J}{N}\right)\left[\overline{Y}+N\ \mathrm{Cov}(p,Y)\right]
\end{aligned} \tag{D.34}$$

The third summation is easy to calculate:

$$-e^{-\frac{J}{N}}\left(1-\frac{J}{N}\right)\frac{1}{N}\sum_{k\neq i}Y_k = -e^{-\frac{J}{N}}\left(1-\frac{J}{N}\right)\overline{Y} \tag{D.35}$$

Adding all three summations together, the third summation term will cancel with the $\overline{Y}$ term of the second summation. Then, after some rearranging, I substitute $J = aE_DH(a)$ back into the equation. This yields:

$$Y_i > \underbrace{\overline{Y}e^{-aH(a)\frac{E_D}{N}}}_{\substack{\text{mean}\\ \text{JCE-fitness term}}} + \underbrace{N\mathrm{Cov}(p,Y)\left(1-aH(a)\frac{E_D}{N}\right)e^{-aH(a)\frac{E_D}{N}}}_{\text{covariance-JCE term}} \tag{D.36}$$

which is identical to expression for the AD model in Table 1 of the main text.

5 Note on ODE approximation

To derive the ODE model, I took approximations of the expected values of $P_{i,i}(x)$ and $P_{i,k}(x)$. To do so, I took the expected abundance of $S_{i,i}(x)$, $S_{i,k}(x)$, and $S_{all,i}(x)$ with respect to space and then examined their quotients. Note that this assumes $\mathbb{E}[S_{i,k}(x)/S_{all,i}(x)] \approx \mathbb{E}[S_{i,k}(x)]/\mathbb{E}[S_{all,k}(x)]$ (I take the expectation of the numerator and denominator and then take the quotient). Using a Taylor Expansion about the mean,

$$\mathbb{E}\left[\frac{S_{i,k}(x)}{S_{all,i}(x)}\right] \approx \frac{\mathbb{E}[S_{i,k}(x)]}{\mathbb{E}[S_{all,k}(x)]} - \frac{\mathrm{Cov}\big(S_{i,k}(x),S_{all,k}(x)\big)}{\mathbb{E}[S_{all,k}(x)]^2} + \mathrm{Var}\big(S_{all,k}(x)\big)\frac{\mathbb{E}[S_{i,k}(x)]}{\mathbb{E}[S_{all,i}(x)]^3}$$

Because there are many species in the community, $S_{all,i}(x) >> S_{i,k}(x)$. This implies that the covariance term and the term containing $\mathbb{E}[S_{all,i}(x)]^3$ are close to zero. Additionally, $\mathrm{Var}\big(S_{all,i}(x)\big)$ is likely small because it is assumed that dispersal is uniform across the community. Therefore, $\mathbb{E}[S_{i,k}(x)/S_{all,i}(x)] \approx \mathbb{E}[S_{i,k}(x)]/\,\mathbb{E}[S_{all,k}(x)]$ is likely a good approximation. I rely on the quantitative similarity of the SEM and ODE model to validate this assumption.

References

Inc., W.R. (2021). Mathematica, Version 13.0.0. Champaign, IL, 2021.

6 Figures

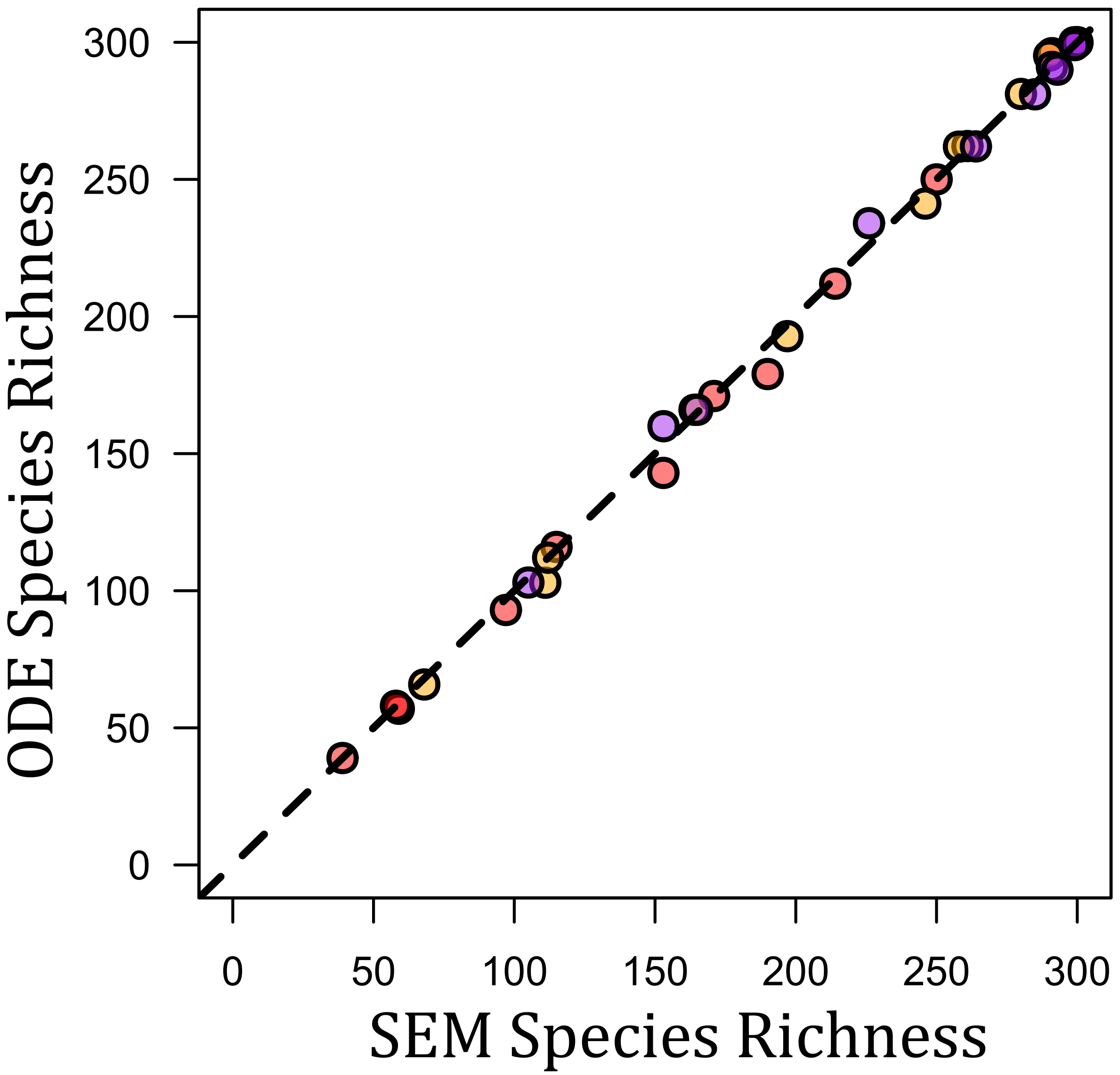

Fig. D1 ODE model validation. The figures compare species richness between SEM and ODE model simulations under identical parameterizations. The dashed line is the one-to-one line (points on the line represent when the SEM and ODE yield the exact same diversity output). Red points are when $v = 5$, orange/yellow points are when $v = 7.5$, and purple points are when $v = 10$. To a first approximation, the ODE model yields the same output as the SEM. These parameter values span the most of the parameter space explored in Figs. 2 and 3 of the main text.

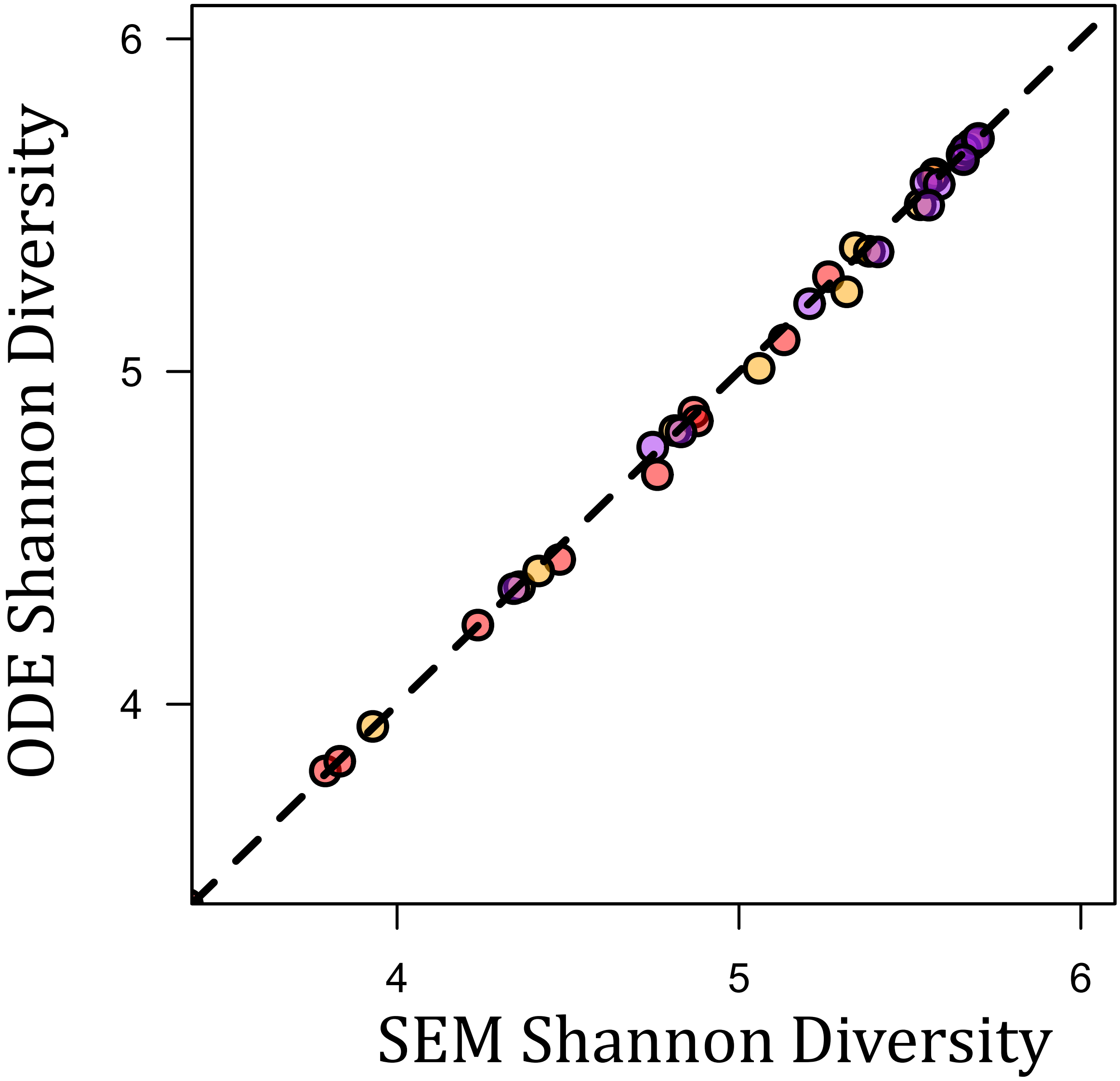

Fig. D2 The same as the Fig. D1, but showing Shannon Diversity instead of species richness. As in the above case, the SEM and ODE model yield very similar outputs.

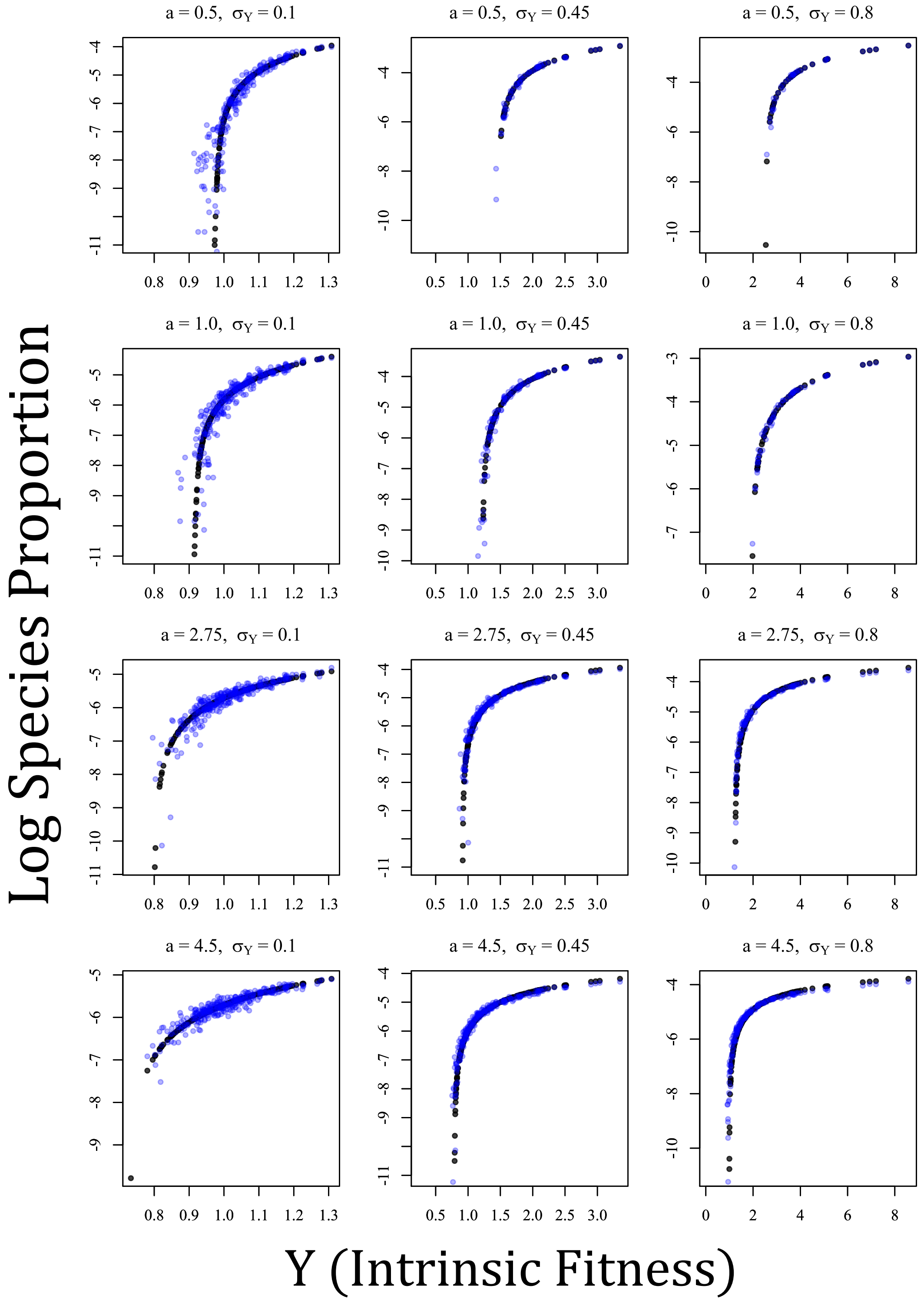

Fig. D3 Comparisons between identical parameterizations of the ODE approximation (black) and SEM (blue) outputs under twelve parameter values when species vary in intrinsic fitness (Y). The y-axis depicts the log-proportion of each species and the x-axis depicts Y of each species. In all plots, $v = 5$, $g = 0.2$, and $D = 1.0$. Other relevant parameters are listed on each plot.

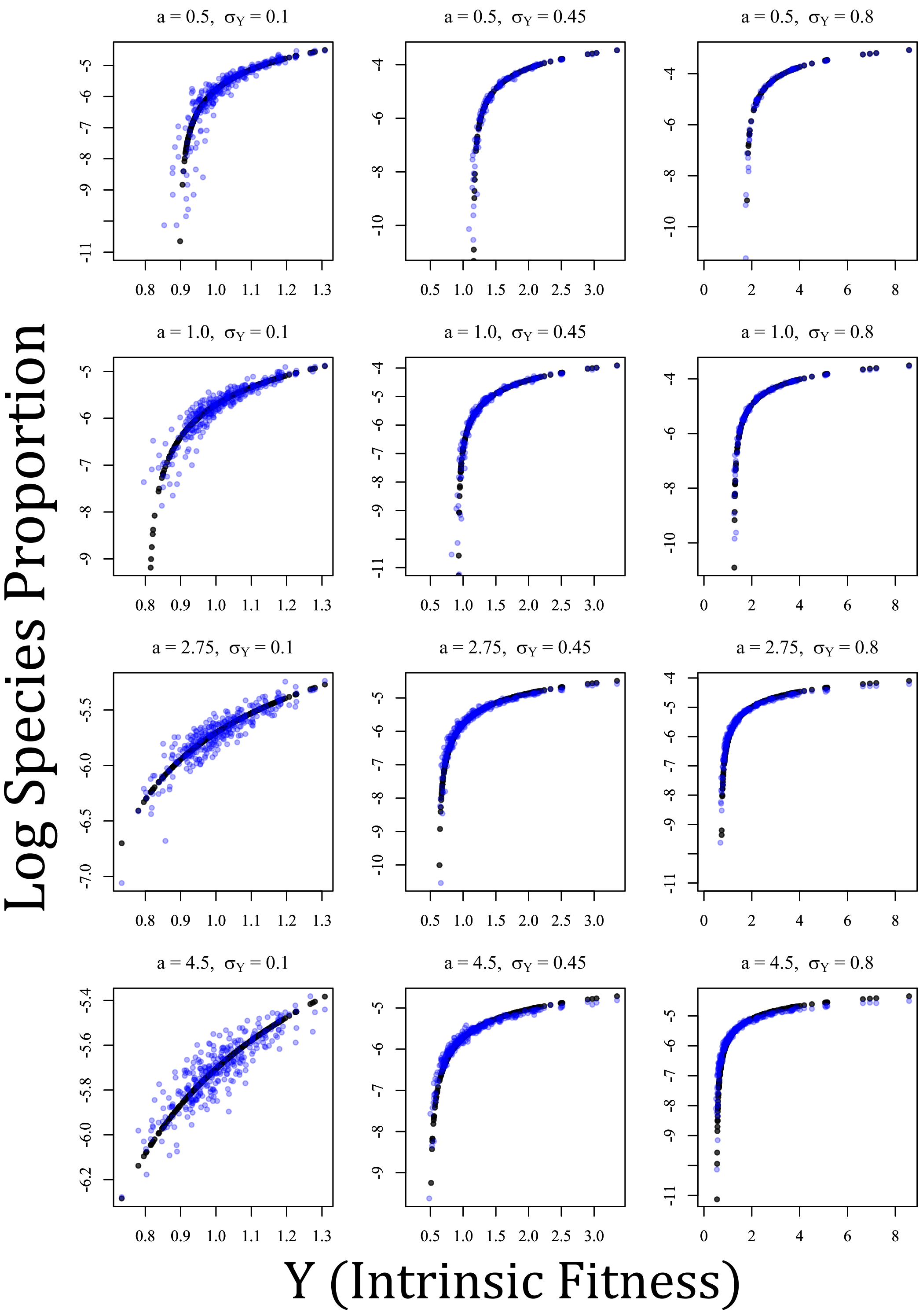

Fig. D4 The same format as Fig. D3, but with $v = 7.5$.

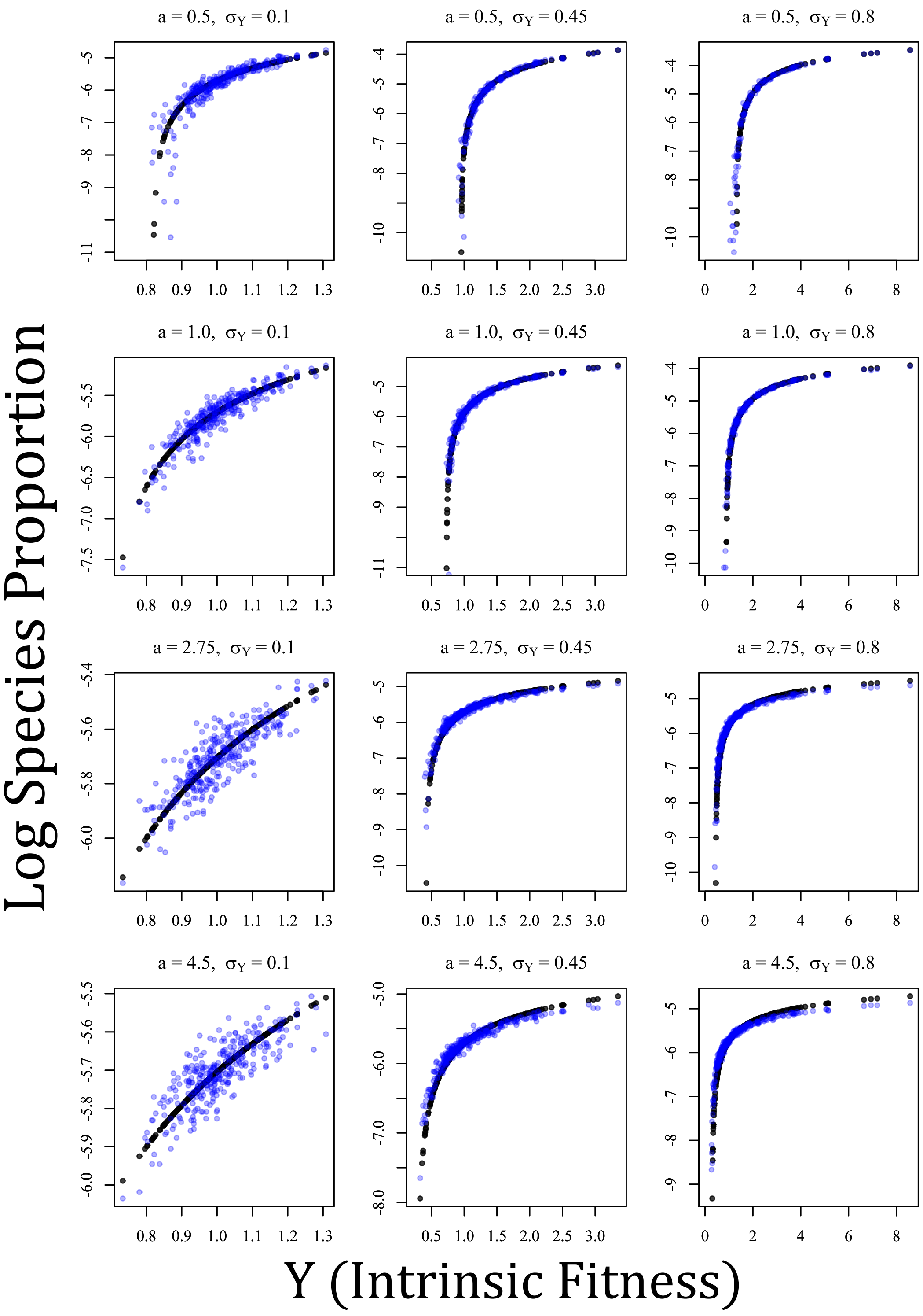

Fig. D5 The same format as Fig. D3, but with $v = 10$.

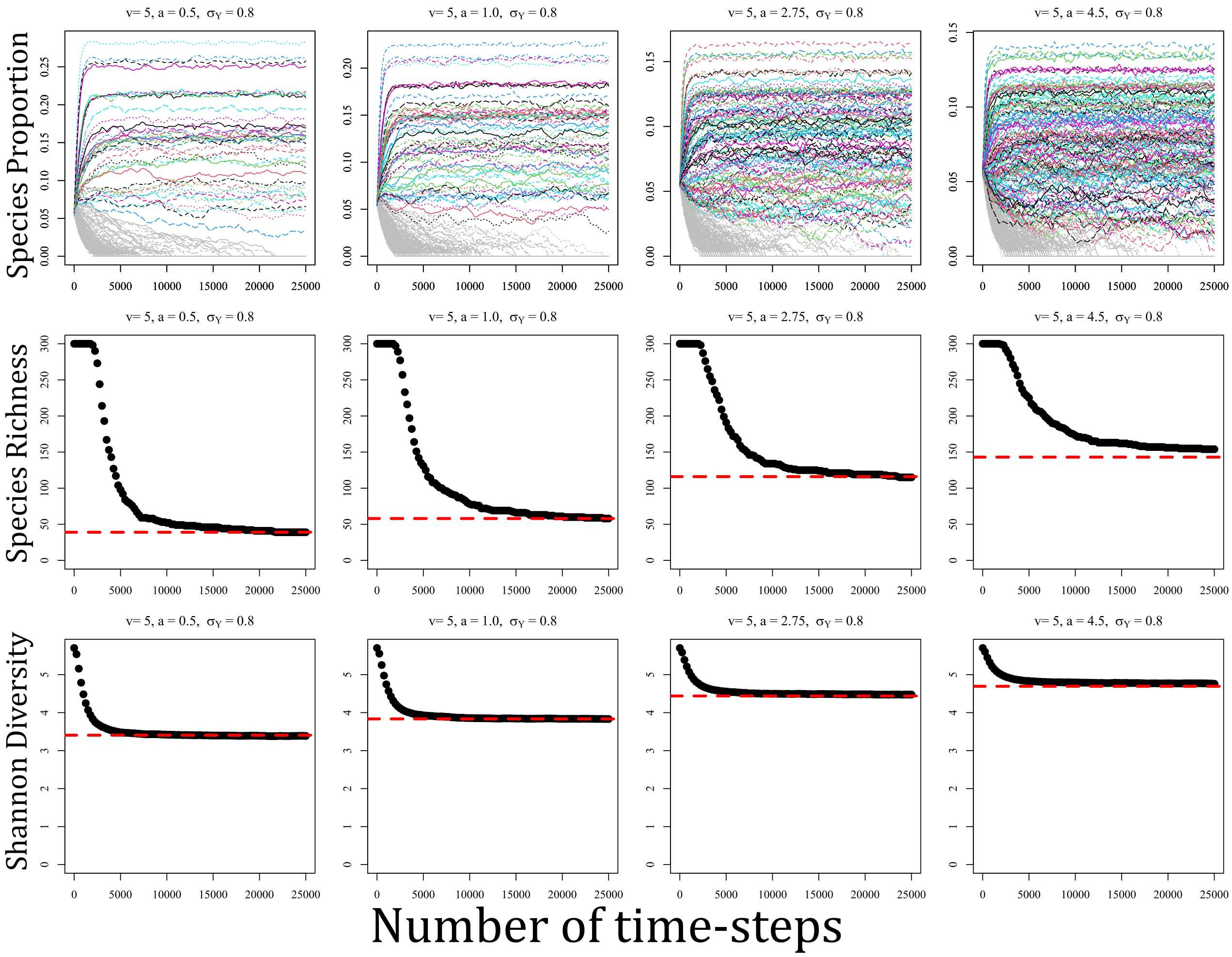

Fig. D6 Examples of the SEM simulation time series outputs, species richness over time in the simulations, and Shannon diversity over time in the simulations. The top row shows examples of the time series outputs of the SEMs. The x-axis is time and the y-axis is each species' proportion. Proportions have been square-root transformed to aid visualization. Colored trajectories indicate species that persisted throughout the simulation; grey trajectories indicate species that went extinct. Parameters are listed on each plot. Dynamics as shown are typical examples from the SEMs. Most species settle into a relatively stable pseudo-equilibrium, while lower abundance species fluctuate due to drift. The second row shows the number of persisting species in the community as a function of time. Each panel corresponds to the plot above it. Most species that go extinct do so in the early stages of the dynamics. Therefore, the vast majority of persisting species likely persist deterministically. The dashed red line is the diversity maintained by the ODE under the same parameterization. All SEMs saturate, approximately, to the dashed line. The third row is the same as the second row, except it shows Shannon diversity instead of species richness.

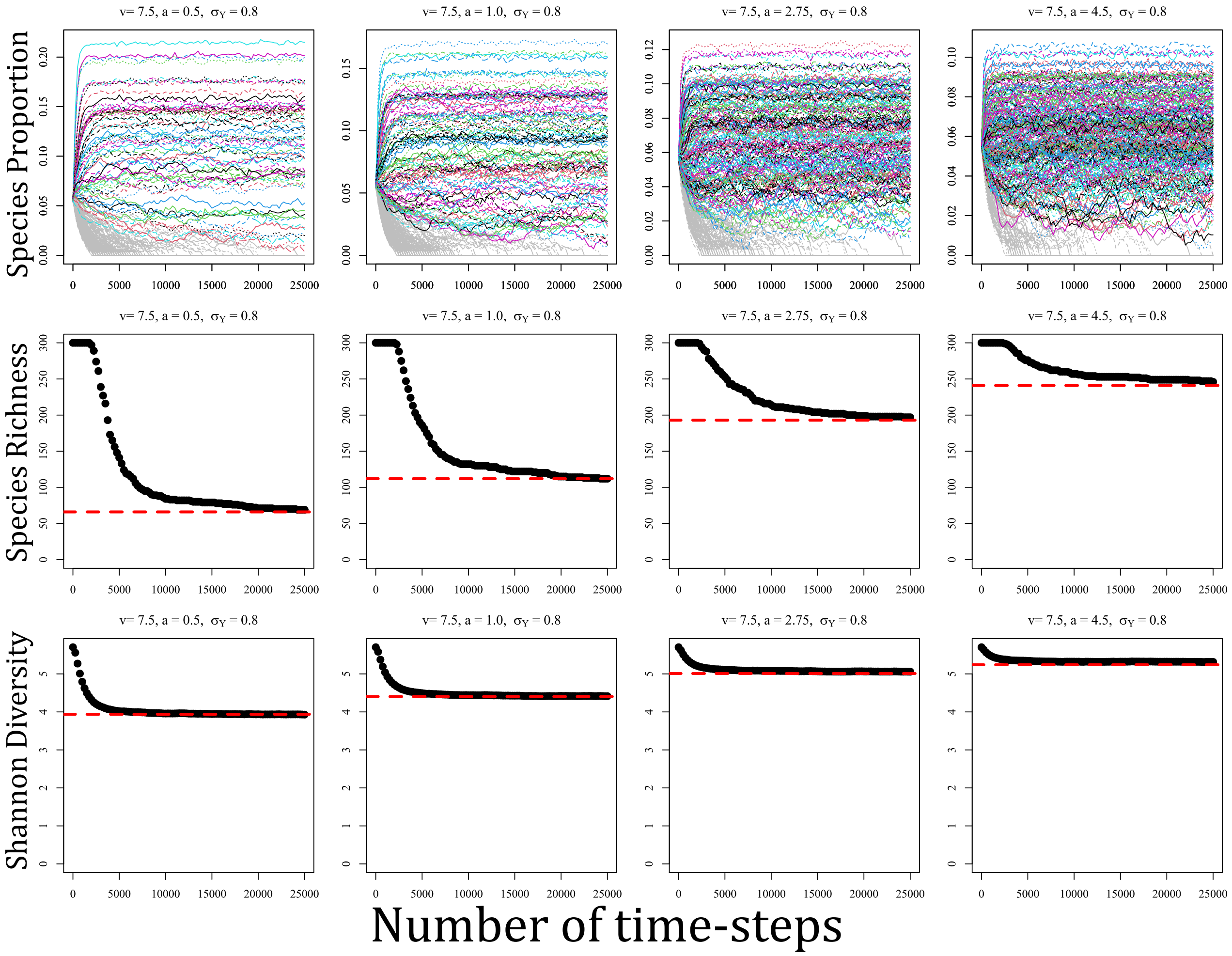

Fig. D7 The same as Fig. D6, but with $v = 7.5$.

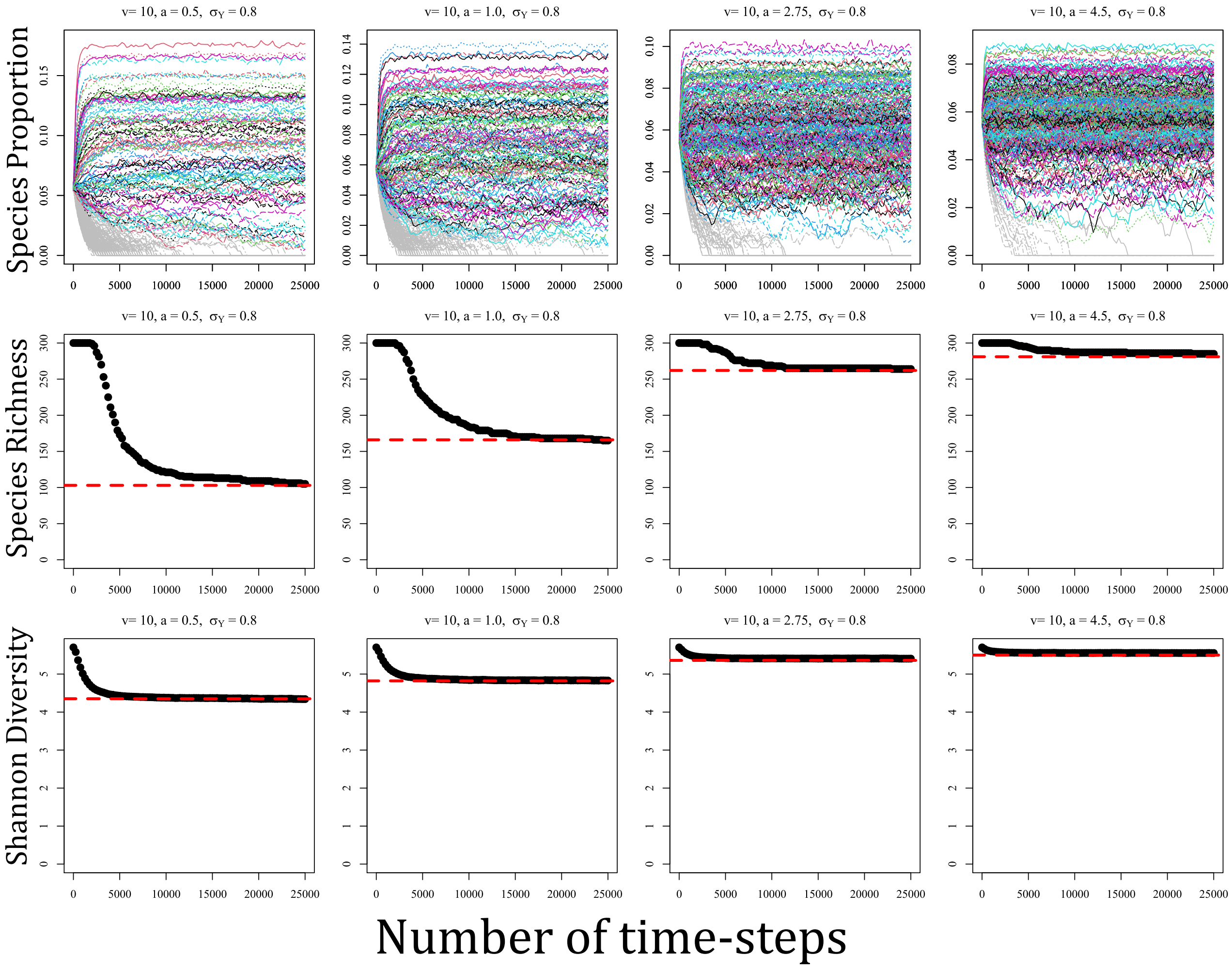

Fig. D8 The same as Fig. D6, but with $v = 10$.
