# Supplementary material for: The functional form of specialised predation affects whether Janzen–Connell effects can prevent competitive exclusion
Source: Ecol Lett. 2022 Apr 26;25(6):1458–70. doi: 10.1111/ele.14014 (PMC9324109; doi:10.1111/ele.14014)
Supplement: Supplementary file 5 — Supplementary Material [file ELE-25-1458-s003.pdf]

Appendix E: Comprehensive explanation of the model formulation and model normalizations

Contents

1 Introduction **2**

2 General modeling framework **2**

3 Normalizations **5**

3.1 Normalization of distance-decay and fixed-distance functional forms 5

3.1.1 Connection to previous literature 6

3.2 Normalization of additive and non-additive functional forms 8

1 Introduction

In this Appendix, I explain the general conceptual and mathematical framework of the model in greater detail than is presented in the main text. Then, I provide detailed mathematical and conceptual explanations for the normalizations introduced in the main text. These normalizations are essential for the modeling comparisons, as they ensure the mean predation pressure offspring experience is identical between models.

2 General modeling framework

In this section, I provide an in-depth explanation of the within-patch dynamics and explicitly articulate how predation is incorporated into the model. Much of this first section is simply a re-iteration of content found in the main text, but in greater detail. The below descriptions are also relevant for motivating the inter-model normalizations.

The tree community model is conceptualized as having discrete time-steps. Each time-step, offspring of each species accumulate on each patch. Offspring abundance are determined in the following way: **(1)** each tree produces a set number of seeds each time-step, f_i. All trees uniformly disperse a portion of their seeds (D) among patches and retain the remaining portion of their seeds ($1-D$) on the local patch. Therefore, if p_i is the proportion of trees of species i in the population, species i disperses $f_i p_i D$ seeds to each heterospecific patch and $f_i\big[(1-D)+p_i D\big]$ seeds to each conspecific patch. **(2)** Offspring of species i at location x, after dispersal, experience density-independent mortality at rate m_i' and experience specialized predation pressure at rate $a' F_{i,k}(x)$. a' is a constant that defines the baseline rate of specialized predation pressure and $F_{i,k}(x)$ defines how the spatial distribution of adult trees of species i affects conspecific offspring predation on a patch occupied by species k at location x. $F_{i,k}(x)$ is the functional form of specialized predation pressure which may be defined as the Additive−Distance-decay

(AD) model, the Additive–Fixed-distance (AF) model, the Non-additive–Distance-decay (ND) model, or the Non-additive–Fixed-distance (NF) model. The reader may refer to the main text and Appendices A-D for more details on the models.

In any case, $F_{i,k}(x)$ is essentially a metric of the number of natural enemies at location x that attack species i. The key point is that $F_{i,k}(x)$ represents how the distance and density of conspecific trees affect the *rate* at which predation occurs. Let $S_{i,k}(x,t)$ represent the number of offspring of species i on a patch occupied by species k at location x and time t. Post dispersal,

$$\frac{dS_{i,k}(x,t)}{dt} = -S_{i,k}(x,t)\big(a'F(x) + m_i'\big) \tag{E.1}$$

Solving the ODE yields

$$S_{i,k}(x,\tau) = S_{i,k}(x,0)e^{-m_i}e^{-aF_{i,k}(x)} \tag{E.2}$$

in which $a = a'\tau$ and $m_i = m_i'\tau$ where τ is the time over which predation occurs (in essence, the time over which offspring develop and are susceptible to natural enemies and other sources of mortality) and $S_{i,k}(x,0)$ is the initial offspring abundance (at $t=0$). If $k = i$

$$S_{i,i}(x,0) = f_i\big[(1-D) + p_iD\big] \tag{E.3}$$

such that

$$S_{i,i}(x,\tau) = \big[(1-D) + p_iD\big]f_ie^{-m_i}e^{-aF_{i,i}(x)} \tag{E.4}$$

If $k \neq i$,

$$S_{i,k}(x,0) = f_ip_iD. \tag{E.5}$$

such that

$$S_{i,k}(x,\tau) = p_iDf_ie^{-m_i}e^{-aF_{i,k}(x)} \tag{E.6}$$

Henceforth, I drop the τ from the notation such that

$$S_{i,i}(x) = \big[(1-D) + p_i D\big] f_i e^{-m_i} e^{-aF_{i,i}(x)} \tag{E.7}$$

and

$$S_{i,k}(x) = p_i D f_i e^{-m_i} e^{-aF_{i,k}(x)} \tag{E.8}$$

For both $S_{i,i}(x)$ and $S_{i,k}(x)$, offspring abundance is proportional to $f_i e^{-m_i}$. Therefore, "intrinsic fitness" is defined by

$$f_i e^{-m_i} = Y_i \tag{E.9}$$

Intrinsic fitness is then (explicitly) a compound trait of fecundity and survivorship, which represents the number of expected offspring in the absence of JCEs.

Finally, let $J_{i,i}(x) = e^{-aF_{i,i}(x)}$ and $J_{i,k}(x) = e^{-aF_{i,k}(x)}$. $J_{i,i}(x)$ and $J_{i,k}(x)$ are functions that describe the proportion of offspring of species i that survive JCEs on a patch occupied by species i and k, respectively ($i \neq k$) at location x. Putting it all together, the number of each species' offspring on each patch type are equal to

$$\begin{aligned} S_{i,i}(x) &= Y_i\big[(1-D) + p_i D\big] J_{i,i}(x) \\ S_{i,k}(x) &= Y_i p_i D J_{i,k}(x) \\ S_{all,i}(x) &= \sum_{n=1}^{N} S_{n,i}(x) \end{aligned} \tag{E.10}$$

where $i \neq k$. **(3)** If the adult in the focal patch at location x dies during the time step, then a lottery determines which species replaces the adult. If the adult occupying the patch does not die during the time-step, it is assumed all the offspring on the patch die. Equation (E.10) is identical to equation (1) from the main text.

3 Normalizations

In this section, I discuss the inter-model normalizations in detail.

3.1 Normalization of distance-decay and fixed-distance functional forms

Two distance-dependent functional forms are considered in the main text of the model: the fixed-distance functional form (in which offspring experience predation pressure at a fixed rate up until a distance of r meters from a conspecific adult) and the distance-decay functional form (in which the predation pressure offspring experience from a conspecific adult decays exponentially with distance). To ensure model comparisons are fair, it is necessary to set equal the how each tree induces over space between functional forms. In other words, the predation a tree induces within the radius r under the fixed distance (AF and NF) models must be set equal to the predation over the scale set by v under the distance-decay (AD and ND) models. Conceptually, this is analogous to assuming that the number of natural enemies is maintained between distance-dependent functional forms, but are distributed in space differently.

Consider a single tree from which natural enemies disperse and induce predation pressure as defined by $G(x)$ (analogous to $F_{i,k}(x)$ in the above section, except from the perspective from the adult tree rather than the offspring; I use $G(x)$ instead of $F(x)$ to avoid confusion). $G(x)$, in essence, represents the relative density of natural enemies that disperse from a focal tree to a location x meters away from it. $G(x) \leq 1$ such that $G(x) = 1$ corresponds to maximum predation pressure induced by a single adult. For the fixed distance model, $G(x) = 1$ if $x \leq r$ and $G(x) = 0$ if $x > r$. For the distance-decay model, $G(x) = e^{-x/v}$. Predation pressure is induced in 2-dimensional space such that the total predation pressure induced by a single adult is

$$g \int_0^\infty 2\pi x G(x) \mathrm{d}x \tag{E.11}$$

Note that the $2\pi x$ term reflects that fact that predation is equal to the sum (integral) of predation pressure spanned by the radius x in two dimensions.

For the fixed distance model:

$$\begin{aligned} g\int_0^\infty 2\pi x G(x)\mathrm{d}x &= g\int_0^r 2\pi x \mathrm{d}x \\ &= \pi g r^2 \end{aligned} \tag{E.12}$$

and, for the distance-decay model:

$$\begin{aligned} g\int_0^\infty 2\pi x G(x)\mathrm{d}x &= g\int_0^\infty 2\pi x e^{-x/v} \mathrm{d}x \\ &= 2\pi g v^2 \end{aligned} \tag{E.13}$$

Predation pressure is therefore equal if $g\pi r^2 = 2g\pi v^2$; canceling terms yields the relationship $r = v\sqrt{2}$. If this equality is satisfied, an individual tree induces the same total magnitude of predation pressure irrespective of how predation decays with distance (albeit distributed over space differently). Note that $g\pi r^2 = E_F$ and $2g\pi v^2 = E_D$. Therefore, this normalization is equivalent to setting $E_F = E_D$.

Note that a is not included in the normalization – predation pressure is proportional to a where JCEs occur, but a is not directly related to the distance-decay component of the functional form. Thus, the above should be interpreted as normalizing r with v.

3.1.1 Connection to previous literature

It is noted in the main text that previous papers such as Adler & Muller-Landau (2005) and Sedio & Ostling (2013) also normalize predation pressure using a mathematically (and conceptually) similar method. I briefly articulate the similarity here. Making some modest simplifications and altering some notation for consistency, I describe how they model predation.

First, they introduce the function

$$h(x) = \frac{1}{2\pi v^2} e^{-x/v} \tag{E.14}$$

where $h(x)$ is the relative density of natural enemies at distance x from the focal tree and v is identical to v in the present study. $h(x)$ integrates to 1. Then, Adler & Muller-Landau (2005) and Sedio & Ostling (2013) assume that offspring survival on a patch is a hyperbolically decreasing function of distance-weighted adult density (defined by $h(x)$) such that:

$$\Pr[\text{Survival}] = \frac{1}{1 + \beta N \sum_{m=1}^{N_i} h(x_m)} \tag{E.15}$$

where N_i is the number of adults of species i in the population and x_m is the distance of the m_{th} individual of species i to the location x, β defines the strength of predication pressure, and N acts as a general density term such that morality increases with the total number of adults in the population. This is similar to the present study, in which survival is an exponentially decreasing function (rather than hyperbolic). However, the models are conceptually and mathematically very similar.

The key point is that Adler & Muller-Landau (2005) and Sedio & Ostling (2013) vary v between simulations. Because $h(x)$ is structured such that its integral over space sums to 1, it follows varying v does not change the total number of natural enemies. In this way, they normalize the distance-dependent component of their assumed functional form. In the present study, I do not normalize v between simulations. Rather, I compare the distance-decay and fixed-distance functional forms. In the present study, $h(x)$ is analogous to

$$G(x) = e^{-x/v} \tag{E.16}$$

for the distance-decay models, which integrates to $2\pi v^2$; for the fixed-distance models, $h(x)$ is analogous to

$$G(x) = \begin{cases} 1, & \text{if } r \leq x \\ 0, & \text{if } r > x \end{cases} \tag{E.17}$$

which integrates to πr^2. Therefore, setting $2\pi v^2 = \pi r^2$ serves the same function as ensuring that $h(x)$ integrates to 1 with respect to different values of v in Adler & Muller-Landau (2005) and Sedio & Ostling (2013). This, in turn, is identical to assuming that each distance-dependent functional form produces the same total number of natural enemies that disperse from adult trees, albeit distributed in space differently. Therefore, this normalization in the present study is biologically meaningful and consistent with previous literature.

It should be noted, however, that the coefficients that determine the baseline strength of predation pressure (a in the present study and β in Adler & Muller-Landau (2005) and Sedio & Ostling (2013)) have slightly different interpretations.

3.2 Normalization of additive and non-additive functional forms

In this section, I describe the process of normalizing predation pressure between the additive and non-additive functional forms. Because offspring experience mean greater predation pressure under the additive models than under the non-additive models, it is necessary to adjust the baseline predation pressure of the latter to compensate. I take the following approach: I calculate the mean predation pressure experienced by offspring in a community given that predation is additive. Then, I adjust the non-additive model such that both models exhibit the same mean predation pressure. Here, I present the normalization and then I justify it with reference to its implementation for the non-additive models (the non-additive−fixed-distance (NF) model and the non-additive−distance-decay (ND) model).

The normalization is as follows. Let a_{A} and a_{n} be the predation pressure for an additive model and non-additive model, respectively. Additionally, let E represent E_n or E_D (recalling that these quantities are identical due to the normalization described in the above section). The additive−non-additive normalization sets a_{n} as

$$a_{\mathrm{n}} = a_{\mathrm{A}}\left(1 + \frac{E}{N}\right) \tag{E.18}$$

where N is the number of species in the community.

Now, I describe the logic behind this normalization in the context of the NF and ND models, respectively. For the NF model, if species i experiences JCEs on a given patch because it falls within the radius defined by r, it experiences predation pressure with intensity a. However, if predation pressure were additive, it would experience predation pressure additional predation pressure equal to the total number of conspecifics found within the effect radius, r. On average, one expects an additional p_iE_F offspring to fall within the patch (on the basis that p_i is the proprotion of species i in the population and E_F is the expected number of trees in the effect area defined by r). Therefore, the expected additional predation pressure induced by species i due to additive predation is equal to

$$a_{\mathrm{A}}p_iE_F \tag{E.19}$$

in which case, the total expected predation pressure for species i on a patch in which it experiences JCEs is equal to

$$a_{\mathrm{A}}\left(1 + p_iE_F\right) \tag{E.20}$$

The $+1$ comes from the baseline predation pressure that species i experiences due to the first adult falling within r and the p_iE_F is the expected additional predation due to additive effects. Thus, the above should be interpreted as "given offspring of species i experience JCEs on a patch,

what is the expected total predation pressure it experiences when predation is additive?".

Then, the mean predation pressure within the community is equal to

$$\begin{aligned}\mathbb{E}\left[a_{\mathrm{A}}\Big(1+p_iE_F\Big)\right] &= \frac{1}{N}\sum_{i=1}^{N}a_{\mathrm{A}}\Big(1+p_iE_F\Big)\\ &= a_{\mathrm{A}}\left(1+\frac{E_F}{N}\right)\end{aligned} \tag{E.21}$$

noting that the above results comes from the fact that $\mathbb{E}\left[p_i\right] = 1/N$ (the mean proportion in a community of N species is $1/N$). Therefore, the normalization requires that

$$a_{\mathrm{n}} = a_{\mathrm{A}}\left(1+\frac{E_F}{N}\right) \tag{E.22}$$

recalling a_{n} is the normalized baseline predation pressure for non-additive models and a_{A} is the baseline predation pressure for the additive models.

Very similar logic applies for the ND model. On a given patch, the ND model induces predation pressure to offspring of species i on a given patch defined by the minimum distance between the patch and a conspecific adult. However, when predation is additive, the expected additional predation pressure experienced by offspring of species i is equal to $a_{\mathrm{A}}p_iE_D$. As before, the expected predation pressure due to additive predation is equal to

$$\begin{aligned}\mathbb{E}\left[a_{\mathrm{A}}p_iE_D\right] &= \frac{1}{N}\sum_{i=1}^{N}a_{\mathrm{A}}p_iE_D\\ &= a_{\mathrm{A}}\frac{E_D}{N}\end{aligned} \tag{E.23}$$

This yields the normalization

$$a_{\mathrm{n}} = a_{\mathrm{A}}\left(1+\frac{E_D}{N}\right) \tag{E.24}$$

I now describe how this is implemented comparing numerical simulations (ODEs) of the

additive and non-additive models. I perform the following operations:

1. A simulation is run for one of the additive models with a given set of parameters (i.e. σ_Y, g, a_{A}, D, and v or r.

2. The number of coexisting species for the additive model at the end of simulation (after reaching equilibrium) henceforth N_{A}, is recorded.

3. The predation pressure for the non-additive model is set to

$$a_{\mathrm{n}} = a_{\mathrm{A}}\left(1 + \frac{E}{N_{\mathrm{A}}}\right).$$

4. A simulation is run for the non-additive model and the number of coexisting species is recorded after the community reaches equilibrium.

With these steps, additive and non-additive models are compared under the condition that the *mean* predation pressure experienced by offspring is identical between simulations. More precisely, this probes the question: given an additive model maintains N_{A} species, how many species would be maintained under a non-additive model if offspring experience the same mean predation pressure non-additively?

References

Adler, F.R. & Muller-Landau, H.C. (2005). When do localized natural enemies increase species richness? *Ecol. Lett.*, 8, 438–447.

Sedio, B.E. & Ostling, A.M. (2013). How specialised must natural enemies be to facilitate coexistence among plants? *Ecol. Lett.*, 16, 995–1003.
