# Supplementary material for: The functional form of specialised predation affects whether Janzen–Connell effects can prevent competitive exclusion
Source: Ecol Lett. 2022 Apr 26;25(6):1458–70. doi: 10.1111/ele.14014 (PMC9324109; doi:10.1111/ele.14014)
Supplement: Supplementary file 6 — Supplementary Material [file ELE-25-1458-s001.pdf]

Appendix F: argument related to dispersal-limited communities

Contents

1 Dispersal limitation and species richness **2**

1.1 The effects of large a 2

1.2 The effects of small a 3

1.3 Expanded argument on dispersal limitation 3

1.3.1 Connection to Stump & Chesson (2015) 4

1.3.2 Connection to other models 5

1 Dispersal limitation and species richness

1.1 The effects of large a

In the main text, it is noted that dispersal limitation does not matter if a (baseline predation pressure) is very large. To show this, consider the invasion criteria of species i (see Appendices A-D) which can be written as

$$\frac{Y_i(1-D)e^{-a}}{Y_i(1-D)e^{-a} + D\sum_{k\neq i} Y_k p_k \,\mathbb{E}[J_{k,i}(x)]} + \\ Y_i D \sum_{m\neq i} \frac{1}{Y_m\big[(1-D)+p_m D\big]e^{-a}\,\mathbb{E}[J_{m,i}(x)] + D\sum_{k\neq m} Y_k p_k \,\mathbb{E}[J_{k,m}(x)]p_m} > 1 \tag{F.1}$$

noting that $\mathbb{E}[J_{m,m}(x)] = e^{-a}\,\mathbb{E}[J_{m,i}(x)]$ because local JCEs simply induce an addition e^{-a} mortality. In Appendices A-D, it is assumed that $D = 1$ and that the species identity of the tree previously occupying a patch does not matter such that $Y_m p_m e^{-a}\,\mathbb{E}[J_{m,i}(x)] \approx Y_m p_m \,\mathbb{E}[J_{m,i}(x)]$. Making these assumptions and doing some rearranging of equation (F.1) yields

$$Y_i \sum_{m\neq i} \frac{1}{\sum_{k\neq i} Y_k p_k \,\mathbb{E}[J_{k,i}(x)]} p_m > 1 \tag{F.2}$$

Now, I consider when there is dispersal limitation and a is large. I take the limit of equation (F.1) as $a \to \infty$. Therefore, all terms containing e^{-a} will disappear. This yields

$$Y_i \sum_{m\neq i} \frac{1}{\sum_{k\neq i,k\neq m} Y_k p_k \,\mathbb{E}[J_{k,i}(x)]} p_m > 1 \tag{F.3}$$

Comparing equations (F.2) and (F.3), the only difference is that species m is not included in the denominator for the latter equation (noting the difference in the indexes on the summations). In other words, only one less species' offspring are present on a given patch. Therefore, unless there are very few species or if one species is highly dominant, equations (F.2) and (F.3) will be

approximately the same value. Therefore, for large a such that $e^{-a} \approx 0$, the dispersal-limited case is approximately identical to that of the non-dispersal-limited case.

1.2 The effects of small a

In the main text, it is also claimed that dispersal limitation should not matter for small a. As $a \to 0$, no JCEs occur. Then, the system converges to a lottery model with no stabilizing effects (resulting in mono-dominance) in which case dispersal limitation is irrelevant. Taken with the above result, this implies that dispersal limitation is less important for both low a and very high a, consistent with Fig. 2 from the main text (at least, for the non-additive models).

1.3 Expanded argument on dispersal limitation

In the Results and Discussion of the main text, I argue that the functional form of specialized predation (whether it is additive or non-additive) interacts with dispersal limitation. Here, I present an extended argument for why dispersal limitation leads to lower species richness when predation is non-additive but not when it is additive.

Consider when predation is additive and species experience dispersal limitation ($D < 1$). Additionally, consider two conspecific adults nearby in space such that they induce predation pressure on each others patches. Each conspecific adult increases the offspring mortality of the locally dispersed offspring on each patch. More generally, as a species becomes more common in the community, its locally dispersed offspring experience greater and greater mortality due to additive predation. Therefore, dispersal limitation does not affect rare species advantage: although rare species lose some of their locally dispersed offspring to Janzen-Connell Effects (JCEs), they lose a smaller proportion than more common species lose.

In contrast, if predation is non-additive, then two conspecific adults close in proximity do not affect the survival of offspring on each others patches. More generally, as a species becomes more common in the community, the proportion of locally dispersed offspring that experience

mortality due to JCEs does not change. As a result, the survival a rare species' locally dispersed offspring is exactly the same as that of common species. Thus, relative to when there is no dispersal limitation ($D = 1$, in which case rare species' offspring experience no JCE-induced mortality) rare species advantage is diminished.

1.3.1 Connection to Stump & Chesson (2015)

I now connect the above arguments to Stump & Chesson (2015). Stump & Chesson (2015) compare two JCE-related situations: (1) when JCEs are "local", occurring directly beneath adults (only on the local patch; identical to the NF model with $E = 0$) and (2) when JCEs are entirely "non-localized". For "non-localized" JCEs, predation occurs equally on every patch in the community such that the proportion of offspring of species i that die on every patch, J_i, is equal to:

$$J_i = \alpha p_i \tag{F.4}$$

where p_i is the proportion of species i in the community and α represents the strength of JCEs ($0 \leq \alpha \leq 1$). In comparing these models, Stump & Chesson (2015) find that dispersal limitation reduces the stabilizing effects of JCEs for the "local" model, but not for the "non-local" model. They conclude that this is because locally dispersed offspring of rare species experience mortality in the "local" model, but not in the "non-local" model.

This is not incorrect, but the I argue that it's not the mortality of locally dispersed offspring that causes this result *per se*. Rather, the "non-local" model presented by Stump & Chesson (2015) is implicitly additive while their "local" model is non-additive. I aruge it is this distinction that truly underlies why dispersal limitation decreases the JCE stabilizing effect in Stump & Chesson (2015).

The "non-local" model in Stump & Chesson (2015) does not allow for any JCE-induced local offspring mortality of rare species ($J_i = \alpha p_i \to 0$ as $p_i \to 0$). In other words, all offspring of rare

species always escape JCEs. Therefore, it is ambiguous in Stump & Chesson (2015) whether additive predation or the lack of local offspring mortality underlies why the "non-local" model decreases JCE stabilizing effect strength. The additive (AD and AF) models in the present paper allow for the co-occurrence dispersal limitation such that a rare species' locally dispersed offspring experience JCE-induced mortality and additive predation simultaneously. When $D < 1$ and predation is additive (AD and AF models), species richness is indistinguishable from the $D = 1$ case (main text, Fig. 2). When $D < 1$ and predation is non-additive (ND and NF models), species richness is lower than the $D = 1$ case. The additive models incorporate "local" predation as defined in Stump & Chesson (2015) (the survival of locally dispersed offspring of rare species is decreased by JCEs) yet species richness is unaffected by dispersal limitation. This implies that the nature of predation – whether it is additive or non-additive – determines whether dispersal limitation decreases the ability of JCEs to maintain species richness (rather than "local" vs. "non-local" predation *per se*). In other words, whether JCEs kill locally dispersed offspring of rare species does not affect whether dispersal limitation decreases the ability of JCEs to stabilize coexistence – rather, what matters is if the locally dispersed offspring of rare species experience lower mortality *relative* to resident species.

1.3.2 Connection to other models

Despite the above insights, Muller-Landau & Adler (2007) find species richness can increase with dispersal limitation (particularly when predators disperse large distances). As their model incorporates additive predation, this seems to contradict the above results. Importantly, Muller-Landau & Adler (2007) examine spatially explicit models, using spatially explicit seed and predator dispersal kernels. I hypothesize the results from Muller-Landau & Adler (2007) reflect how the co-occurrence of spatially explicit seed and predator dispersal kernels modify the spatial structure of trees. Similarly, papers in which dispersal limitation increases species diversity (Detto & Muller-Landau, 2016; Wiegand *et al.*, 2021) largely attribute this result to species aggregation.

Aggregation is not possible under the model in this study (both in terms of how dispersal limitation is modeled and because the ODE assumes species are approximately randomly distributed in space). Therefore, further investigations of how dispersal and distance- and density-dependent are warranted, but beyond the scope of the present study.

References

Detto, M. & Muller-Landau, H.C. (2016). Stabilization of species coexistence in spatial models through the aggregation–segregation effect generated by local dispersal and nonspecific local interactions. *Theor. Popul. Biol.*, 112, 97–108.

Muller-Landau, H. & Adler, F. (2007). How seed dispersal affects interactions with specialized natural enemies and their contribution to the maintenance of diversity. In: *Seed dispersal: theory and its application in a changing world*. CAB International Wallingford, UK, chap. 18, pp. 407–446.

Stump, S.M. & Chesson, P. (2015). Distance-responsive predation is not necessary for the janzen–connell hypothesis. *Theor. Popul. Biol.*, 106, 60–70.

Wiegand, T., Wang, X., Anderson-Teixeira, K.J., Bourg, N.A., Cao, M., Ci, X. *et al.* (2021). Consequences of spatial patterns for coexistence in species-rich plant communities. *Nat. Ecol. Evol.*, pp. 1–9.
