# Supplementary material for: The functional form of specialised predation affects whether Janzen–Connell effects can prevent competitive exclusion
Source: Ecol Lett. 2022 Apr 26;25(6):1458–70. doi: 10.1111/ele.14014 (PMC9324109; doi:10.1111/ele.14014)
Supplement: Supplementary file 7 — Supplementary Material [file ELE-25-1458-s005.pdf]

Appendix G: Supplemental Figures

Contents

1 Introduction 2

2 Figures 3

1 Introduction

In this Appendix, I present supplemental figure (most of which are referenced in the manuscript). Figure legends provide all necessary information to interpret the figures.

2 Figures

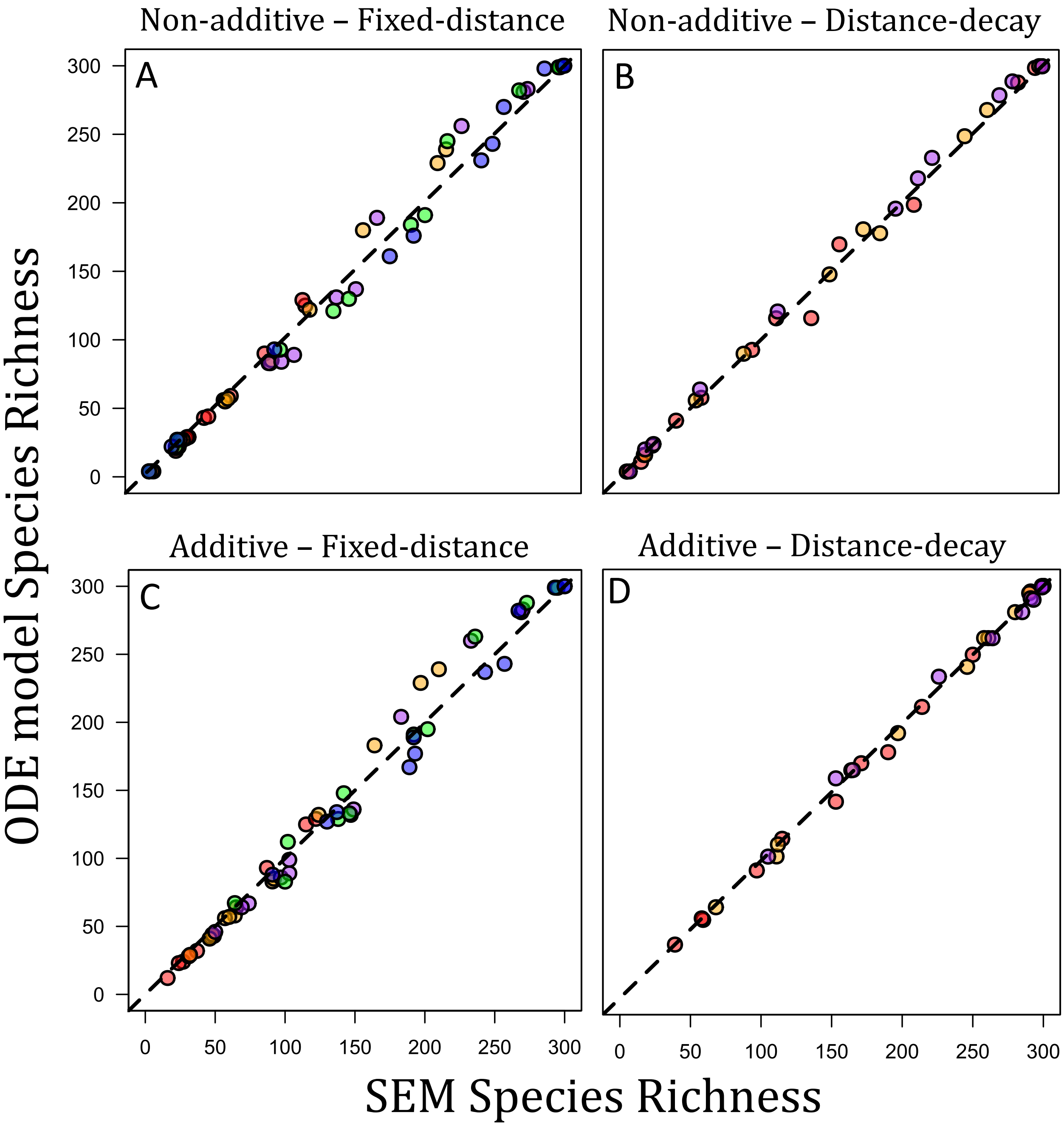

Fig. G1: Ordinary Differential Equation (ODE) model validation. The figures compare species richness between Spatially Explicit Model (SEM) and ODE model simulations under identical parameterizations. Each panel shows a different model labeled by its functional form. The dashed line is the one-to-one line (points on the line represent when the SEM and ODE yield the exact same diversity output). For panels A and C (the fixed-distance models), red points depict a 3×3 Moore Neighborhood ($E_F = 9$), orange/yellow points depict a 5×5 Moore Neighborhood ($E_F = 25$), purple points depict a 7×7 Moore Neighborhood ($E_F = 49$), green points depict a 9×9 Moore Neighborhood ($E_F = 81$), and blue points depict an 11×11 Moore Neighborhood ($E_F = 121$). This approximately corresponds to r between 4.0 and 14. For panels B and D, red points are when $v = 5$, orange/yellow points are when $v = 7.5$, and purple points are when $v = 10$. To a first approximation, the ODE model yields the same species richness output as the SEM. See Appendices A-D for more details and summary statistics.

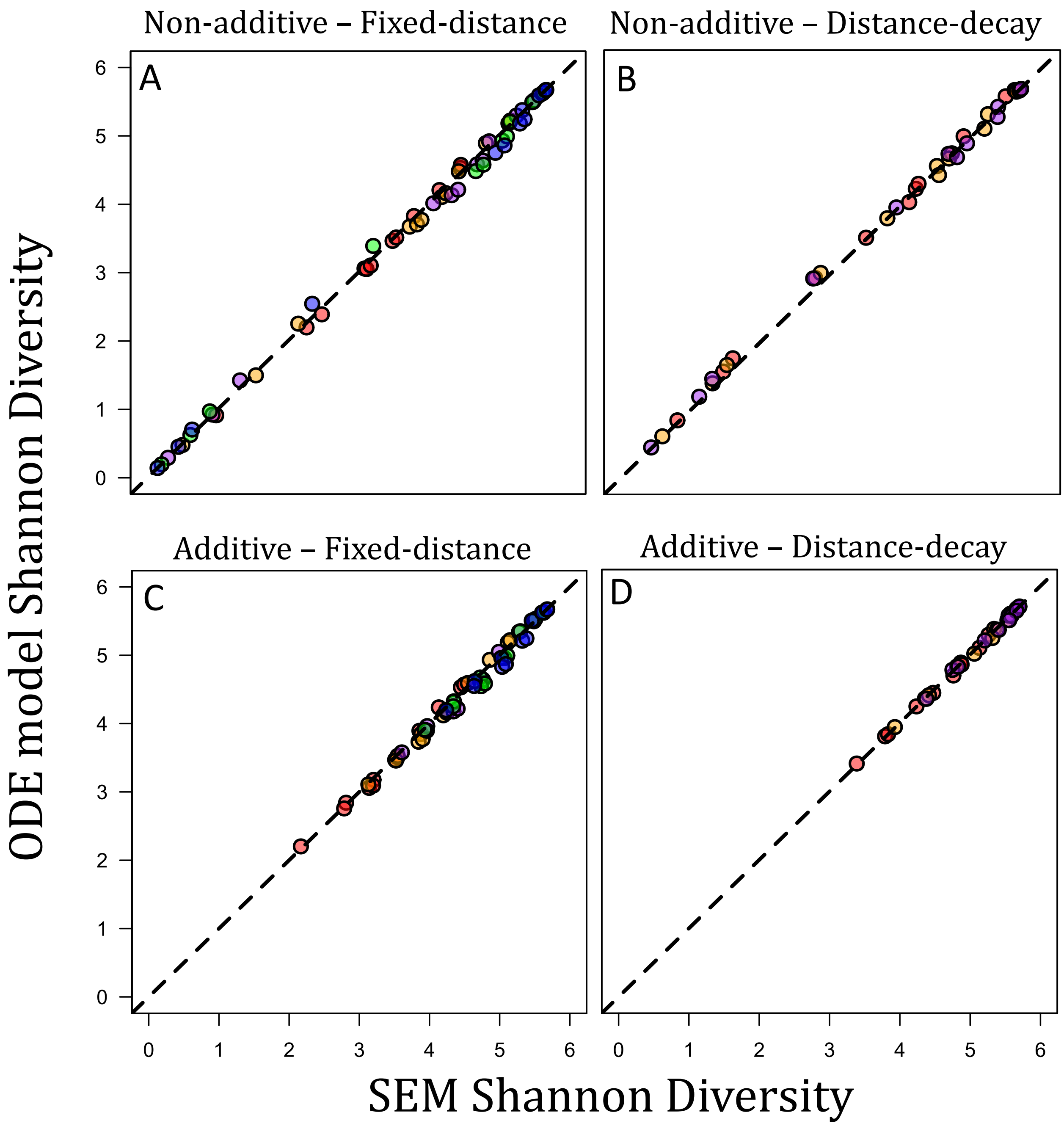

Fig. G2: The same as Fig. G1, but showing Shannon Diversity instead of species richness. SEM and ODE model outputs are highly similar.

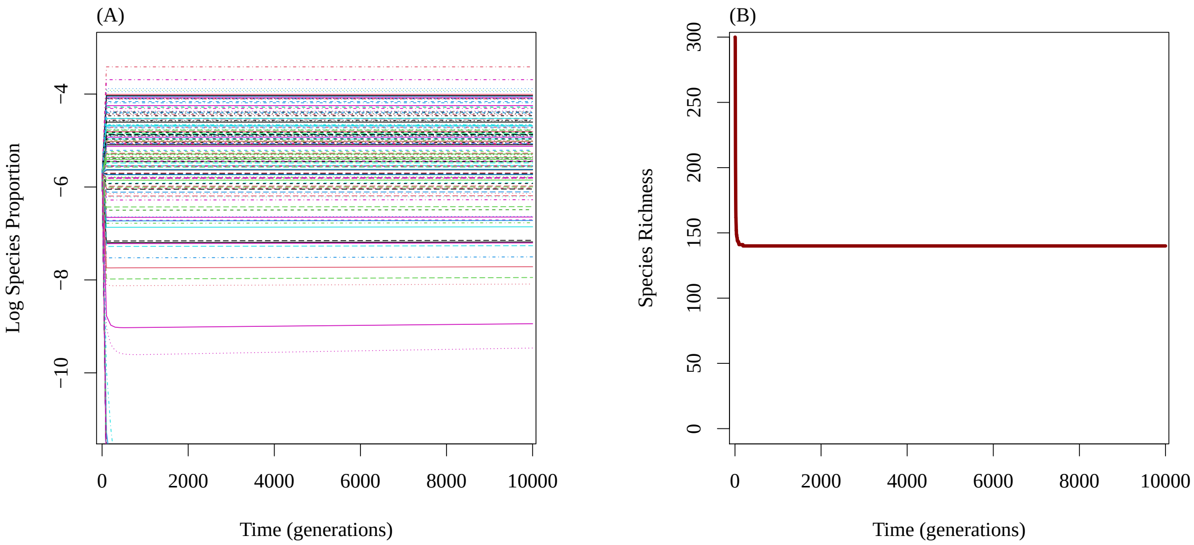

Fig. G3: An example of a time-series output from the ODE model, showing the AD model. (A) shows the log-transformed proportion of each species as a function of time. Abundances stop changing relatively early into the simulation. (B) shows number of species persisting in the community as a function of time. This implies the transient dynamics have concluded and the system is at equilibrium. This is a typical example. Parameters are as follows: $v = 10$, $g = 0.172$, $1 - e^{-a} = 0.7$ ($a \approx 1.2$), $D = 1$, $\sigma_Y = 1.0$.

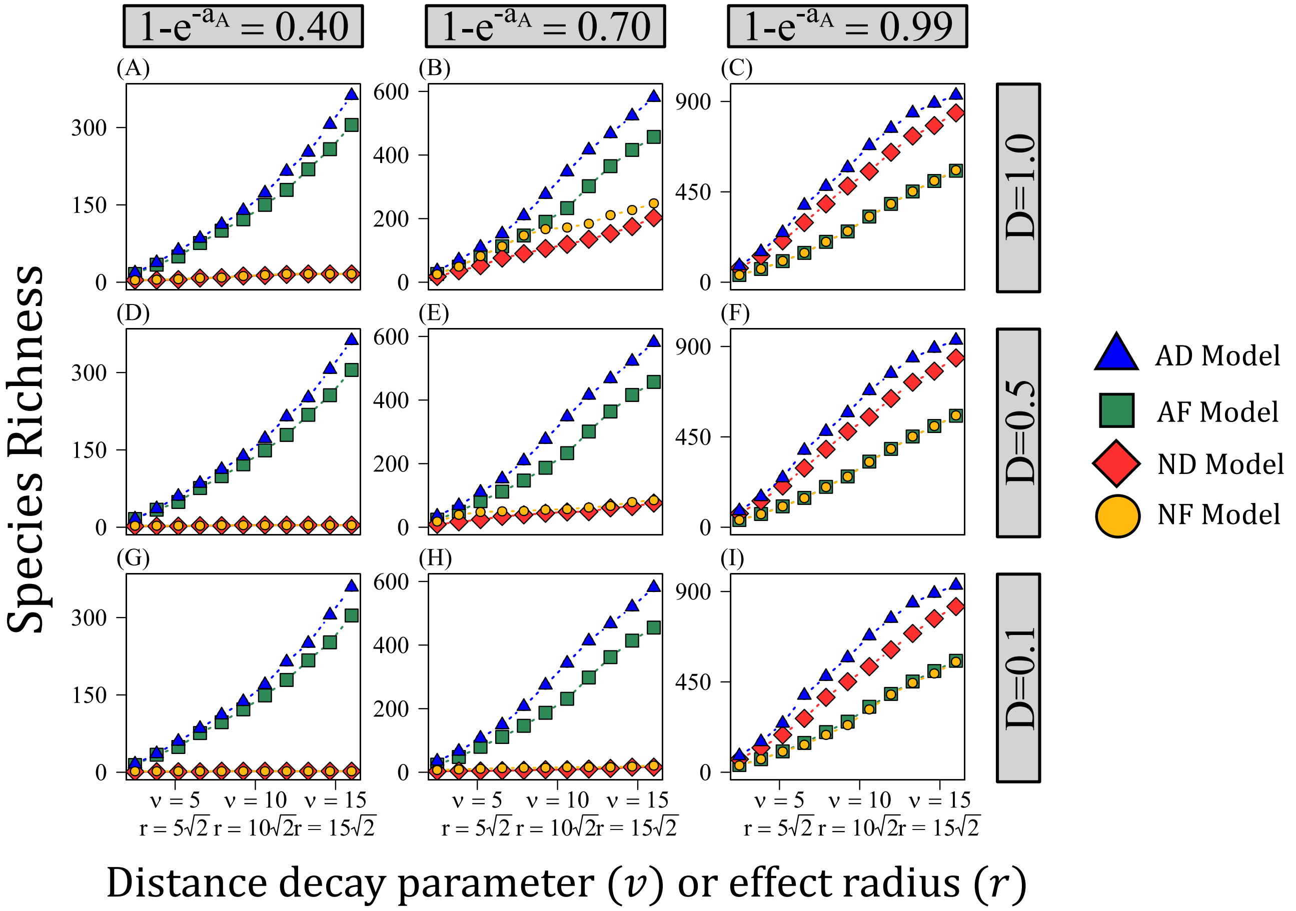

Fig. G4: The same as Fig. 2 from the main text, but with 1000 species in the initial community (instead of 300). In the main text, it is noted that model differences are more extreme for a larger initial species pool. In columns 1 and 2, the additive models can maintain hundreds of more species than the non-additive models. Similarly, in column 3, the species richness maintained by the distance-decay models is often several hundred greater than the fixed-distance models. For large a_A and large v, the species richness of the AD model is nearly 1000 species. As in Fig. 2 of the main text, $g = 0.172$ and $\sigma_Y = 0.55$ ($Y \sim$ lognormal$[\mu = 0, \sigma_Y]$). All other relevant parameters are noted on the figure.

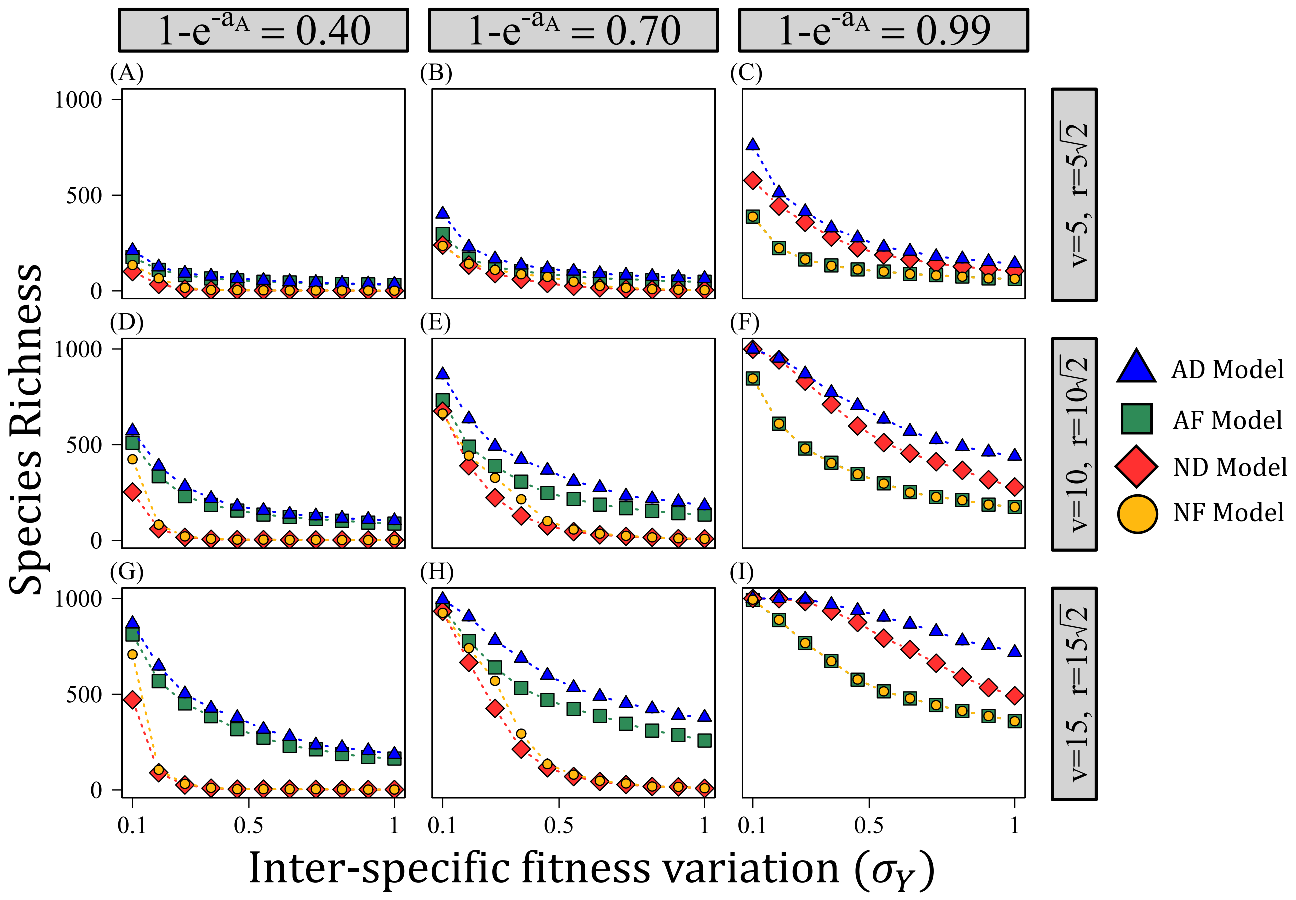

Fig. G5: The same as Fig. 3 from the main text, but with 1000 species in the initial community (instead of 300). As noted in the main text, results are qualitatively similar to the 300 species case, but the numerical difference in species richness maintained by models is larger. In particular, when JCEs extend over a large spatial scale (large v and r; rows 2 and 3) models differences are sometimes very large. The starkest contrast to Fig. 3 of the main text is Panel (I) – in this case, the AD model maintains hundreds of more species than each other model. In the analogous plot in the main text (Fig. 3, Panel (I)), all species maintain high species richness for all σ_Y. Therefore, when JCEs are very strong (large v or r and large a_A, as in Panel (I)), examining a larger species pool reveals the full extent to which the AD maintains greater species richness than the other models (particularly the fixed-distance models).

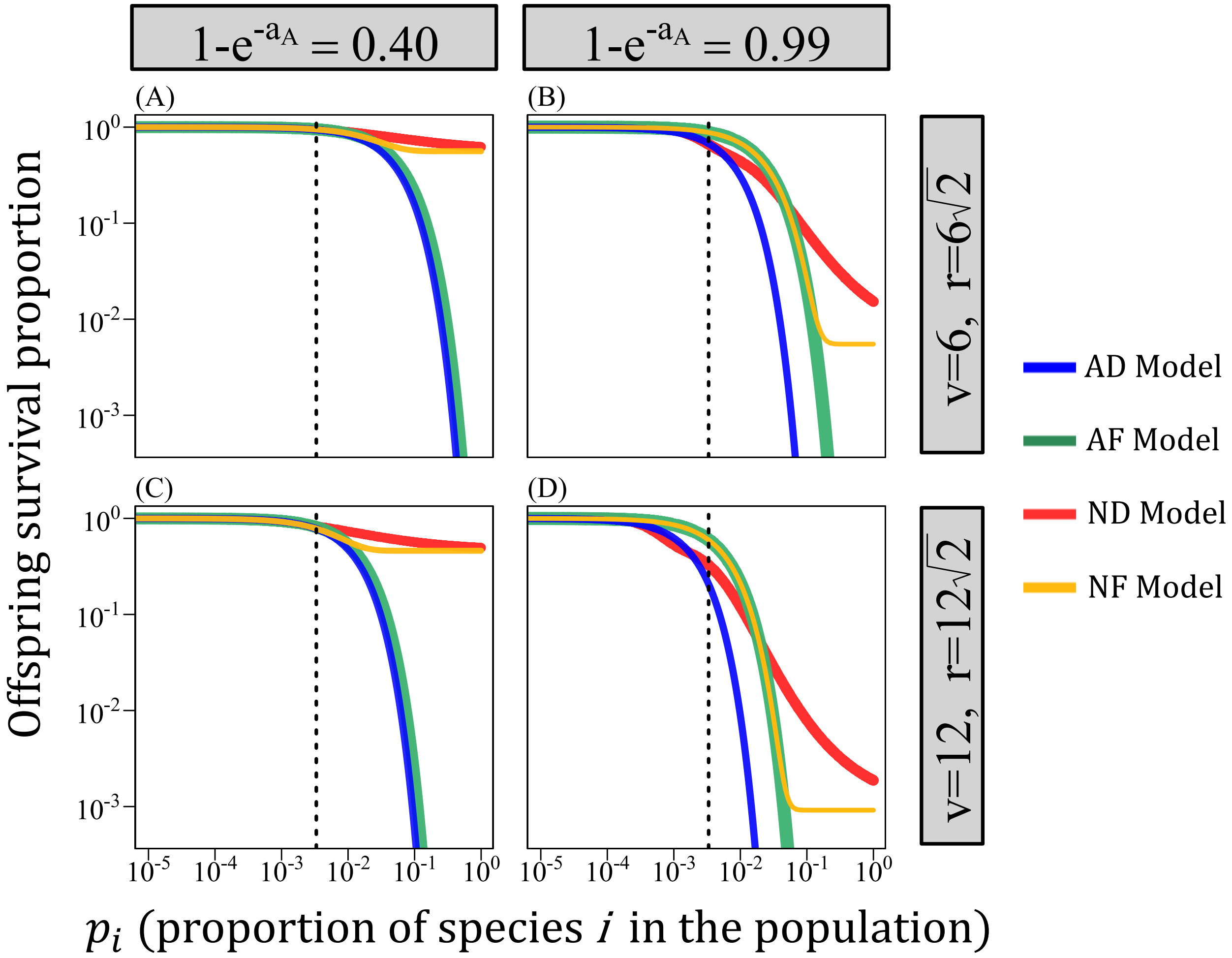

Fig. G6: The same as Fig. 4 from the main text, but both axes are log-scaled (rather than just the x-axis). This allows the observation that the additive models indeed produce stronger negative frequency dependence (lower offspring survival for large p_i) than the non-additive models in all cases, including (B) and (D) (which is difficult to see in Fig. 4 of the main text). Parameters are as follows: $g = 0.172$, $D = 1.0$, $N = 300$, and $a_{\mathrm{N}} = a_{\mathrm{A}}\big(1 + E/N\big)$ where $E = E_F = E_D = 2\pi v^2 g$.

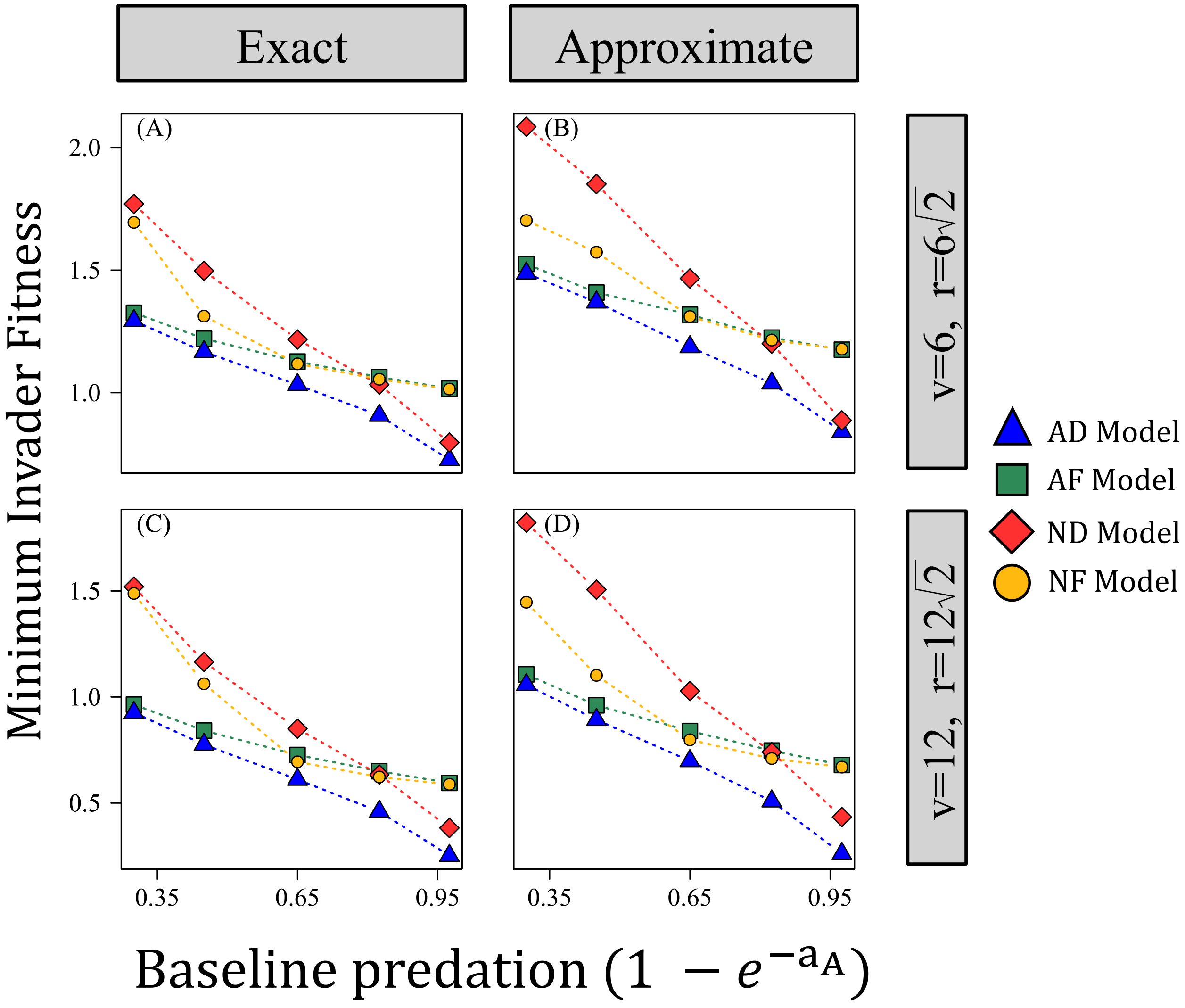

Fig. G7: The approximate invasion criteria compared to the exact invasion criteria (the right-hand-side, "minimum invader fitness", of the invasion criteria shown in Table 1). (A, C) show the exact invasion criteria for each model and (B, D) show the approximate invasion criteria for each model. Results are qualitatively identical, although approximate invasion criteria tends produce larger values (which means the approximation underestimates stabilizing strength). Relevant parameters are as follows: $D = 1$, $g = 0.172$, and $\sigma_Y = 0.375$. Other parameters are listed on the figure.

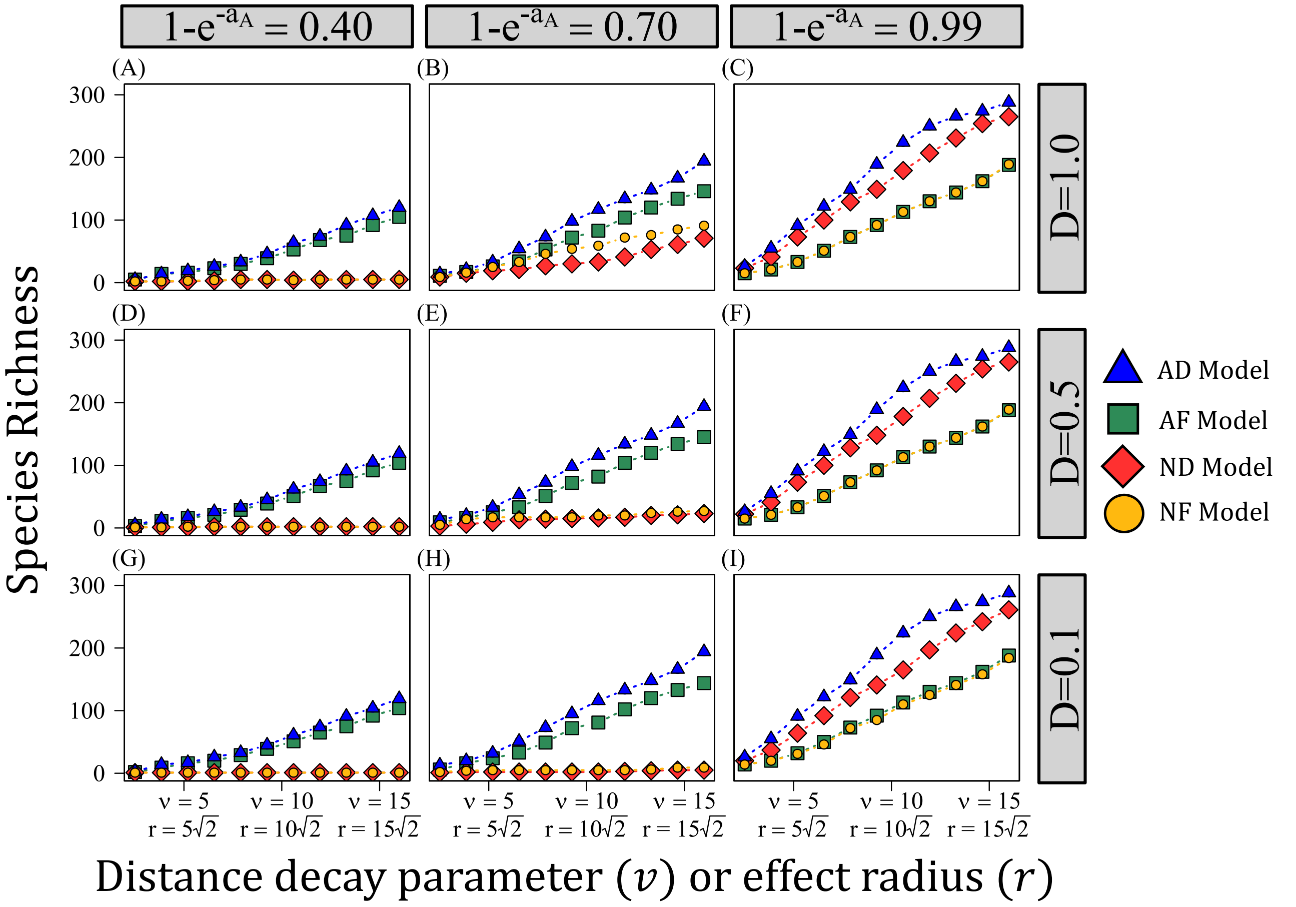

Fig. G8: Species richness maintained by each JCE functional form under different values of baseline predation pressure ($a_{\rm A}$), dispersal limitation (D), and the spatial scale of predation (v and r) at relatively low tree density ($g = 0.06$). In the main text, it is noted that lower g produces lower species diversity; this figure should be compared to Fig. 2 of the main text, as they are identical except for the value of g. For each plot, the x-axis depicts either v or r (depending on the JCE functional form) and the y-axis is species richness (the number of species maintained in the community at equilibrium). Models are differentiated by shape and color. Each column depicts a different value of $a_{\rm A}$ (the baseline predation pressure for the additive models) and each row shows a different value of D ($D = 1$, $D = 0.5$, and $D = 0.1$, respectively). The non-additive models are normalized such that $a_{\rm N} = a_{\rm A}(1 + E/N_{\rm A})$ where $a_{\rm N}$ is the normalized predation pressure for the non-additive models, $N_{\rm A}$ is the diversity maintained by the additive model to which the non-additive model is being compared and E is either E_F or E_D (which are equivalent). $a_{\rm N}$ was calculated based on the diversity maintained by the AD model for the additive – non-additive normalizations. Using the AF model outputs to quantify $a_{\rm N}$ yielded trivially similar results. All simulations were conducted with 300 species initially in the population. $g = 0.06$ and $\sigma_Y = 0.55$ ($Y \sim$ lognormal$[\mu = 0, \sigma_Y]$). All other relevant parameters are noted on the figure.
